# Effect of Different Exercise Modalities on Inflammatory Markers in Individuals with Depressive Disorder: A Systematic Review and Meta-Analysis

**DOI:** 10.3390/life15091452

**Published:** 2025-09-16

**Authors:** Jie Song, Jinning Zhang, Xijin Wang, Jiaqi Liang, Yan Li

**Affiliations:** 1 Department of Exercise Physiology, School of Sport Science, Beijing Sport University, Beijing 100084, China; 2023210317@bsu.edu.cn (J.S.); 2024210322@bsu.edu.cn (J.Z.); 2023210322@bsu.edu.cn (X.W.); 2022210362@bsu.edu.cn (J.L.); 2 Beijing Key Laboratory of Sports Performance and Skill Assessment, Beijing Sport University, Beijing 100084, China

**Keywords:** exercise modalities, depressive disorder, inflammation, meta-analysis

## Abstract

Background: One of the important mechanisms of depression is long-term high levels of inflammation. Exercise may help relieve depressive symptoms and is associated with anti-inflammatory effects. This research methodically assesses how various exercise modalities influence peripheral inflammatory markers in individuals with depression, so that more personalized and precise exercise schemes can be provided for people with depression to improve inflammation. Methods: Randomized controlled trials on depressive disorder, exercise, and inflammation published until May 2025 were screened in five databases. The Cochrane Risk of Bias tool (RoB2.0) was used to evaluate potential biases, with random effects meta-analyses gauging the impact of varying exercise regimens on peripheral inflammatory markers (CRP, IL-6 and TNF-α) involving exercise types, intensity, frequency, and length. Meta-regression analyses were employed to show the sources of heterogeneity and investigate potential moderator variables influencing CRP, IL-6, and TNF-α (PROSPERO CRD42024590612). Results: A total of 13 unique studies with 1004 participants were included. Overall, exercise training could alleviate depressive symptoms (SMD = −0.59, 95%CI: [−0.86, −0.32], I^2^ = 61.7%, *p* < 0.001), and subgroup analysis showed that exercise lasting 8–12 weeks could reduce the level of TNF-α (MD = −0.74, 95%CI: [−1.30, −0.17], I^2^ = 0, *p* < 0.05). Apparent discrepancies between subgroup and meta-regression findings were resolved by advanced modeling, which identified a significant non-linear relationship for TNF-α (quadratic term *p* = 0.003), characterized by a reduction peak at 8–12 weeks followed by a gradual increase, rather than a positive linear trend. Conclusions: Exercise could alleviate depressive symptoms in individuals with depression. However, the influence of exercise length on TNF-α levels has conflicting conclusions because of inconsistent evidence and substantial heterogeneity. Future high-quality trials with standardized biomarker measurements and better control of confounding factors are essential to determine whether the antidepressant effects of exercise are mediated by inflammation.

## 1. Introduction

Depressive disorder is a severe, persistent mental health condition. According to the World Health Organization, approximately 5% of adults worldwide are affected by depression [1]. It is projected to become the leading cause of global disease burden by 2030 [2,3]. This mental health epidemic is now a pressing issue on the global stage, posing a significant threat to human health due to its alarming prevalence, significant levels of disability, and tragic rate of suicide [4]. The complex pathogenesis of depression involves alterations in multiple physiological systems, including the neurotransmitter-receptor hypothesis, HPA axis hypothesis, and cytokine hypothesis [5,6,7]. Among various pathogenesis hypotheses, the cytokine hypothesis has emerged as a particularly significant area of research in recent years. This hypothesis suggests that inflammatory processes are involved in the onset and maintenance of depression [8]. To further investigate the role of inflammatory markers in depressive disorder, both animal experiments and human studies have been conducted. Animal experiments have shown that chronic social failure stress model mice exhibiting depression-like behavior showed more pronounced changes, including elevated plasma corticosterone, increased proinflammatory cytokines levels, impaired proliferation of T lymphocytes, and decreased hippocampal BDNF levels [9]. Similarly, human studies have consistently found that circulating levels of at least 15 markers such as CRP, IL-2, IL-6, CCL3, TNF-α, and TNF-β (also known as Lymphotoxin-alpha) were increased in patients with depression [10,11,12]. Thus, serum cytokines could be potential biomarkers for diagnosing and tracking treatment progress in major depressive disorder [13]. Critically, there is substantial evidence for bidirectional communication between central and peripheral inflammation throughout the pathogenesis of depression [14,15,16]. Peripheral proinflammatory factors can trigger a central inflammatory response through multiple pathways. First, peripheral proinflammatory factors can enter the brain through “leaky” regions of the blood–brain barrier [17,18]. Second, through a neural route, they can bind to cytokine receptors via afferent nerve fiber cytokine receptors [19]. And third, stress can upregulate the expression of ICAM-1 in the choroid plexus and, in turn, enhance the infiltration of immune cells into the central nervous system (CNS). And this infiltration then affects the immunological competence function of microglia and other CNS immune cells [20,21,22,23]. Ultimately, these peripheral proinflammatory factor-induced neuroinflammation processes in CNS can directly lead to structural and functional disruptions of neural circuits implicated in mood and cognition and contribute to the development of depressive symptoms [24,25,26,27]. Therefore, peripheral inflammation-targeted interventions hold promise as a translational therapeutic strategy for depression.

Given the limitations and side effects of pharmacological treatments, non-pharmacological interventions such as exercise therapy are increasingly important [28,29]. Numerous studies have indicated that exercise is a promising and effective non-pharmacological treatment method for depressive disorder [30,31,32,33]. A key mechanism by which exercise may exert its benefits is that exercise has a close link to inflammation and plays a dual regulatory role in modulating the inflammatory response [34,35]. Acute exercise could trigger the enhancement of reactive oxygen species (ROS) and inflammatory factor levels that temporarily harm muscle fibers, leading to muscle fibers fatigue, pain, and inflammation [36,37,38,39,40]. Moreover, contraction stimulates skeletal muscles to produce a lot of “myokines”, such as IL-6 [35,41]. This swift elevation in IL-6 levels is promptly coupled with the induction of anti-inflammatory substances (e.g., IL-10 and IL-1 receptor antagonist) [42,43]. During the recovery phase, this rise in IL-6 helps to calm down the inflammation and reduce oxidative stress post exercise [44,45]. Chronically, consistent or regular exercise training may lead to a gradual decrease in basal levels of systemic inflammation, potentially through mechanisms involving the adaptation and enhancement of anti-inflammatory pathways [46]. This long-term anti-inflammatory effect is particularly relevant for chronic inflammatory conditions similar to depression. However, findings regarding the specific impact of exercise training on proinflammatory factors in individuals with depression are controversial [47,48,49,50]. For example, previous studies have demonstrated that both low-intensity aerobic exercise and high-intensity interval exercise can significantly improve depressive symptoms [49,51]. However, a significant reduction in the levels of peripheral proinflammatory factors has not been consistently observed across all trials and is primarily associated with low-intensity exercise interventions in depressed individuals [51,52]. These discrepancies suggest that the influences of exercise regimens on peripheral inflammation in depression might be modulated by the type, intensity, frequency, and length of exercise [53,54]. A recent meta-analysis has demonstrated that antidepressants combined with physical exercise is an effective non-pharmacological intervention for individuals with depression to improve depressive symptoms and reduce peripheral proinflammatory factors [55]. Despite these advances, critical gaps remain. Previous studies have failed to establish a clear quantitative dose–response relationship between specific exercise regimens (e.g., type, intensity, length, and frequency) and the magnitude of reductions in peripheral inflammation markers, particularly in depressed populations, creating a significant gap in developing targeted exercise protocols for individuals with depressive disorders [56,57]. To directly address this critical gap and provide actionable insights for clinical practice, this meta-analysis systematically quantifies the effects of various exercise intervention characteristics (defined by their specific parameters) on proinflammatory biomarkers in individuals with depression, aiming to elucidate potential dose–response relationships.

## 2. Materials and Methods

The methodology for this present study followed the Preferred Reporting Items for Systematic Reviews and Meta-Analyses (PRISMA) guidelines 2020 [58], and the meta-analysis was registered in the International Prospective Register of Systematic Reviews (PROSPERO, http://www.crd.york.ac.uk/PROSPERO/) on 14 September 2024 (CRD42024590612) (the protocol is available at https://www.crd.york.ac.uk/PROSPERO/view/CRD42024590612 (accessed on 28 August 2025)). A completed PRISMA checklist is available in Supplementary File S2.

### 2.1. Inclusion and Exclusion Criteria

#### 2.1.1. Inclusion Criteria and Rationale

All studies incorporated into this meta-analysis had to satisfy specific PICOS criteria (Participants, Interventions, Comparisons, Outcomes, and Study Design). The inclusion requirements were as follows: (1) Participants of the exercise group had to be adults aged 18 or older, regardless of gender. This included individuals with a formal diagnosis of depressive disorder based on the DSM-5™ Diagnostic Criteria reference guide or ICD classification [4] or individuals from other clinical populations (e.g., those with chronic physical or neurological conditions such as COPD or Alzheimer’s disease) who presented with clinically significant depressive symptoms or a comorbid depressive diagnosis. These diagnoses were confirmed either by licensed psychiatrists or through validated assessment tools. (2) The experimental group received regular exercise as a standalone treatment, defined as structed interventions meeting core parameters: intensity at light intensity (57–63% HRmax), moderate intensity (64–76% HRmax), high intensity (77–95% HRmax) [59]; frequency of ≥1 sessions/week; session duration type-specified as ≥30 min for aerobic exercise, ≥20 min for high-intensity interval training (including recovery), or full major muscle group coverage (≥2 sets/muscle) for resistance training; and total length of ≥4 weeks. This definition integrates ACSM FITT-VP prescription principles [59]. (3) The control group comprised individuals with a depression diagnosis who were not involved in any exercise-based intervention programs, including standard care, a waiting-list control, or placebo treatments, to serve as the non-exercise control. These control conditions could be active (e.g., stretching, relaxation techniques) or passive (e.g., placebo). When investigating the effects of pan-population exercise or the antidepressant effect of different volumes, a control group consisting of healthy subjects or DD individuals who received exercise intervention was acceptable. (4) The study focused chiefly on measuring depressive symptoms and inflammatory marker levels as its main endpoints. Additionally, it examined secondary outcomes, including how various forms of exercise, workout frequency, and session duration influenced both depression and anxiety symptoms, as well as inflammatory marker concentrations. (5) Only randomized controlled trials (RCTs) published in peer-reviewed journals or academic dissertations were considered; conference abstracts and presentations were excluded from the analysis.

#### 2.1.2. Exclusion Criteria

The meta-analysis excluded studies if they fell into any of the following criteria: (1) Clinical trials that were ongoing or had initiated physical activity programs for expectant or nursing mothers. (2) Trials that incorporated nutritional advice as a comparative intervention. (3) Research studies including individuals with a clinical diagnosis of anxiety or bipolar disorder. (4) Studies that were short on details about the specifics of exercise—such as its frequency, intensity, duration, volume, and progression. (5) Animal studies, conference reports, review papers, editorials, and non-English language articles. (6) The experimental group used acute exercise intervention. (7) The data is incomplete and the data cannot be converted.

### 2.2. Search Strategy

The protocol for this research was developed in advance and officially registered with PROSPERO (CRD42024590612). The review was conducted in accordance with the Preferred Reporting Items for Systematic Review and Meta-Analyses (PRISMA) statement [60]. To gather pertinent studies published up to May 2025, searches were conducted across some databases including PubMed, the Cochrane Library, Embase, EBSCO, and Web of Science. The search incorporated keywords related to “depressive disorder”, “exercise”, and “inflammation” to ensure a comprehensive collection of relevant records. Details of the search strategy and findings are available in Supplementary Table S1.

### 2.3. Quality Assessment

To evaluate the methodological rigor of the studies in our analysis, we employed the RoB 2.0 tool [61]. Each study was classified into one of three categories, “low risk of bias”, “high risk of bias”, or “some concerns”, across five critical domains: (1) randomization process; (2) deviations from the intended interventions; (3) missing outcome data; (4) measurement of the outcome; and (5) selection of the reported result [62]. Two independent evaluators (JS and XJW) performed the assessments following the Cochrane RoB 2.0 guidelines, issuing individual ratings for every study. In cases where discrepancies arose, a third reviewer (YL) stepped in to mediate discussions until a unanimous decision was reached.

### 2.4. Data Extraction

In each reviewed study, pertinent details were extracted, including the following: (1) Study characteristics (title, author information, publication year). (2) Participants’ characteristics (sex, age and sample size). (3) Clinical features (depression diagnostic criteria baseline depression severity using standard scales, e.g., HAM-D/BDI). (4) Exercise intervention parameters: exercise type (e.g., mixed exercise [63], traditional aerobic exercise [64], Mind–Body exercise [65], and high-intensity interval exercise [66]). These categories were selected because they represent classifications commonly used by health promotion organizations and are applicable to the exercise modalities adopted in the included studies. For this study, aerobic exercise was defined as any exercise intervention aimed at improving cardiovascular health. This includes walking, running, dancing, cycling, swimming, etc. [67]. Mind–Body exercise was categorized as exercise combining movement sequences, breath control, and attention regulation. Examples of Mind–Body exercise are Tai Chi, Pilates, and yoga [68]. High-intensity interval training was defined as repeated bouts of relatively high-intensity exercise interspersed with easier recovery periods or rest [66]. Mixed exercise is defined as a combination of at least two modalities; exercise intensity (light intensity: 57–63% HRmax, moderate intensity: 64–76% HRmax, high intensity: 77–95% HRmax [59]); exercise frequency; exercise length. (5) Inflammatory marker levels (some specific biomarkers (e.g., CRP, IL-6, TNF-α), and pre/post-intervention mean ± SD or change values). These makers were selected because they were the most commonly reported across the included studies, whereas other inflammatory markers (e.g., IL-1 α; IL-1 β; IL-2) were reported in very few studies. (6) Outcome measures (Main outcome: The average difference in the alteration of biomarker concentrations, from the pre-intervention to the post-intervention period, between those in the exercise group and the control group, following a sustained exercise program. Secondary outcome: Measures of depressive symptoms. Studies that provided biomarker data of patients with depressive disorder were included, even if accurate information on depressive symptoms was not available.).

### 2.5. Data Synthesis and Analysis

For depressive symptoms, outcomes were expressed as standardized mean differences (SMDs) with 95% CIs, whereas mean differences (MDs) were used for the three common inflammatory factors (CRP, IL-6, TNF-α). Biomarker units (CRP in mg/L and TNF-α and IL-6 in pg/mL) were harmonized into international standard units, and standard errors were harmonized to SDs.

Random effects meta-analyses were fitted using restricted maximum likelihood (REML) for τ^2^ estimation. Inference was based on the Hartung–Knapp–Sidik–Jonkman (HKSJ) adjustment, which employs a t-distribution with degrees of freedom (df) = k − p. Fixed-effect models were employed only when heterogeneity was negligible (I^2^ < 50%).

To assess the robustness of the results and identify potential sources of heterogeneity, leave-one-out sensitivity analysis was conducted. A prespecified influence diagnostics panel was used to identify studies with undue influence: studentized residuals (|z| > 2 flagged as outliers), hat values (high leverage > 2/k), and Cook’s distances (high influence > 4/(k − 1)). Studies flagged by any diagnostic were subjected to influence-robust sensitivity re-analyses under the HKSJ framework.

In addition, subgroup analyses were performed to examine the effects of specific exercise prescriptions and participant characteristics on individuals with depression. The selection of these analytical variables was based on the characteristics of the included literature, which suggests that the effects of exercise interventions may be influenced by the exercise parameters themselves (such as exercise type, intensity, frequency, length, etc.) and the baseline characteristics of the subjects (such as severity of depression, age, the proportion of females, presence or absence of comorbidities, etc.).

To further explore potential sources of heterogeneity and the influence of continuous moderators (e.g., exercise parameters, participant characteristics), univariable meta-regression analyses were performed using the random effects framework with REML for τ^2^ estimation and the HKSJ method for inference. To enhance statistical power for identifying moderators common across the inflammatory response, exploratory analysis was conducted using a unified meta-regression model that combined all three inflammatory biomarkers (CRP, IL-6, TNF-α; total k = 21). This model included key exercise parameters and participant characteristics as covariates, with collinearity assessed via variance inflation factors (VIF > 4 indicated concern). The goal of this model was to assess the independent contributions of these key covariates while adjusting for biomarker type. For TNF-α, the non-linear relationship with intervention length was formally tested using the following approaches: quadratic terms, restricted cubic splines (RCS; with 3 knots at the 10th, 50th, and 90th percentiles), and categorical models (<8, 8–12, >12 weeks). Model fit was compared using AIC/BIC and Wald or likelihood-ratio tests.

Publication bias was evaluated using funnel plots and Egger’s regression test, but only when ≥10 studies were available for a given outcome, as recommended by the Cochrane guidelines. All tests were two-tailed (α = 0.05) and implemented in R software (version 4.2.0), using the metafor, dplyr, ggplot2, and knitr packages.

## 3. Results

### 3.1. The Process of Study Selection

**Figure 1:**
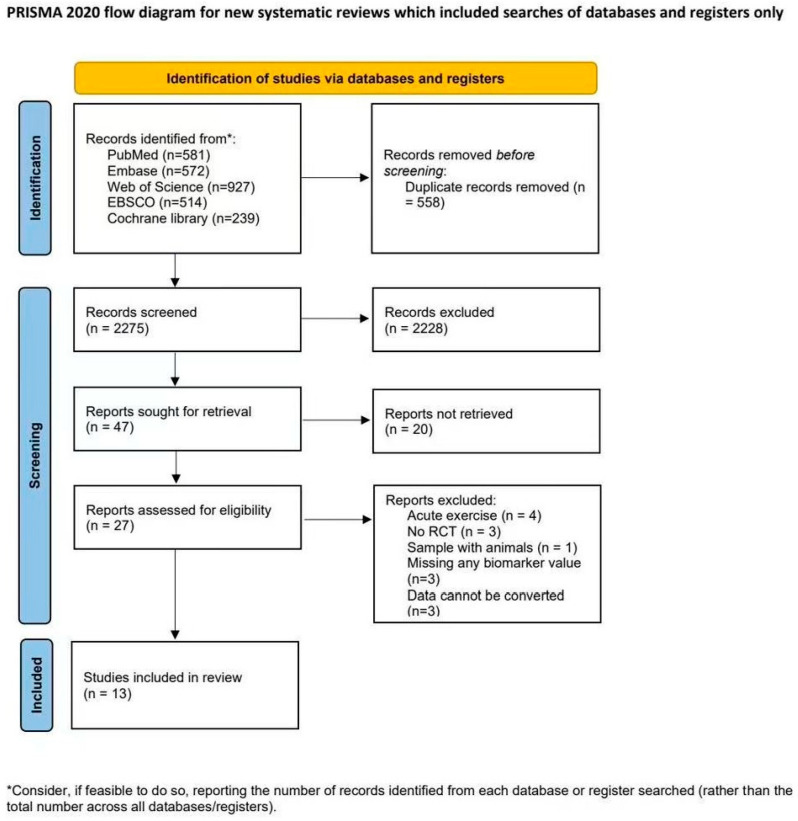
PRISMA flowchart of study selection. Flowchart adapted from PRISMA 2020 statement.

The initial search yielded a total of 2833 potentially eligible reports. After duplicate entries were eliminated, 2228 titles and abstracts were carefully reviewed. In the next stage, 27 studies were chosen for full-text reading, and of these, 14 studies were excluded, leaving 13 studies that fulfilled the inclusion criteria and were included in the present review [49,50,51,52,69,70,71,72,73,74,75,76,77], as shown in Figure 1. Conducting a manual search through the references did not uncover any further relevant studies for inclusion.

### 3.2. Characteristics of Included Studies

A summary of the key characteristics of the 13 studies included in our review is presented in Table 1. And details of intervention delivery across studies are summarized in Table S2, including settings, participant residence, supervision modes, and implementation of group exercise. A total of 1004 subjects were evenly split between the exercise group (n = 502) and the control group (n = 502), with 54.28% women in these studies. Participant ages ranged from 18 to 72 years old. There were no significant differences in gender distribution or age range between the exercise and control groups. The studies involved participants with various comorbidities, including Alzheimer’s disease [69], sleep disorders, type 2 diabetes mellitus [76], Stage C symptomatic heart failure (both preserved [HFpEF] and reduced [HFrEF] ejection fraction subtypes) [72], obstructive pulmonary disease [70], and sarcopenia [74]. Diverse exercise intervention types were employed across the qualifying studies, including mixed exercise, traditional aerobic exercise (running, cycling, and swimming), Mind–Body exercise (Qigong, Tai Chi), and high-intensity interval exercise. Of the total interventions, 11 studies used aerobic exercise and 2 studies used anaerobic exercise. Exercise intensity was categorized as low, moderate, or high. The lengths of the exercise interventions ranged from 4 weeks to 48 weeks. The control group conditions reported were active and passive, considering usual care [72,74], wait-list [49,50,51,69,70], placebo intervention [75], stretching exercise [73], health education [71], and patient-centered counseling in the group [76]. Furthermore, because the study aim to explore the influence of exercise across different populations and the antidepressant effect of different exercise volumes, the control group received an intervention of active week x passive week [77] or 4KKW exercise volume [52]. Most of the research used outcome indicators such as depression and anxiety scales and CRP, IL-6, and TNF-α levels.

### 3.3. Description of Exercise Interventions

Aerobic exercise emerged as the most extensively researched form of exercise, featuring in 11 RCTs (84.62%). In contrast, anaerobic exercise was the focus of merely two RCTs, representing just 15.38% of the research.

The aerobic exercise programs across these studies displayed considerable variation in types, frequency, length, and intensity. Usually, participants engaged in workouts 3 to 5 days each week, with intervention lengths ranging from 4 weeks to 48 weeks. Each session generally spanned between 30 and 60 min. The intensity of aerobic exercise was often determined using different heart rate per minute assessments. Only a single study reported an exercise progression plan, where exercise intensity increased from 60 to 70% in the initial two weeks to 70–80% during the final ten weeks [70]. Meanwhile, numerous other studies failed to specify how much progression occurred or when exactly the interventions took place. The types of aerobic activity utilized in these programs ranged widely, encompassing Mind–Body exercise such as yoga, Tai Chi, and Qigong [51,71,72], traditional aerobic exercise (swimming, running, cycling) [50,69,70,73], and mixed exercise [52,74,76,77]. Anerobic exercise mainly involves high-intensity interval exercise [49,75]. Exercise intensity was categorized as low, moderate, or high based on heart rate.

### 3.4. Risk of Bias and Quality Assessment

**Figure 2:**
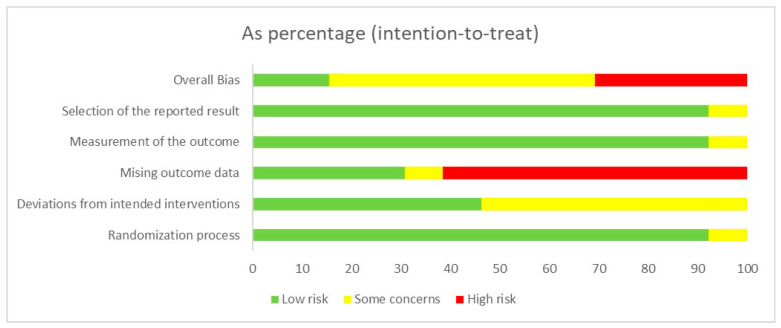
Summary of assessment of methodological quality using RoB 2.0.

To assess the risk of bias in the included randomized controlled trials, we utilized the RoB 2.0 assessment tool. The analysis was meticulously reviewed by our two researchers, with the outcomes thoroughly deliberated upon. Drawing from the Cochrane Collaboration’s criteria for determining the risk of bias outlined in the *Cochrane Handbook for Systematic Reviews of Interventions* [62], we classified the studies into distinct categories based on the level of bias, which leads to one of the following conclusions: low risk of bias, some concerns, or high risk of bias. Our analysis revealed that two studies presented a low risk of bias [49,73], three studies raised some concerns [50,69,75], and eight studies were judged to be at high risk of bias [51,52,70,71,72,74,76,77], as depicted in Figure 2 and Supplementary Table S3.

### 3.5. Primary Outcome

**Figure 3:**
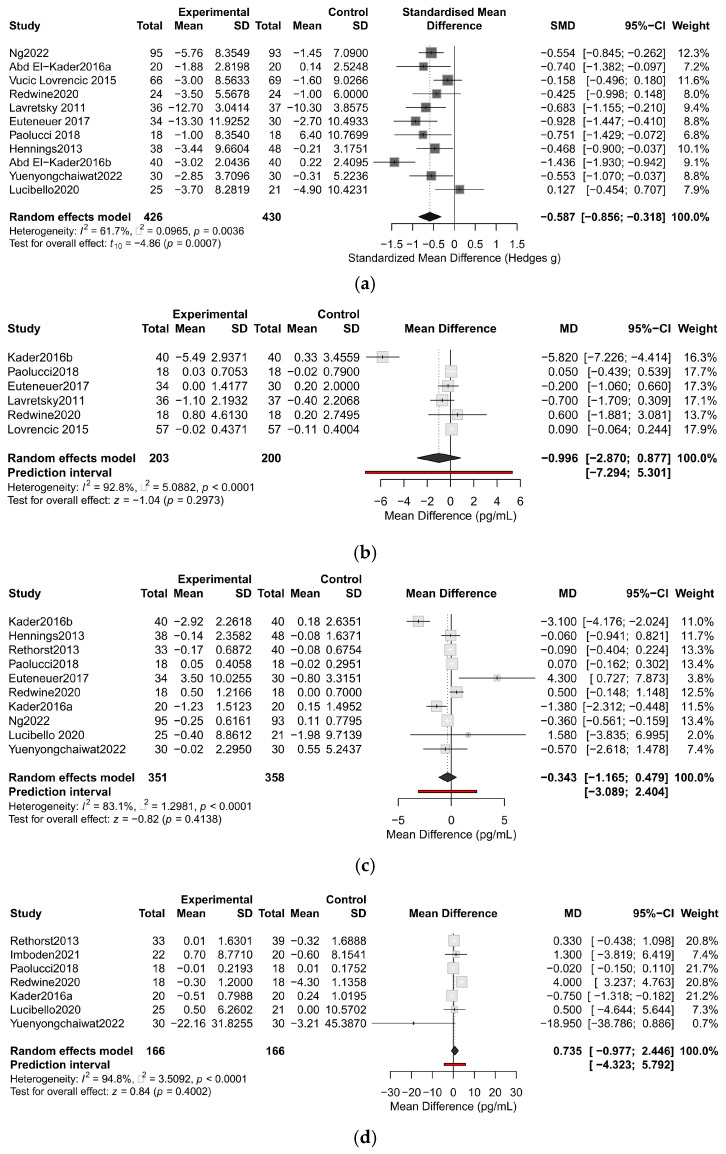
Forest plot on effect of exercise compared to control group on depressive symptoms and biomarkers in adults with depressive disorder; (**a**) effect of exercise on depressive symptoms; (**b**) effect of exercise on CRP; (**c**) effect of exercise on IL-6; (**d**) effect of exercise on TNF-α. The red horizontal line represents the prediction interval, and the black diamond indicates the pooled mean difference with its 95% confidence interval. The references in Figure 3 are Ng2022 [51], Abd El-Kader2016a [69], Vučić Lovrenčić 2015 [76], Redwine2020 [72], Lavretsky 2011 [71], Euteneuer 2017 [50], Paolucci 2018 [49], Hennings2013 [77], Abd El-Kader2016b [70], Yuenyongchaiwat2022 [74], Lucibello2020 [75], Rethorst2013 [52], Imboden2021 [73].

Among the 13 studies included in the meta-analysis, 2 studies did not provide detailed data on depression symptom scores and 11 studies suggested exercise alleviated depressive symptoms, with 8 studies showing statistical significance. Our findings demonstrated that exercise significantly improved depressive symptoms (SMD = −0.59, 95%CI: [−0.86, −0.32], I^2^ = 61.7%, *p* < 0.001) (Figure 3). The funnel plot showed approximate symmetry, and sensitivity analysis indicated that excluding any single study retained statistically significant results. When excluding Kader et al. (2016b) [70], heterogeneity decreased to 30% (SMD = −0.49, 95%CI: [−0.70, −0.28], I^2^ = 30%, *p* < 0.0001). The funnel plot and sensitivity analysis plot are shown in Supplementary Figure S1.

Among the 13 articles reviewed, three categories of peripheral biomarkers were reported, with CRP, TNF-α, and IL-6 being the most commonly reported. Meta-analysis revealed that exercise did not reduce the levels of CRP, IL-6, and TNF-α (CRP, MD = −1.00, 95%CI: [−2.87, 0.88], I^2^ = 92.8%, *p* = 0.30; TNF-α, MD = 0.74, 95%CI: [−0.98, 2.45], I^2^ = 94.8%, *p* = 0.40; IL−6, MD = −0.34, 95%CI: [−1.17, 0.48], I^2^ = 83.1%, *p* = 0.413) (Figure 3). The reported 95% prediction intervals (PIs) were exceptionally wide, encompassing both null and potential clinically meaningful effects in either direction for CRP, IL-6, and TNF-α (see Supplementary Table S5 for all intervals), indicating profound uncertainty in predicting the true effect in a new setting. The funnel plot, sensitivity analysis, and influence diagnostics analysis are shown in Supplementary Figure S2 and Tables S4 and S6.

Six trials were incorporated into the meta-analysis of CRP, with four studies suggesting that exercise might reduce CRP levels, though only one study reached statistical significance. The pooled results showed no significant association between exercise and CRP levels (MD = −1.00, 95%CI: [−2.87, 0.88], I^2^ = 92.8%, *p* = 0.30). Sensitivity analysis revealed that removing Kader2016b reduced I^2^ to 0%, resulting in MD = 0.06 (95%CI: −0.11, 0.24).

The meta-analysis of IL-6 encompassed 10 eligible studies: 7 trials suggested exercise might lower IL-6 levels, with 3 studies achieving statistical significance. Our study found no notable association between exercise and IL-6 levels (MD = −0.34, 95%CI: [−1.17, 0.48], I^2^ = 83.1%, *p* = 0.41). Sensitivity analysis confirmed robustness, as excluding any study did not alter the results.

Seven trials were incorporated into the meta-analysis of TNF-α: one study reported exercise increased TNF-α with statistical significance, while one study showed the opposite trend. The results indicated no significant association between exercise and TNF-α levels (MD = 0.74, 95%CI: [−0.98, 2.45], I^2^ = 94.8%, *p* = 0.40). For TNF-α, excluding Redwine2020 substantially reduced heterogeneity (I^2^ from 94.8% to 53.6%), resulting in MD = −0.15 (95%CI: −0.93, 0.62).

### 3.6. Subgroup Analysis

We conducted subgroup analyses based on factors including age, severity of depression, presence or absence of comorbidities, exercise type, exercise intensity, frequency, and length. Subgroup analyses revealed that the sample’s age, proportion of females, severity of depression, presence or absence of comorbidities, and exercise type, intensity and frequency demonstrated a minimal to negligible impact on the exercise-induced changes in biomarker concentrations in the bloodstream. However, durations of 8–12 weeks were associated with a significant reduction in the level of TNF-α (n = 86; MD = −0.73, 95%CI: [−1.30, −0.17], I^2^ = 0, *p* < 0.05), as shown in Table 2. The forest plot of different subgroups on CRP, IL-6, and TNF-α are presented in Supplementary Figures S3–S5.

### 3.7. Meta-Regression Analysis

To further explore the sources of heterogeneity, meta-regression analyses were conducted. Univariable analyses revealed differential response patterns of inflammatory biomarkers to exercise intervention characteristics. For CRP (k = 6) and IL-6 (k = 10), none of the analyses reached statistical significance. TNF-α (k = 7) exhibited the most distinct response pattern, with exercise length showing a significant positive correlation with TNF-α levels in a linear model (β = 0.24, *p* = 0.039), a finding contrary to clinical expectations. The prespecified unified multivariable meta-regression analyses that combined all three inflammatory biomarkers (k = 21). This model included key exercise parameters and participant characteristics as covariates. Collinearity among these covariates was assessed via variance inflation factors (VIF > 4 indicated concern). The goal of this model was to assess the independent contributions of these key covariates while adjusting for biomarker type. This model demonstrated that the overall explained variance by the included moderators remained very low (adjusted R^2^ values ranging from −0.22 to 0.20), and substantial heterogeneity persisted (I^2^ = 91.7%), indicating the presence of unmeasured moderators.

Given the apparent inconsistency of the positive linear relationship between exercise duration and TNF-α, we formally tested for non-linearity. A quadratic model provided a markedly better fit than linear specification (ΔAIC = 6.77, ΔBIC = 8.06) with significant curvature (*p* for the quadratic term = 0.003) and reduced between-study heterogeneity from I^2^ = 88.8% (linear) to I^2^ = 5.2% (quadratic). An RCS model corroborated the curvature and was comparable to the quadratic specification. The fitted curves indicate a nadir (greatest reduction in TNF-α) within the 8–12-week window, with values tending to rise again beyond 12 weeks (Figure S6). Categorical analysis (<8, 8–12, >12 weeks) was directionally consistent with this pattern. These results convert the initial counterintuitive linear finding into an analytic result: the relationship seems to be non-linear and with an apparent “8–12-week sweet spot”. Detailed data and figures are available in Supplementary Tables S4 and S5 and Figure S6.

## 4. Discussion

In our study, we identified 13 RCTs involving 1004 participants. We examined the impacts of exercise interventions with varying degrees of intensity, type, frequency, and length on the levels of peripheral proinflammatory factors and depressive symptom among patients with depression. The findings of our research showed that exercise intervention could improve depression scale scores in individuals with depression, but overall, exercise intervention did not affect the level of inflammatory factors. However, subgroup analysis showed that exercise intervention lasting 8–12 weeks decreased levels of TNF-α. Nevertheless, univariable regression analysis demonstrated a significant positive association between intervention length and TNF-α levels. This conflict suggests non-linear biological responses where 8–12 weeks represents an optimal window for TNF-α reduction, while longer durations may induce compensatory inflammatory processes.

The antidepressant effects of exercise have been confirmed, and it may even exert potential protective effects, making it a viable adjunctive treatment for depression [78,79,80]. Our findings demonstrate that exercise combined with pharmacological treatment can improve the severity of depressive symptoms in individuals with major depression. It is beyond doubt that exercise is beneficial for depressive disorder. An 11-year HUNT cohort study including 33,908 healthy adults showed that regular exercise helps to reduce the prevalence of depressive disorder and that 12% of potential depression cases could be averted if every participant engaged in at least 60 min of weekly exercise [81]. However, the mechanisms underlying exercise-induced improvement in depressive severity remain unclear. Current studies suggest that exercise may alleviate depression through increased BDNF levels in brain regions such as the hippocampus and modulating neuroimmune responses in the brain [82,83,84,85]. An animal study has shown that 4 weeks of aerobic running exercise significantly reduced the depressive-like behaviors of mice exposed to chronic unpredictable stress (CUS). This also led to a decline in microglial numbers and brought about changes in their shape within three specific hippocampal regions, effectively rebalancing the M1/M2 microglial states. These neural effects were mirrored by shifts in the production and release of both proinflammatory and anti-inflammatory cytokines in CUS-exposed mice. The underlying mechanisms may likely involve elevations in peripheral tissue such as fat stores and muscle, as well as increased plasma adiponectin. Additionally, running exercise appears to boost the expression of AdipoR1 in the hippocampus and activate the AMPK-NF-κB/STAT3 signaling pathways [86]. Furthermore, regular aerobic exercise can reprogram peripheral tryptophan metabolism by enhancing kynurenine aminotransferase (KAT) expression in skeletal muscle via the PGC-1α1 pathway. This adaptation promotes the conversion of kynurenine to the neuroprotective metabolite kynurenic acid, thereby lowering circulating kynurenine levels and limiting its entry into the brain. Through this shift, exercise helps reduce the generation of neurotoxic metabolites such as quinolinic acid, modulates immune responses, and confers neuroprotection. This mechanism mediates the potential process by which exercise improves depression [87,88,89].

Our findings revealed that exercise had no statistically significant effect on inflammatory marker levels. This finding aligns with the results obtained by previous research. One meta-analysis suggested that long-term exercise interventions do not significantly modulate baseline IL-6 and IL-1β [90]. Moreover, randomized controlled trials revealed that there was no clear dose-dependent relationship between 12-week exercise intervention and peripheral cytokine levels in patients with depression [52]. This lack of efficacy might arise from the interplay of multifactorial biological mechanisms. The effects of exercise on peripheral proinflammatory factors are influenced by several factors including exercise type (aerobic, anaerobic, mixed), intensity, volume, and subject-specific attributes (NSAID use, sex, age, diet, baseline activity, and comorbidities including obesity, diabetes, and autoimmune diseases). The variance in exercise parameters might directly shift the balance between anti-inflammatory classical IL-6Rα signaling and proinflammatory sIL-6R trans-signaling pathways [91,92,93]. Pretreatment with medications (e.g., selective serotonin reuptake inhibitors) in participants might mask any observable alterations in cytokine concentrations [52]. Moreover, during exercise, skeletal muscle mobilizes multiple energy substrates, including branched-chain amino acids (BCAAs) and tryptophan (Trp). Muscle takes up and metabolizes these amino acids through a series of transporters (e.g., the L-type amino acid transporter (LAT) family) and metabolic enzymes (e.g., BCAT, BCKDH), thereby modulating the flux of the peripheral Trp–kynurenine (Kyn) pathway. Circulating BCAAs can compete with Trp and certain Kyn metabolites via the large neutral amino acid (LNAA) transport system, consequently altering Trp/Kyn distribution within tissues and the brain. Experimental evidence indicates that BCAAs or other LNAAs can reduce kynurenic acid (KYNA) production—primarily by inhibiting Kyn uptake into tissues (transport competition), although the direct inhibition of kynurenine aminotransferase (KAT) activity has also been reported under certain in vitro conditions. Such metabolic interplay may amplify peripheral inflammatory responses through a variety of pathways [94,95,96,97,98]. Finally, the results of the studies included in this meta-analysis were all levels of peripheral inflammatory factors. There might be dissociation between central and peripheral inflammatory markers. Thus, finding a reliable and sensitive method for central inflammation detection and analysis is still a challenge.

The results of subgroup analysis suggested that 8–12 weeks of exercise intervention is associated with a reduction in TNF-α levels in patients with depression. However, the results of univariable regression indicated that studies with longer exercise lengths showed a higher level of TNF-α. Synthesizing the above outcomes, the peak of exercise-induced improvement of TNF-α levels appeared around 8–12 weeks in patients with depression. The temporal dimension of intervention efficacy on TNF-α exhibits non-linear characteristics: short-term programs (median 9 weeks, range 4–15 weeks) are not sufficient to initiate anti-inflammatory adaptations [90], whereas excessive exercise load or exercise dosage can lead to an increase in chronic inflammation [90,99]. If the exercise load and dosage are too high, the body will be in a state of chronic low-concentration inflammation for a long time, and its ability to inhibit the production of TNF-α will be weakened, which may be one of the reasons for the increase in TNF-α levels. This biphasic response pattern substantiates prior observations by Rose et al., who identified an 8-week minimal threshold (optimal 9–12 weeks) to elicit stable inflammatory marker adaptations, suggesting the existence of critical temporal windows for exercise-induced immunomodulation in depressive populations [80,99].

There is evidence that IL-6 exerts a dual regulatory role on TNF-α through dichotomous signaling by promoting TNF-α release in proinflammatory states, yet suppressing it via pathways such as TLR/NF-κB inhibition in response to exercise [100,101]. Exercise-induced IL-6 release is closely linked to the energy demand and metabolism of skeletal muscle [102,103]. There might be a “U”-shaped relationship between training load and basal IL-6 levels, with non-excessive training levels being associated with lower IL-6 levels in either sedentary or excessively exercising individuals [45,104]. If the exercise volume is too low (e.g., walking), it cannot induce an IL-6 anti-inflammatory effect [105]. On the other hand, if the exercise volume is too high, it might hinder or restrict the secretion of anti-inflammatory cytokines, which are needed to decrease the enhancement in TNF-α levels [106]. Therefore, a suitable exercise volume can beneficially skew the function of IL-6’s anti-inflammatory effect, which contributed to the suppression of TNF-α. For the above reasons, there might be an appropriate exercise volume (8–12 week) for the improvement of enhanced levels of TNF-α in patients with depression.

There are substantial discrepancies between our meta-regression results and subgroup analysis findings. Specifically, in univariate regression analysis, intervention length was significantly positively correlated with TNF-α levels, which is contrary to some previous evidence, while subgroup analysis suggested a potential non-linear or threshold effect. These divergences may arise from methodological differences between the two approaches: subgroup analysis examines discrete categories and emphasizes between-group differences, whereas standard meta-regression models continuous variables under a linearity assumption. Notably, quadratic and restricted cubic spline terms revealed a significant non-linear relationship that neither initial method fully captured. This curve, characterized by a reduction in TNF-α up to 8–12 weeks followed by a gradual increase, helps reconcile the contradictions and highlights the limitation of applying linear models to inherently non-linear biological responses. Furthermore, other significant subgroup findings were not retained in multivariable meta-regression. This may be attributed to several factors: reduced statistical power in smaller subgroups; confounding by unmeasured variables such as exercise intensity or type, or timing of biomarker measurement (acute or chronic phase); and multicollinearity among moderators, which can inflate variance and obscure true effects. Importantly, the low explanatory power (adjusted R^2^ ranging from −0.22 to 0.20) and persistently high heterogeneity (I^2^ > 90%) in multivariable models underscore that the included covariates explain little of the between-study variance, indicating that critical effect modifiers remain unaccounted for. Therefore, the discrepancies do not invalidate either set of results but rather emphasize the complexity of inflammatory responses to exercise. They underscore the necessity of using complementary analytical approaches and prespecified non-linear models to avoid misleading interpretations from oversimplified linear assumptions.

This meta-analysis offers several advantages over prior work: First, we have comprehensively revised and synthesized the outcomes of various exercise programs (type, intensity, length, and frequency) on peripheral proinflammatory factor levels in all adult populations with depression [90]. Second, we not only included patients with depression diagnosed according to the DSM-V criteria but also incorporated those diagnosed with depression by local hospitals based on clinical diagnostic criteria. The advantage of this strategy lies in the fact that it expands the scope of the sample sources, making the research results more in line with the real clinical environment. It reflects the manifestations of depression under different diagnostic practices, thus enhancing the generalizability of the conclusions. Additionally, it strengthens the robustness of the research findings and reduces issues such as cultural biases and insufficient applicability [107]. There are some limitations of the included studies: First, lots of studies included in this meta-analysis focused on low-to-moderate intensity exercise, with fewer studies focused on high-intensity exercise; furthermore, systematic exercise progression was scarcely reported (only one study described it in detail), limiting meaningful analysis of dose–response relationships. Second, because of the small number of included RCTs, the data of this meta-analysis were collected from different sexes and a wide range of age parameters. However, these inherent individual variabilities had impacts on the results. The number of subjects in our research is limited, and subsequent large-scale clinical studies are still needed. Third, this study has several limitations. Although most of the included studies explicitly distinguished between populations with and without comorbidities, the levels of inflammatory markers may still be influenced by various uncontrolled factors, such as obesity, dietary habits, acute or transient illnesses (e.g., common cold), and extreme geographical environments (e.g., Antarctica, isolated islands, or the Arctic). Therefore, potential confounding effects cannot be fully excluded, which may, to some extent, affect the interpretation and generalizability of the findings. Fourth, some included studies combined exercise interventions with additional activities (e.g., pharmacological treatment not explicitly excluded, glucose control, or psychoeducation). Because of the limited number of studies, we were unable to completely exclude these combined interventions. This may have introduced potential confounding effects. Finally, most of the included studies did not provide detailed information on participants’ dietary patterns, traditional medicine, and nutraceutical products, which may influence inflammatory markers, and thus we could not control for this factor. There are also some limitations of this review: First, despite using rigorous heterogeneity analyses (leave-one-out, Hartung–Knapp adjustments), between-study heterogeneity remained very high, suggesting that unmeasured factors such as diet, traditional medicine or nutraceutical products use, or environment may influence biomarker levels. Second, while non-linear modeling (quadratic and restricted cubic splines) provided stronger evidence for a potential U-shaped biomarker response, the limited number, the extreme heterogeneity, and the model-dependent nature of these biomarker findings reduce the stability of this finding. Third, comorbidities and other confounders were insufficiently reported in many studies, limiting the ability to isolate exercise-specific effects.

## 5. Conclusions

Our meta-analysis supports that exercise exerts a positive influence on depressive symptoms, whereas its effects on peripheral inflammatory markers remain uncertain due to high heterogeneity and potential unmeasured moderators. Subgroup and non-linear analyses suggest that interventions of 8–12 weeks may be linked to reductions in TNF-α, but this finding should be interpreted cautiously due to the limited and uneven distribution of the studies. Although exercise seems to confer mood-boosting benefits, further rigorous research is necessary to determine whether its antidepressant properties are tied to inflammation. Future studies should employ more robust methodologies, extend trial length, account for participants’ medication use, and include proper control groups that do not involve exercise. Furthermore, the detection of integrating CNS biomarkers will be essential to explore the peripheral/central connection. Only through such comprehensive investigations can we truly establish if exercise’s mental health benefits operate through inflammatory pathways.

## Figures and Tables

**Table 1:** Summary of study and participant characteristics of included studies.

Author/Year/Country	Sample Size (n)		Mean Age (Years)	Intervention				Control Group Treatment	Biomarkers	Depressive Symptom Measure	Depression Severity
	Exercise	Control		Type of intervention	Intensity	Sessions per wk	Wks				
Euteneuer 2017 [50] Germany	34	30	37.3 ± 12.2	Mixed exercise	Low–moderate	4	16	Wait-list	CRP/IL-6/IL-10	BDI-Ⅱ	MDD
Lavretsky 2011 [71] America	36	37	70 ± 7.2	Mind–Body exercises	Low–moderate	1	10	Health education	CRP	HAMD/HAMA/ PSQI	MDD
Abd El-Kader & Al-Jiffri, 2016b [70] America	40	40	34.17 ± 4.98	Traditional aerobic exercises	Middle–high	3	12	Wait-list	IL-4/IL/6/CRP/TNF-β	BDI	Mild
Ng 2022 [51] Hong Kong	95	93	55.49 ± 10.12	Mind–Body exercises	Low–moderate	3	12	Wait-list	IL-6/IL-1β	PSQI/CES-D	Moderate–Severe
Paolucci 2018 [49] Canda	19	18	21 ± 2	HIIT	High–moderate	3	6	Wait-list	IL-6/TNF-α/CRP	BDI-Ⅱ/PSS/BAI	Moderate
Redwine 2020 [72] America	24	24	65 ± 8.31	Mind–Body Exercises	Moderate	2	16	Usual care (standard medical treatment)	IL-6/TNF-α/CRP	BDI	Mild
Vučić Lovrenčić 2015 [76] Croatia	66	69	58.15 ± 5.53	Mixed exercise	Low–moderate	6	48	Enhanced treatment	CRP	CES-D	Moderate–Severe
Abd El-Kader & Al-Jiffri, 2016a [69] America	20	20	69.035 ± 5.96	Traditional aerobic exercises	Moderate	3	8	Wait-list	TNF-α/ IL-6	BDI system/ POMS/ RSES/ SF-36	Mild
Hennings 2013 [77] Germany	38	48	34.51 ± 12.84	Active week x passive week (cross-over trial)	Moderate	7	4	Active week x passive week (cross-over trial)	IL-6	BDI	MDD
Rethorst 2013 [52] America	53	52	47.51 ± 9.44	Mixed exercise	Moderate	16KKW per week	12	4KKW for 12 weeks	IL-6/TNF-α	HRSD/IDS-C/IDS-SR	MDD
Imboden 2021 [73] Switzerland	22	20	39.9 ± 11.4	Traditional aerobic exercises	Moderate	3	6	Stretching condition (active control)	TNF-α	HDRS17	Moderate
Yuenyongchaiwat 2023 [74] Thailand	30	30	71.7 ± 4.9	Mixed exercise	Low	5	12	Usual care (live normally without intervention)	IL-6/TNF-α	TGDS	Mild
Lucibello 2020 [75] Canada	25	21	19.8 ± 2.2	HIIT	High	3	9	Placebo	IL-6/TNF-α	BDI-II	Mild–Moderate

Note. BDI = Beck Depression Inventory. BDI-Ⅱ = Beck Depression Inventory—Second Edition. HAMD/HRSD = Hamilton Depression Rating Score. HAMA = Hamilton Anxiety Rating Scale. PSQI = Pittsburgh Sleep Quality Index. CES-D = Center for Epidemiologic Studies Depression Scale. POMS = Profile of Mood States. RSES = Rosenberg Self-Esteem Scale. SF-36 = Medical Outcomes Study 36-Item Short-Form Health Survey HDRS17 = Hamilton Depression Rating Scale—17 items. IDS-C = Inventory of Depressive Symptomatology—Clinician Rated. IDS-SR = Inventory of Depressive Symptomatology—Self Report. TGDS = Thai Geriatric Depression Scale. MDD = major depressive disorder. HIIT = high-intensity interval training. CRP = C-reactive protein. Il-6 = interleukin-6. TNF-α = tumor necrosis factor-alpha. Traditional Aerobic Exercises = running, swimming, cycling, and so on. Mind–Body Exercises = Yoga, Qigong, and Tai Chi.

**Table 2:** Effect of exercise training on biomarkers in adults with depressive disorder.

	CRP			IL-6			TNF-α		
	Interventions	Participants	Effect Metrics	Interventions	Participants	Effect Metrics	Interventions	Participants	Effect Metrics
Type of training									
Aerobic	3	189	−2.04 [−5.88,1.80], I^2^ = 94.9%, *p* = 0.30	4	344	−1.03 [−2.51,0.45], I^2^ = 91.7%, *p* = 0.17	3	118	1.55 [−1.73, 4.83], I^2^ = 97.9%, *p* = 0.35
HIIT	1	36	0.05 [−0.44,0.54], NA, *p* = 0.84	2	82	0.07 [−0.16,0.30], I^2^ = 0, *p* = 0.54	2	82	−0.02 [−0.15, 0.11], I^2^ = 0, *p* = 0.77
Mixed	2	178	0.08 [−0.07,0.23], I^2^ = 0, *p* = 0.30	4	283	−0.07 [−0.36,0.23], I^2^ = 49.9%, *p* = 0.65	2	132	−6.66 [−24.82, 11.51], I^2^ = 72.4%, *p* = 0.47
Intensity									
Low	2	178	0.08 [−0.07, 0.23], I^2^ = 0, *p* = 0.30	4	385	−0.23 [−0.50, 0.04], I^2^ = 64.3%, *p* = 0.09	2	132	−6.66 [−24.82, 11.51], I^2^ = 72.4%, *p* = 0.47
Moderate	2	109	−0.52 [−1.45, 0.42], I^2^ = 0, *p* = 0.28	3	162	−0.28 [−1.36, 0.81], I^2^ = 81%, *p* = 0.618	3	118	1.55 [−1.73, 4.83], I^2^ = 97.9%, *p* = 0.354
High	2	116	−2.85 [−8.60, 2.91], I^2^ = 98.3%, *p* = 0.33	3	162	−0.99 [−3.63, 1.64], I^2^ = 93.8%, *p* = 0.50	2	82	−0.02 [−0.15, 0.11], I^2^ = 0, *p* = 0.77
Frequency									
≤2/week	2	109	−0.52 [−1.45, 0.42], I^2^ = 0, *p* = 0.28	1	36	0.50 [−0.15,1.15], NA, *p* = 0.17	1	36	4.00 [3.24, 4.76], NA, *p* = 0.60
3–4/week	3	180	−1.94 [−5.66, 1.77], I^2^ = 96.7%, *p* = 0.31	6	454	−0.36 [−2.08,1.35], I^2^ = 89.5%, *p* = 0.68	4	164	−0.29 [−0.94,0.37], I^2^ = 52.8%, *p* < 0.001
≥5/week	1	114	0.09 [−0.06,0.24], NA, *p* = 0.25	2	146	−0.14 [−0.95,0.67], I^2^ = 0, *p* = 0.74	1	60	−18.95 [38.79, 0.89], NA, *p* = 0.06
Length (weeks)									
<8	1	36	0.05 [−0.44,0.54], NA, *p* = 0.84	2	122	0.06 [−0.16,0.29], I^2^ = 0, *p* = 0.59	2	78	−0.02 [−0.15,0.11], I^2^ = 0, *p* = 0.72
8–12	1	73	−0.70 [−1.71,0.31]. NA, *p* = 0.17	2	86	−1.15 [−2.70,0.40], I^2^ = 10.3%, *p* = 0.15	2	86	**−0.73 [−1.30, −0.17], I^2^ = 0, *p* = 0.01**
≥12	4	294	−1.35 [−4.29,1.58], I^2^ = 95.6%, *p* = 0.37	6	501	−0.24 [−1.70,1.22], I^2^ = 87.6%, *p* = 0.75	3	168	1.20 [−3.26,5.66], I^2^ = 95.9%, *p* = 0.60
Age (years)									
<30	1	36	0.05 [−0.44,0.54], NA, *p* = 0.84	2	82	0.07 [−0.16,0.30], I^2^ = 0, *p* = 0.538	2	82	−0.02 [−0.15,0.11], I^2^ = 0, *p* = 0.77
30–59	3	258	−1.93 [−5.65,1.80], I^2^ = 97%, *p* = 0.31	5	491	−0.26 [−2.04,1.53], I^2^ = 88.4%, *p* = 0.78	3	114	0.35 [−0.41, 1.11], I^2^ = 0, *p* = 0.37
≥60	2	109	−0.52 [−1.45,0.42], I^2^ = 0, *p* = 0.28	3	136	−0.43 [−1.71, 0.85], I^2^ = 81.3%, *p* = 0.513	3	136	−0.19 [−6.73, 6.36], I^2^ = 98%, *p* = 0.96
Depression severity									
Mild	2	116	−2.70 [−8.98, 3.60], I^2^ = 94.9%, *p* = 0.40	4	216	−1.14 [−2.72, 0.44], I^2^ = 91.3%, *p* = 0.16	3	136	−0.19 [−6.73, 6.36], I^2^ = 98%, *p* = 0.96
Moderate	2	150	0.09 [−0.06; 0.23], I^2^ = 0, *p* = 0.25	3	270	−0.14 [−0.56, 0.28], I^2^ = 74.8%, *p* = 0.52	3	124	−0.02 [−0.15, 0.11], I^2^ = 0, *p* = 0.78
Severe	2	137	−0.41 [−1.07; 0.24], I^2^ = 0, *p* = 0.22	3	223	−0.06 [−0.35, 0.24], I^2^ = 65.3%, *p* = 0.71	1	72	0.33 [−0.44,1.10], NA, *p* = 0.40
Sex, % female									
>50%	3	223	0.070 [−0.08, 0.22] I^2^ = 13.3%, *p* = 0.34	6	489	−0.13 [−0.37, 0.11] I^2^ = 40.3%, *p* = 0.28	4	214	−0.01 [−0.14, 0.12] I^2^ = 30.5%, *p* = 0.87
≤50%	3	180	−1.86 [−5.83, 2.11] I^2^ = 95.8%, *p* = 0.36	4	220	−0.25 [−2.99, 2.49] I^2^ = 92.8%, *p* = 0.86	3	118	1.55 [−1.73, 4.83] I^2^ = 97.9%, *p* = 0.35
Comorbidities									
Yes	3	230	−1.75 [−5.79, 2.30] I^2^ = 97%, *p* = 0.40	5	404	−0.956 [−2.19, 0.28] I^2^ = 88.9%, *p* = 0.13	3	136	−0.19 [−6.73, 6.36] I^2^ = 98%, *p* = 0.96
No	3	173	−0.115 [−0.51, 0.28] I^2^ = 0, *p* = 0.57	5	305	0.02 [−0.16, 0.21] I^2^ = 38.6%, *p* = 0.80	4	196	−0.01 [−0.14, 0.12] I^2^ = 0, *p* = 0.89

Note: NA, not available. Bold values indicate *p* < 0.05 in subgroup analysis.

## Data Availability

The extracted data and analysis code that support the findings of this study are available from the corresponding author upon reasonable request.

## Supplementary Materials

The following supporting information can be downloaded at: https://www.mdpi.com/article/10.3390/life15091452/s1. Figure S1. Publication bias and sensitivity analysis of exercise effects on depressive symptoms. (a) Funnel plot for visual inspection of publication bias in studies assessing effect of exercise on depressive symptom; (b) leave-one-out analysis of studies assessing effect of exercise on depressive symptom, using random effects model. Figure S2: Publication bias and sensitivity analyses of exercise effects on inflammatory biomarkers. (a) Leave-one-out analysis of studies assessing effect of exercise on level of CRP, using random effects model; (b) funnel plot for visual inspection of publication bias in studies assessing effect of exercise on level of IL-6; (c) leave-one-out analysis of studies assessing effect of exercise on level of IL-6, using random effects model; (d) leave-one-out analysis of studies assessing effect of exercise on level of TNF-α, using random effects model. Figure S3: Subgroup analysis of exercise-induced CRP level changes by intervention parameters and participant characteristics. (a) Forest plot of mean difference in CRP levels at different exercise intensities; (b) forest plot of mean difference in CRP levels at different exercise lengths; (c) forest plot of mean difference in CRP levels at different exercise frequencies; (d) forest plot of mean difference in CRP levels at different depression severities; (e) forest plot of mean difference in CRP levels at different exercise types; (f) forest plot of mean difference in CRP levels at different age ranges. Figure S4: Subgroup analysis of exercise-induced IL-6 level changes by intervention parameters and participant characteristics. (a) Forest plot of mean difference in IL-6 levels at different exercise intensities; (b) forest plot of mean difference in IL-6 levels at different exercise lengths; (c) forest plot of mean difference in IL-6 levels at different exercise frequencies; (d) forest plot of mean difference in IL-6 levels at different depression severities; (e) forest plot of mean difference in IL-6 levels at different exercise types; (f) forest plot of mean difference in IL-6 levels at different age ranges. Figure S5: Subgroup analysis of exercise-induced TNF-α level changes by intervention parameters and participant characteristics. (a) Forest plot of mean difference in TNF-α levels at different exercise intensities; (b) forest plot of mean difference in TNF-α levels at different exercise lengths; (c) forest plot of mean difference in TNF-α levels at different exercise frequencies; (d) forest plot of mean difference in TNF-α levels at different depression severities; (e) forest plot of mean difference in TNF-α levels at different exercise types; (f) forest plot of mean difference in TNF-α levels at different age ranges. Figure S6. Non-linear relationship between exercise duration and change in TNF-α levels. Table S1: Specific search strategy for each database. Table S2. Characteristics of intervention implementation in included studies. Table S3. Risk of bias assessment for RCTs based on the ROB2 tool. Table S4. Influence diagnostics for pooled mean differences. Table S5. Main meta-analysis results with 95% confidence and prediction intervals. Table S6. Sensitivity analyses: changes in pooled estimates and heterogeneity upon removal of influential studies. Table S7. Univariable meta-regression on association between exercise training and biomarkers in adults with major depressive disorder. Table S8. Multivariable meta-regression on association between exercise training and biomarkers in adults with major depressive disorder. Table S9: Supplementary study-level data and intervention characteristics; Supplementary File S2: PRISMA_2020_checklist.

## Abbreviations

The following abbreviations are used in this manuscript:

AMPK	Adenosine Monophosphate-Activated Protein Kinase
BCAT	Branched-Chain Amino Acid Transaminase
BCKDH	Branched-Chain α-Keto Acid Dehydrogenase Complex
BDNF	Brain-Derived Neurotrophic Factor
CCL3	C-C Motif Chemokine Ligand 3
CI	Confidence Interval
CRP	C-Reactive Protein
HPA axis	Hypothalamic–Pituitary–Adrenal Axis
ICAM-1	Intercellular Adhesion Molecule 1
IL-2	Interleukin-2
IL-6	Interleukin-6
NF-κB	Nuclear Factor Kappa-Light-Chain-Enhancer of Activated B Cells
STAT3	Signal Transducer and Activator of Transcription 3
TLR	Toll-Like Receptor
TNF-α	Tumor Necrosis Factor alpha
TNF-β	Tumor Necrosis Factor beta (Lymphotoxin-α)
