# Supplementary material for: Effect of Different Exercise Modalities on Inflammatory Markers in Individuals with Depressive Disorder: A Systematic Review and Meta-Analysis
Source: Life (Basel). 2025 Sep 16;15(9):1452. doi: 10.3390/life15091452 (PMC12472113; doi:10.3390/life15091452)
Supplement: Supplementary file 1 [file life-15-01452-s001.zip › Supplementary File S1 (Figures and Tables).pdf]

Table S1. Specific Search Strategy for Each Database.

Database	Search Strategy
PubMed, The Cochrane Library	#1 (((((((((((((depressive disorders[MeSH Terms]) OR (Disorder, Depressive[Title/Abstract])) OR (Depressive Neuroses[Title/Abstract])) OR (Depressive Neurosis[Title/Abstract])) OR (Endogenous Depression[Title/Abstract])) OR (Endogenous Depressions[Title/Abstract])) OR (Depressive Syndrome[Title/Abstract])) OR (Depressive Syndromes[Title/Abstract])) OR (Neurotic Depression[Title/Abstract])) OR (Neurotic Depressions[Title/Abstract])) OR (Melancholia[Title/Abstract])) OR (Melancholias[Title/Abstract])) OR (Unipolar Depression[Title/Abstract])) OR (Unipolar Depressions[Title/Abstract])<br>#2 ((((((((((((((exercise[MeSH Terms])) OR (Exercises[Title/Abstract])) OR (Physical Activity[Title/Abstract])) OR (Physical Activities[Title/Abstract])) OR (Physical Exercise[Title/Abstract])) OR (Physical Exercises[Title/Abstract])) OR (Acute Exercise[Title/Abstract])) OR (Acute Exercises[Title/Abstract])) OR (Isometric Exercises[Title/Abstract])) OR (Isometric Exercise[Title/Abstract])) OR (Aerobic Exercise[Title/Abstract])) OR (Aerobic Exercises[Title/Abstract])) OR (Exercise Training[Title/Abstract])) OR (Exercise Trainings[Title/Abstract])<br>#3 (((inflammation[MeSH Terms]) OR (Inflammations[Title/Abstract]))) OR (Innate Inflammatory Response[Title/Abstract])) OR (Innate Inflammatory Responses[Title/Abstract])

	#1 AND #2 AND #3
Web of Science	#1 TS = (depressive disorders OR Disorder, Depressive OR Depressive Neuroses OR Depressive Neurosis OR Endogenous Depression OR Endogenous Depressions OR Depressive Syndrome OR Depressive Syndromes OR Neurotic Depression OR Neurotic Depressions OR Melancholia OR Melancholias OR Unipolar Depression OR Unipolar Depressions)<br>#2 TS = (exercise OR Exercises OR Physical Activity OR Physical Activities OR Physical Exercise OR Physical Exercises OR Acute Exercise OR Acute Exercises OR Isometric Exercises OR Isometric Exercise OR Aerobic Exercise OR Aerobic Exercises OR Exercise Training OR Exercise Trainings)<br>#3 TS = (inflammation OR Inflammations OR Innate Inflammatory Response OR Innate Inflammatory Responses)<br>#1 AND #2 AND #3
Embase	#1 'depression'/exp OR 'depression' OR 'central depression'/exp OR 'central depression' OR 'clinical depression'/exp OR 'clinical depression' OR 'depressive disease'/exp OR 'depressive disease' OR 'depressive disorder'/exp OR 'depressive disorder' OR 'depressive episode'/exp OR 'depressive episode' OR 'depressive illness'/exp OR 'depressive illness' OR 'depressive personality disorder'/exp OR 'depressive personality disorder' OR 'depressive state'/exp OR 'depressive state' OR 'depressive symptom'/exp OR 'depressive symptom' OR 'depressive syndrome'/exp OR 'depressive syndrome' OR 'depressivity'/exp OR 'depressivity' OR 'mental depression'/exp OR 'mental depression' OR 'parental depression'/exp OR 'parental depression'<br>#2 'exercise'/exp OR 'exercise' OR 'biometric exercise'/exp OR

	'biometric exercise' OR 'effort'/exp OR 'effort' OR 'exercise capacity'/exp OR 'exercise capacity' OR 'exercise performance'/exp OR 'exercise performance' OR 'exercise training'/exp OR 'exercise training' OR 'exertion'/exp OR 'exertion' OR 'fitness training'/exp OR 'fitness training' OR 'fitness workout'/exp OR 'fitness workout' OR 'physical conditioning, human'/exp OR 'physical conditioning, human' OR 'physical effort'/exp OR 'physical effort' OR 'physical exercise'/exp OR 'physical exercise' OR 'physical exertion'/exp OR 'physical exertion' OR 'physical work-out'/exp OR 'physical work-out' OR 'physical workout'/exp OR 'physical workout'<br>#3 'inflammation'/exp OR 'inflammation' OR 'acute inflammation'/exp OR 'acute inflammation' OR 'inflammation reaction'/exp OR 'inflammation reaction' OR 'inflammation response'/exp OR 'inflammation response' OR 'inflammatory condition'/exp OR 'inflammatory condition' OR 'inflammatory lesion'/exp OR 'inflammatory lesion' OR 'inflammatory process'/exp OR 'inflammatory process' OR 'inflammatory reaction'/exp OR 'inflammatory reaction' OR 'inflammatory response'/exp OR 'inflammatory response' OR 'inflammatory syndrome'/exp OR 'inflammatory syndrome' OR 'reaction, inflammation'/exp OR 'reaction, inflammation' OR 'response, inflammatory'/exp OR 'response, inflammatory'<br>#1 AND #2 AND #3
EBSCO	#1 depressive disorder or depressive symptoms or major depressive disorder<br>#2 exercise or physical fitness or physical activity or exercise therapy or physical therapy or walking<br>#3 inflammation or inflammatory or inflammation response

#1 AND #2 AND #3

Table S2. Characteristics of Intervention Implementation in Included Studies

Study	Group exercise	Setting	Place of residence	Supervised	Type of supervision
Euteneuer 2017[50]Germany	Unclear	Unclear	Unclear	Supervised	Trained staff/research personnel
Lavretsky 2011[71]America	Yes	Hybrid (community + outpatient)	Unclear	Supervised	Certified professional Tai Chi instructors
Abd El-Kader & Al-Jiffri, 2016b[70]America	Unclear	Hospital-based pulmonary rehabilitation center	Community-dwelling	Supervised	Pulmonary rehabilitation therapist and physical therapist
Ng 2022[51] Hong Kong	Yes	University research/health center	Community-dwelling	Supervised	Certified Yoga/MBSR instructors
Paolucci 2018[49]Canda	Unclear	Laboratory	Unclear	Supervised	Trained lab staff and researchers
Redwine 2020[72]America	Unclear	Home-based	Community-dwelling	Supervised	Exercise rehabilitation specialists/research staff +remote

					supervision
Vučić Lovrenčić 2015[76]Croatia	Unclear	Outpatient/ community health center	Community-dwelling	Supervised	Qualified exercise instructors or rehabilitation therapists
Abd El-Kader & Al-Jiffri, 2016a[69] America	Unclear	Hospital/ rehabilitation laboratory	Community-dwelling	Supervised	Physical therapist
Hennings 2013[77]Germany	Unclear	Outpatient	Community-dwelling	Supervised	Trained professionals
Rethorst 2013[52] America	Unclear	Hybrid (laboratory/exercise center + home-based)	Unclear	Partially supervised	Exercise research staff through in-person and remote monitoring
Imboden 2021[73]Switzerland	Yes	Inpatient	Inpatient	Supervised	Physical or rehabilitation therapists
Yuenyongchaiwat	Yes	Hybrid (Community	Community-dwelling	Partially supervised	Exercise rehabilitation

2023[74]Thailand		center + home-based)			specialists + remote telephone coaching
Lucibello 2020[75]Canada	No	Laboratory/university exercise facility	Community-dwelling	Supervised	Trained research assistants or exercise specialists

Table S3. Risk of Bias Assessment for RCTs Based on the ROB2 Tool.

Study ID	D1	D2	D3	D4	D5	Overall
Paolucci et al., 2018	+	+	+	+	+	+
Ng et al., 2022	+	+	-	!	+	!
Abd El-Kader & Al-Jiffri, 2016b	+	!	-	+	+	-
Abd El-Kader & Al-Jiffri, 2016a	+	!	+	+	+	!
Euteneuer et al., 2017	!	!	+	+	+	!
Lavretsky et al., 2011	+	+	-	+	+	!
Redwine et al., 2020	+	+	-	+	+	!
Vučić Lovrenčić et al., 2015	+	+	-	+	+	-
Hennings et al., 2013	+	!	-	+	+	-
Imboden et al., 2021	+	+	+	+	+	+
Rethorst et al., 2013a	+	!	-	+	+	!
Yuenyongchaiwat et al., 2023	+	!	-	+	!	-
Lucibello et al., 2020	+	!	!	+	+	!

+ Low risk

! Some concerns

- High risk

D1 Randomisation process

D2 Deviations from the intended interventions

D3 Missing outcome data

D4 Measurement of the outcome

D5 Selection of the reported result

Note: The references in Table S4 are Ng2022 [51], Abd El-Kader2016a[69], Vučić Lovrenčić 2015[76], Redwine2020[72], Lavretsky 2011[71], Euteneuer 2017[50], Paolucci 2018[49], Hennings2013[77], Abd El-Kader2016b[70], Yuenyongchaiwat2022[74], Lucibello2020[75], Rethorst2013[52], Imbo-den2021[73].

Table S4 Influence Diagnostics for Pooled Mean Differences.

outcome	study	k	yi	vi	hat	z_resid	cooks_D	hat_threshold	cookD_threshold
CRP	Kader2016b	6	-5.82	0.514	0.163	-9.663	1.204	0.333	0.8
CRP	Paolucci2018	6	0.05	0.062	0.177	0.458	0.054	0.333	0.8
CRP	Euteneuer2017	6	-0.2	0.192	0.173	0.34	0.03	0.333	0.8
CRP	Lavretsky2011	6	-0.7	0.265	0.171	0.125	0.004	0.333	0.8
CRP	Redwine2020	6	0.6	1.602	0.137	0.622	0.072	0.333	0.8
CRP	Lovrencic 2015	6	0.09	0.006	0.179	0.479	0.06	0.333	0.8
IL-6	Kader2016b	10	-3.1	0.301	0.11	-3.456	0.235	0.2	0.444
IL-6	Hennings2013	10	-0.06	0.202	0.117	0.185	0.001	0.2	0.444
IL-6	Rethorst2013	10	-0.09	0.026	0.133	0.17	0	0.2	0.444
IL-6	Paolucci2018	10	0.07	0.014	0.134	0.296	0.006	0.2	0.444
IL-6	Euteneuer2017	10	4.3	3.323	0.038	2.297	0.143	0.2	0.444
IL-6	Redwine2020	10	0.5	0.109	0.125	0.64	0.056	0.2	0.444
IL-6	Kader2016a	10	-1.38	0.226	0.115	-0.776	0.096	0.2	0.444
IL-6	Ng2022	10	-0.36	0.011	0.134	-0.034	0.005	0.2	0.444

IL-6	Lucibello 2020	10	1.58	7.634	0.02	0.559	0.006	0.2	0.444
IL-6	Yuenyongchaiwat2022	10	-0.57	1.092	0.074	-0.139	0.006	0.2	0.444
TNF-α	Rethorst2013	7	0.33	0.154	0.208	-0.169	0.004	0.286	0.667
TNF-α	Imboden2021	7	1.3	6.821	0.074	0.155	0.004	0.286	0.667
TNF-α	Paolucci2018	7	-0.02	0.004	0.217	-0.344	0.031	0.286	0.667
TNF-α	Redwine2020	7	4	0.152	0.208	6.027	0.832	0.286	0.667
TNF-α	Kader2016a	7	-0.75	0.084	0.212	-0.76	0.166	0.286	0.667
TNF-α	Lucibello2020	7	0.5	6.888	0.073	-0.059	0	0.286	0.667
TNF-α	Yuenyongchaiwat2022	7	-18.95	102.428	0.007	-2.193	0.021	0.286	0.667

Note: The references in Table S4 are Ng2022 [51], Abd El-Kader2016a[69], Vučić Lovrenčić 2015[76], Redwine2020[72], Lavretsky 2011[71], Euteneuer 2017[50], Paolucci 2018[49], Hennings2013[77], Abd El-Kader2016b[70], Yuenyongchaiwat2022[74], Lucibello2020[75], Rethorst2013[52], Imbo-den2021[73].

Table S5. Main Meta-Analysis Results with 95% Confidence and Prediction Intervals.

Outcome	k	τ² (REML)	I² (%)	Pooled MD (REML)	SE (θ̂_RE),	df (HK)	95% CI L (HKSJ)	95% CI U (HKSJ)	95% PI L (t_HK)	95% PI U (t_HK)
CRP	6	5.088	92.81	-0.996	0.967	5	-3.481	1.489	-7.305	5.312
IL-6	10	1.298	83.13	-0.343	0.47	9	-1.405	0.72	-3.131	2.445
TNF-α	7	3.509	94.82	0.735	0.971	6	-1.643	3.112	-4.429	5.898

Table S6. Sensitivity Analyses: Changes in Pooled Estimates and Heterogeneity Upon Removal of Influential Studies.

Outcome	k	τ² (REML)	I² (%)	Pooled MD (REML)	SE(θ̂_RE)	df (HK)	95% CI L (HKSJ)	95% CI U (HKSJ)	95% PI L (t_HK)	95% PI U (t_HK)	Flagged studies excluded
CRP (influence-robust)	5	0	0	0.064	0.062	4	-0.107	0.236	-0.107	0.236	Kader2016b
IL-6 (influence-robust)	8	0.108	63.02	-0.151	0.177	7	-0.569	0.266	-1.034	0.732	Euteneuer2017, Kader2016b
TNF-α (influence-robust)	5	0.164	45.15	-0.138	0.222	4	-0.753	0.478	-1.419	1.143	Redwine 2020, Yuenyongchaiwat 2022

Table S7. Univariable Meta-Regression on the Association between Exercise Training and Biomarkers in Adults with Major Depressive Disorder.

	CRP						IL-6						TNF-α					
	k	Coefficient(95%CI)	I^2 (%)	*p*_value	tau^2	Adijusted_R^2	k	Coefficient(95%CI)	I^2 (%)	*p*_value	tau^2	Adijusted_R^2	k	Coefficient(95%CI)	I^2 (%)	*p*_value	tau^2	Adijusted_R^2
Exercise type	6	-0.67 (-1.61, 0.28)	90.6	0.167	0.62	-0.2177	10	-0.45 (-0.92, 0.01)	78	0.057	0.22	*-0.1070*	7	0.84 (-0.87, 2.55)	95.6	0.335	2.13	-0.1985
Intensity	6	-0.54 (-1.87, 0.78)	88.6	0.42	0.54	0.0591	10	-0.13 (-0.57, 0.32)	81.9	0.577	0.29	-0.1009	7	0.06 (-1.32, 1.45)	95.1	0.929	1.92	-0.1939
Duration	6	0.02 (-0.03, 0.06)	88.1	0.467	0.57	0.0192	10	0.03 (-0.07, 0.13)	82.4	0.584	0.29	-0.1219	7	**0.24 (0.01, 0.48)**	91.2	0.039	0.97	0.0390
Frequency	6	0.09 (-0.32, 0.51)	89.2	0.664	0.62	-0.0984	9	0.04 (-0.25, 0.34)	84.3	0.778	0.35	-0.0970	6	-0.91 (-2.03, 0.21)	92.8	0.11	1.46	0.0741
Age	6	0.31 (-0.46, 1.09)	89.4	0.428	0.55	-0.0862	10	-0.18 (-0.67, 0.32)	81.8	0.485	0.29	-0.1233	7	-0.10 (-1.54, 1.34)	94.9	0.892	1.92	-0.1781
Depression severity	6	0.01 (-0.02, 0.05)	89.9	0.531	0.58	-0.0557	10	-0.00 (-0.02, 0.02)	83	0.925	0.3	-0.1232	7	0.03 (-0.03, 0.08)	94.3	0.33	1.6	-0.1798

Note: Bold values indicate $p < 0.05$ in the adjusted mod.

Table S8. Multivariable Meta-Regression on the Association between Exercise Training and Biomarkers in Adults with Major Depressive Disorder.

	k	Coefficient(95%Cl)	I^2
IL-6	21	0.46 (-0.78, 1.70)	0.468
CRP	21	-0.55 (-4.43, 3.34)	0.783
TNF-α	21	0.86 (-0.59, 2.32)	0.246
Intensity	21	-0.32 (-1.13, 0.50)	0.447
Duration	21	0.01 (-0.07, 0.08)	0.869
Frequency	21	0.03 (-0.48, 0.55)	0.9
Depression severity	21	0.02 (-0.74, 0.78)	0.959
Age	21	0.02 (-0.02, 0.06)	0.248
Female proportion	21	-2.18 (-5.26, 0.90)	0.166

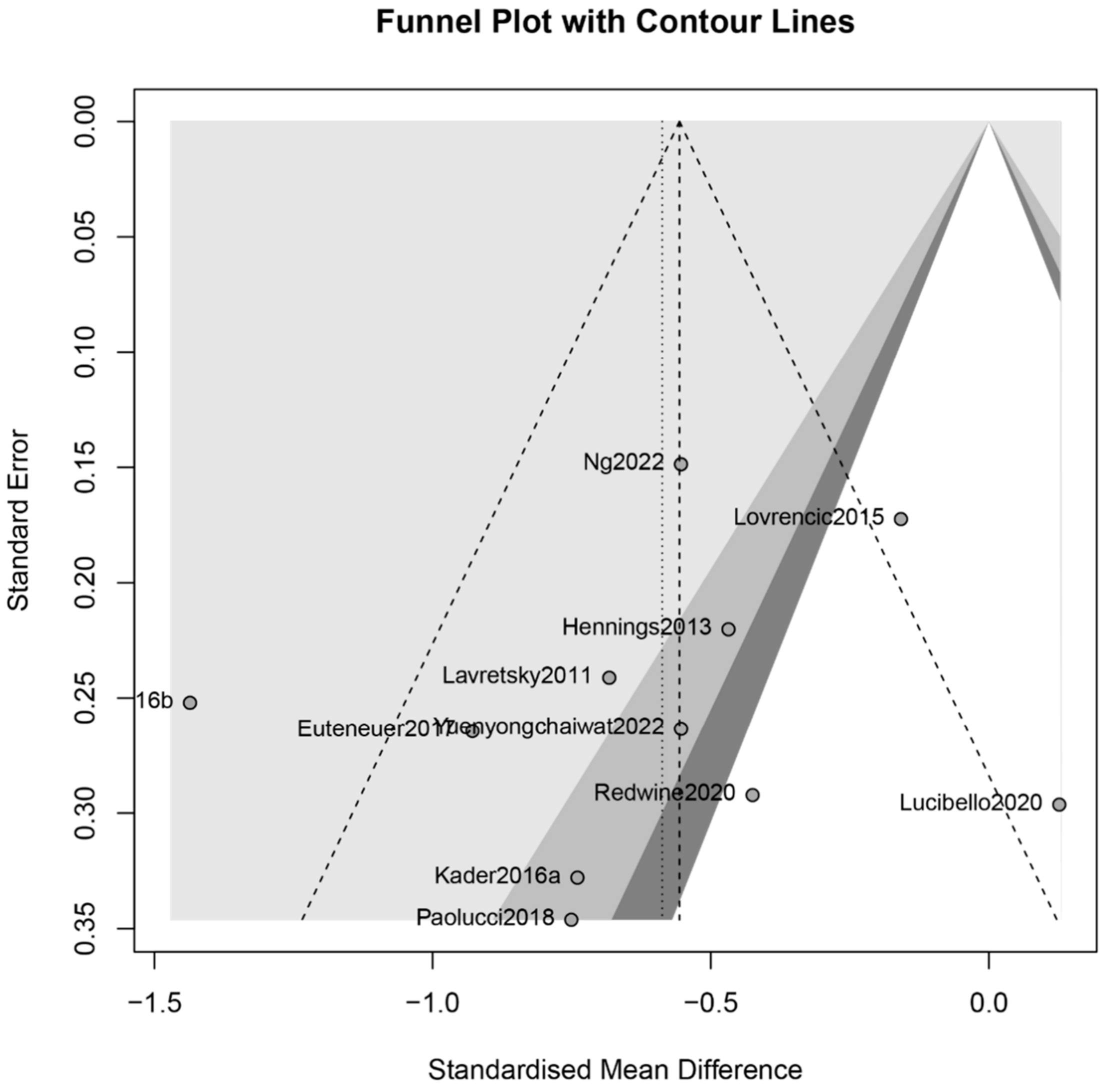

a

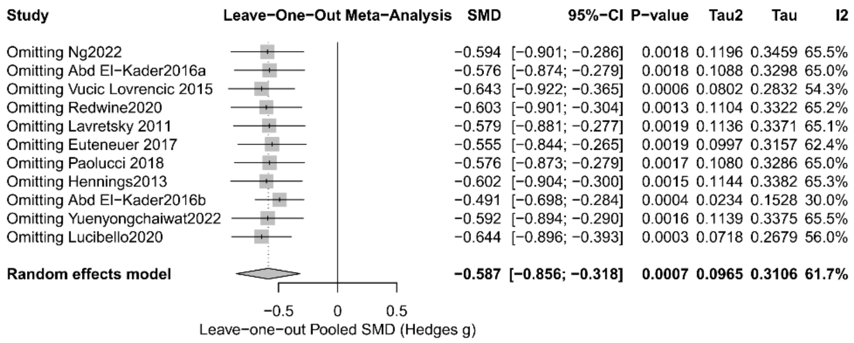

b

Figure S1. Publication Bias and Sensitivity Analysis of Exercise Effects on Depressive

Symptoms. (**a**) Funnel plot for visual inspection of publication bias in studies assessing the effect of exercise on depressive symptom; (**b**) Leave-one-out analysis of studies assessing the effect of exercise on depressive symptom, using the random effects model. The references in Figure S1 are Ng2022 [51], Abd El-Kader2016a[69], Vučić Lovrenčić 2015[76], Redwine2020[72], Lavretsky 2011[71], Euteneuer 2017[50], Paolucci 2018[49], Hennings2013[77], Abd El-Kader2016b[70], Yuenyongchaiwat2022[74], Lucibello2020[75].

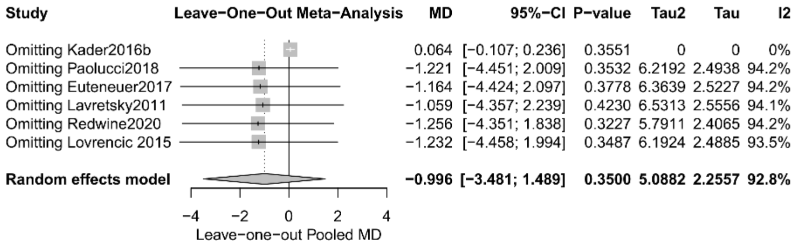

a

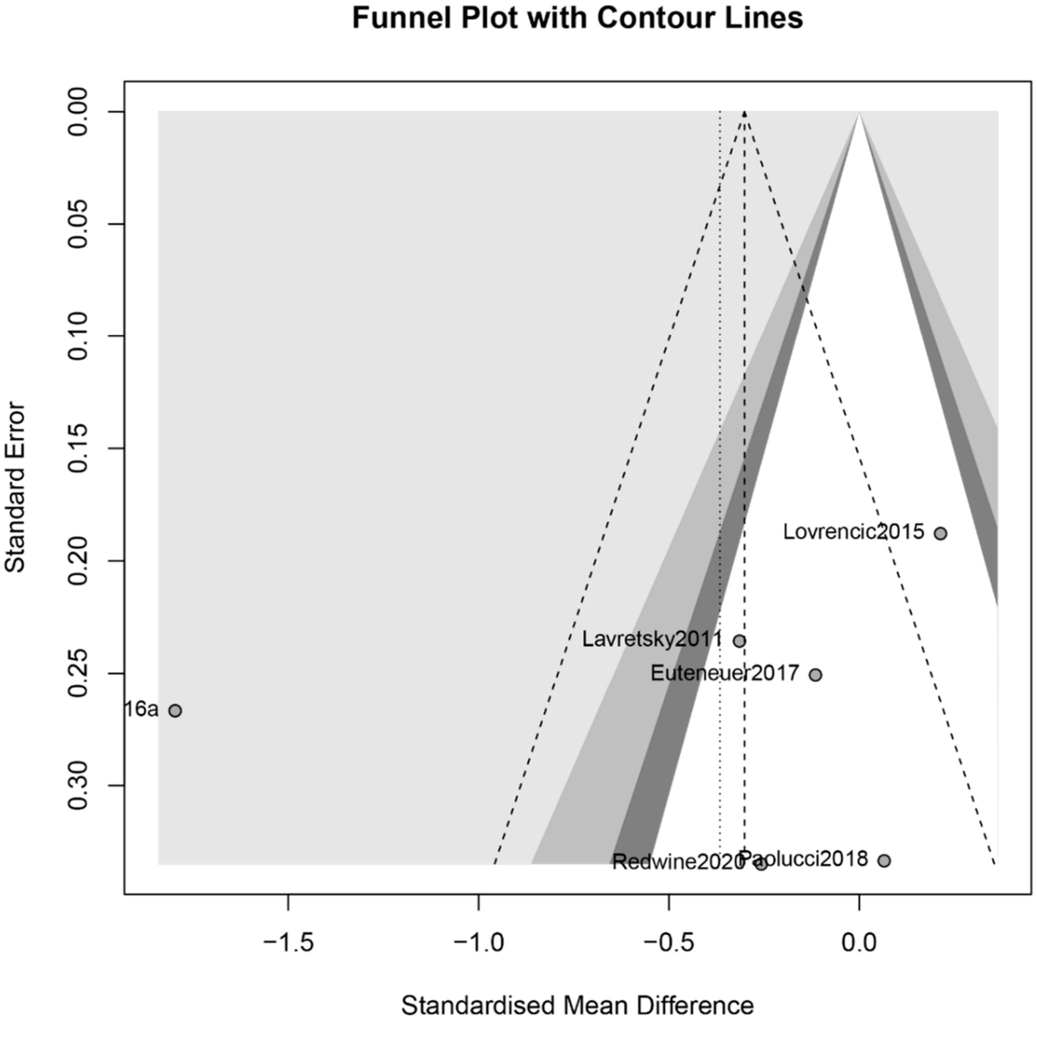

b

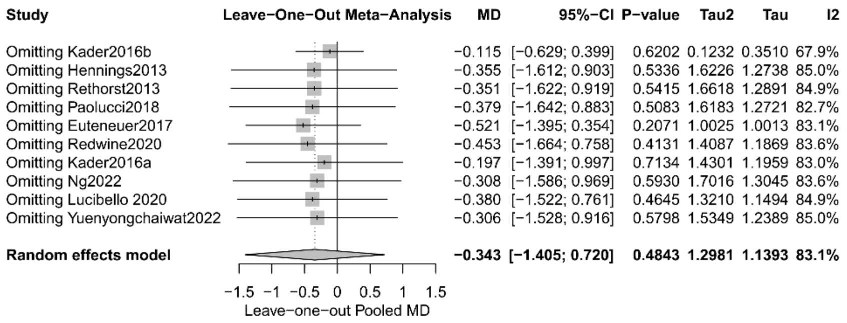

c

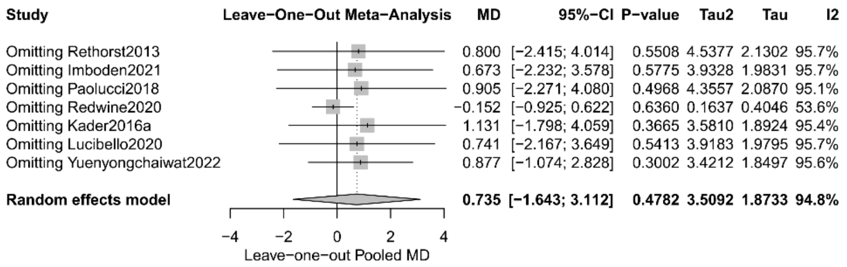

d

Figure S2: Publication Bias and Sensitivity Analyses of Exercise Effects on Inflammatory Biomarkers. (**a**) Leave-one-out analysis of studies assessing the effect of exercise on the level of CRP, using the random effects model; (**b**) Funnel plot for visual inspection of publication bias in studies assessing the effect of exercise on the level of IL-6; (**c**) Leave-one-out analysis of studies assessing the effect of exercise on the level of IL-6, using the random effects model; (**d**) Leave-one-out analysis of studies assessing the effect of exercise on the level of TNF-α, using the random effects model. The references in Figure S2 are Ng2022 [51], Abd El-Kader2016a[69], Vučić Lovrenčić 2015[76], Redwine2020[72], Lavretsky 2011[71], Euteneuer 2017[50], Paolucci 2018[49], Hennings2013[77], Abd El-Kader2016b[70], Yuenyongchaiwat2022[74], Lucibello2020[75], Rethorst2013[52], Imbo-den2021[73].

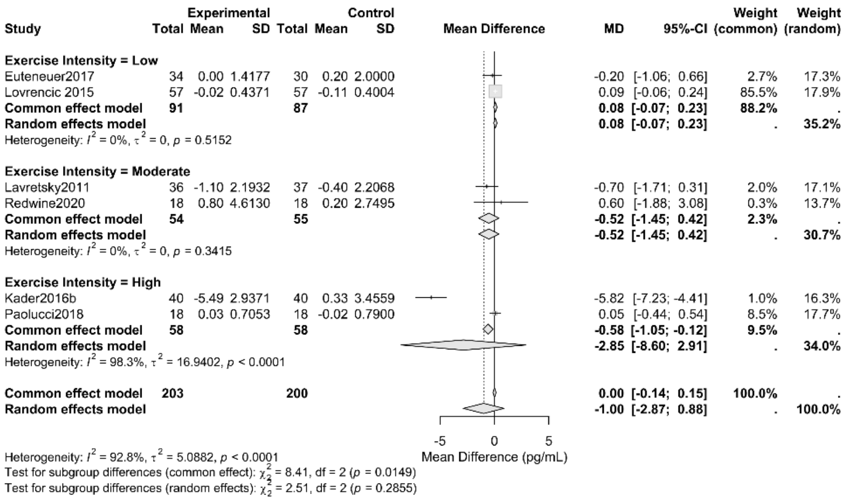
Study
Experimental Total Mean SD
Control Total Mean SD
Mean Difference
MD 95%-CI
Weight (common)
Weight (random)
Exercise Intensity = Low
Euteneuer2017 34 0.00 1.4177 30 0.20 2.0000 -0.20 [-1.06; 0.66] 2.7% 17.3%
Lovrencic 2015 57 -0.02 0.4371 57 -0.11 0.4004 0.09 [-0.06; 0.24] 85.5% 17.9%
Common effect model 91 87 0.08 [-0.07; 0.23] 88.2% .
Random effects model 0.08 [-0.07; 0.23] . 35.2%
Heterogeneity: I² = 0%, τ² = 0, p = 0.5152
Exercise Intensity = Moderate
Lavretsky2011 36 -1.10 2.1932 37 -0.40 2.2068 -0.70 [-1.71; 0.31] 2.0% 17.1%
Redwine2020 18 0.80 4.6130 18 0.20 2.7495 0.60 [-1.88; 3.08] 0.3% 13.7%
Common effect model 54 55 -0.52 [-1.45; 0.42] 2.3% .
Random effects model -0.52 [-1.45; 0.42] . 30.7%
Heterogeneity: I² = 0%, τ² = 0, p = 0.3415
Exercise Intensity = High
Kader2016b 40 -5.49 2.9371 40 0.33 3.4559 -5.82 [-7.23; -4.41] 1.0% 16.3%
Paolucci2018 18 0.03 0.7053 18 -0.02 0.7900 0.05 [-0.44; 0.54] 8.5% 17.7%
Common effect model 58 58 -0.58 [-1.05; -0.12] 9.5% .
Random effects model -2.85 [-8.60; 2.91] . 34.0%
Heterogeneity: I² = 98.3%, τ² = 16.9402, p < 0.0001
Common effect model 203 200 0.00 [-0.14; 0.15] 100.0% .
Random effects model -1.00 [-2.87; 0.88] . 100.0%
-5 0 5
Mean Difference (pg/mL)
Heterogeneity: I² = 92.8%, τ² = 5.0882, p < 0.0001
Test for subgroup differences (common effect): χ²₂ = 8.41, df = 2 (p = 0.0149)
Test for subgroup differences (random effects): χ²₂ = 2.51, df = 2 (p = 0.2855)

a

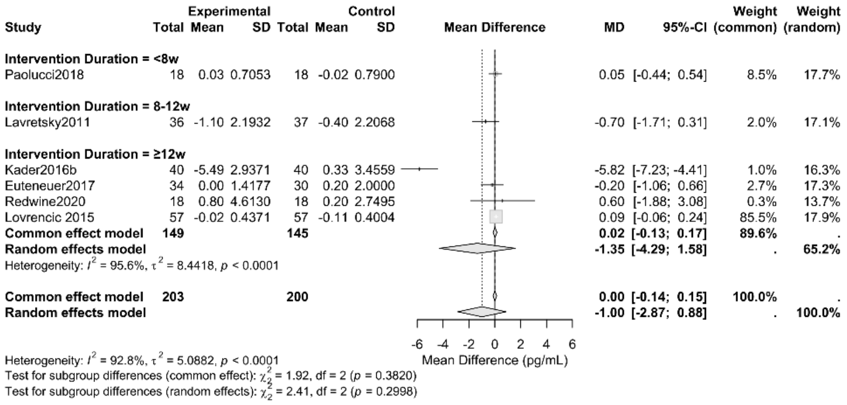
Study
Experimental Total Mean SD
Control Total Mean SD
Mean Difference
MD 95%-CI
Weight (common)
Weight (random)
Intervention Duration = <8w
Paolucci2018 18 0.03 0.7053 18 -0.02 0.7900 0.05 [-0.44; 0.54] 8.5% 17.7%
Intervention Duration = 8-12w
Lavretsky2011 36 -1.10 2.1932 37 -0.40 2.2068 -0.70 [-1.71; 0.31] 2.0% 17.1%
Intervention Duration = ≥12w
Kader2016b 40 -5.49 2.9371 40 0.33 3.4559 -5.82 [-7.23; -4.41] 1.0% 16.3%
Euteneuer2017 34 0.00 1.4177 30 0.20 2.0000 -0.20 [-1.06; 0.66] 2.7% 17.3%
Redwine2020 18 0.80 4.6130 18 0.20 2.7495 0.60 [-1.88; 3.08] 0.3% 13.7%
Lovrencic 2015 57 -0.02 0.4371 57 -0.11 0.4004 0.09 [-0.06; 0.24] 85.5% 17.9%
Common effect model 149 145 0.02 [-0.13; 0.17] 89.6% .
Random effects model -1.35 [-4.29; 1.58] . 65.2%
Heterogeneity: I² = 95.6%, τ² = 8.4418, p < 0.0001
Common effect model 203 200 0.00 [-0.14; 0.15] 100.0% .
Random effects model -1.00 [-2.87; 0.88] . 100.0%
-6 -4 -2 0 2 4 6
Mean Difference (pg/mL)
Heterogeneity: I² = 92.8%, τ² = 5.0882, p < 0.0001
Test for subgroup differences (common effect): χ²₂ = 1.92, df = 2 (p = 0.3820)
Test for subgroup differences (random effects): χ²₂ = 2.41, df = 2 (p = 0.2998)

b

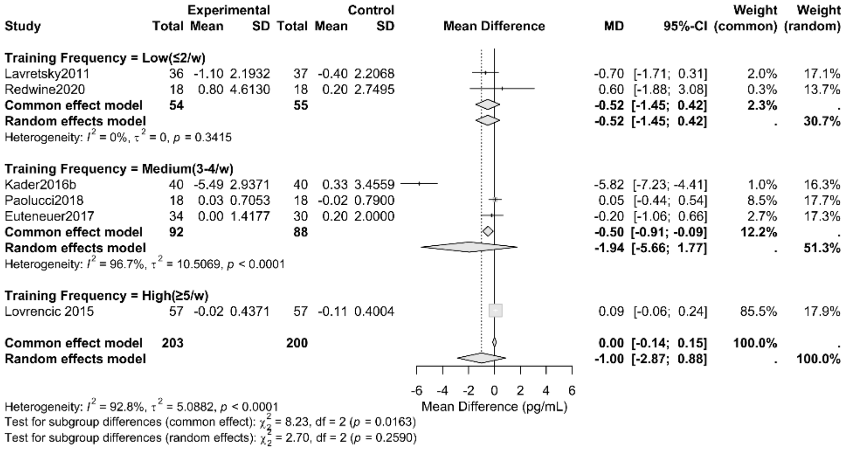
Study
Experimental Total Mean SD
Control Total Mean SD
Mean Difference
MD 95%-CI
Weight (common)
Weight (random)
Training Frequency = Low(≤2/w)
Lavretsky2011 36 -1.10 2.1932 37 -0.40 2.2068 -0.70 [-1.71; 0.31] 2.0% 17.1%
Redwine2020 18 0.80 4.6130 18 0.20 2.7495 0.60 [-1.88; 3.08] 0.3% 13.7%
Common effect model 54 55 -0.52 [-1.45; 0.42] 2.3% .
Random effects model -0.52 [-1.45; 0.42] . 30.7%
Heterogeneity: I² = 0%, τ² = 0, p = 0.3415
Training Frequency = Medium(3-4/w)
Kader2016b 40 -5.49 2.9371 40 0.33 3.4559 -5.82 [-7.23; -4.41] 1.0% 16.3%
Paolucci2018 18 0.03 0.7053 18 -0.02 0.7900 0.05 [-0.44; 0.54] 8.5% 17.7%
Euteneuer2017 34 0.00 1.4177 30 0.20 2.0000 -0.20 [-1.06; 0.66] 2.7% 17.3%
Common effect model 92 88 -0.50 [-0.91; -0.09] 12.2% .
Random effects model -1.94 [-5.66; 1.77] . 51.3%
Heterogeneity: I² = 96.7%, τ² = 10.5069, p < 0.0001
Training Frequency = High(≥5/w)
Lovrencic 2015 57 -0.02 0.4371 57 -0.11 0.4004 0.09 [-0.06; 0.24] 85.5% 17.9%
Common effect model 203 200 0.00 [-0.14; 0.15] 100.0% .
Random effects model -1.00 [-2.87; 0.88] . 100.0%
-6 -4 -2 0 2 4 6
Mean Difference (pg/mL)
Heterogeneity: I² = 92.8%, τ² = 5.0882, p < 0.0001
Test for subgroup differences (common effect): χ²₂ = 8.23, df = 2 (p = 0.0163)
Test for subgroup differences (random effects): χ²₂ = 2.70, df = 2 (p = 0.2590)

c

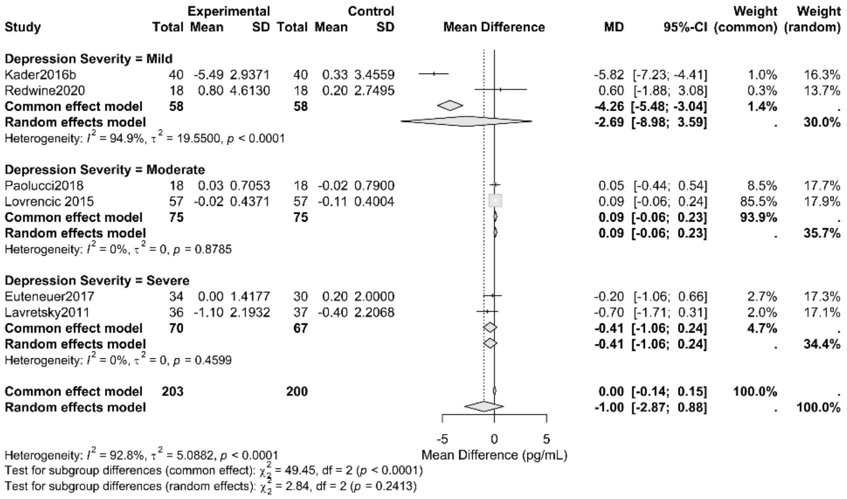
Experimental
Control
Study Total Mean SD Total Mean SD
Mean Difference
MD 95%-CI
Weight (common)
Weight (random)
Depression Severity = Mild
Kader2016b 40 -5.49 2.9371 40 0.33 3.4559 -5.82 [-7.23; -4.41] 1.0% 16.3%
Redwine2020 18 0.80 4.6130 18 0.20 2.7495 0.60 [-1.88; 3.08] 0.3% 13.7%
Common effect model 58 58 -4.26 [-5.48; -3.04] 1.4% .
Random effects model -2.69 [-8.98; 3.59] . 30.0%
Heterogeneity: I^2 = 94.9%, τ^2 = 19.5500, p < 0.0001
Depression Severity = Moderate
Paolucci2018 18 0.03 0.7053 18 -0.02 0.7900 0.05 [-0.44; 0.54] 8.5% 17.7%
Lovrencic 2015 57 -0.02 0.4371 57 -0.11 0.4004 0.09 [-0.06; 0.24] 85.5% 17.9%
Common effect model 75 75 0.09 [-0.06; 0.23] 93.9% .
Random effects model 0.09 [-0.06; 0.23] . 35.7%
Heterogeneity: I^2 = 0%, τ^2 = 0, p = 0.8785
Depression Severity = Severe
Euteneuer2017 34 0.00 1.4177 30 0.20 2.0000 -0.20 [-1.06; 0.66] 2.7% 17.3%
Lavretsky2011 36 -1.10 2.1932 37 -0.40 2.2068 -0.70 [-1.71; 0.31] 2.0% 17.1%
Common effect model 70 67 -0.41 [-1.06; 0.24] 4.7% .
Random effects model -0.41 [-1.06; 0.24] . 34.4%
Heterogeneity: I^2 = 0%, τ^2 = 0, p = 0.4599
Common effect model 203 200 0.00 [-0.14; 0.15] 100.0% .
Random effects model -1.00 [-2.87; 0.88] . 100.0%
-5 0 5
Mean Difference (pg/mL)
Heterogeneity: I^2 = 92.8%, τ^2 = 5.0882, p < 0.0001
Test for subgroup differences (common effect): χ^2_2 = 49.45, df = 2 (p < 0.0001)
Test for subgroup differences (random effects): χ^2_2 = 2.84, df = 2 (p = 0.2413)

d

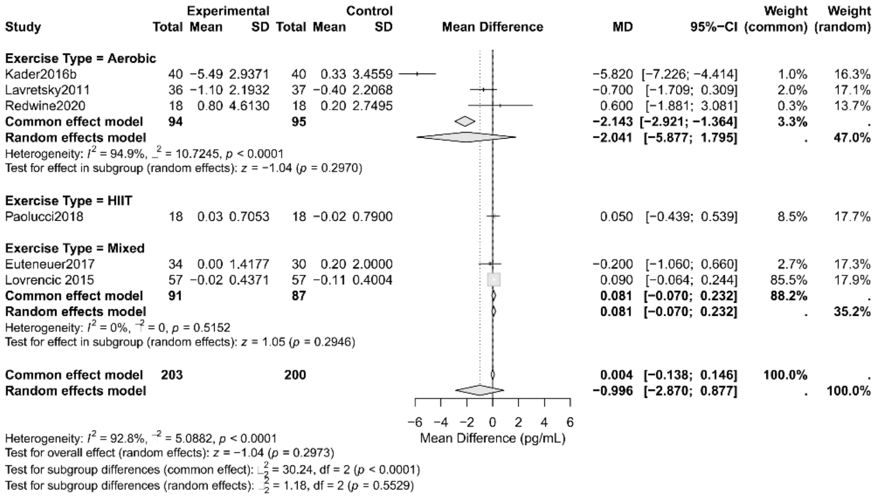
Experimental
Control
Study Total Mean SD Total Mean SD
Mean Difference
MD 95%−CI
Weight (common)
Weight (random)
Exercise Type = Aerobic
Kader2016b 40 −5.49 2.9371 40 0.33 3.4559 −5.820 [−7.226; −4.414] 1.0% 16.3%
Lavretsky2011 36 −1.10 2.1932 37 −0.40 2.2068 −0.700 [−1.709; 0.309] 2.0% 17.1%
Redwine2020 18 0.80 4.6130 18 0.20 2.7495 0.600 [−1.881; 3.081] 0.3% 13.7%
Common effect model 94 95 −2.143 [−2.921; −1.364] 3.3% .
Random effects model −2.041 [−5.877; 1.795] . 47.0%
Heterogeneity: I^2 = 94.9%, _^2 = 10.7245, p < 0.0001
Test for effect in subgroup (random effects): z = −1.04 (p = 0.2970)
Exercise Type = HIIT
Paolucci2018 18 0.03 0.7053 18 −0.02 0.7900 0.050 [−0.439; 0.539] 8.5% 17.7%
Exercise Type = Mixed
Euteneuer2017 34 0.00 1.4177 30 0.20 2.0000 −0.200 [−1.060; 0.660] 2.7% 17.3%
Lovrencic 2015 57 −0.02 0.4371 57 −0.11 0.4004 0.090 [−0.064; 0.244] 85.5% 17.9%
Common effect model 91 87 0.081 [−0.070; 0.232] 88.2% .
Random effects model 0.081 [−0.070; 0.232] . 35.2%
Heterogeneity: I^2 = 0%, _^2 = 0, p = 0.5152
Test for effect in subgroup (random effects): z = 1.05 (p = 0.2946)
Common effect model 203 200 0.004 [−0.138; 0.146] 100.0% .
Random effects model −0.996 [−2.870; 0.877] . 100.0%
−6 −4 −2 0 2 4 6
Mean Difference (pg/mL)
Heterogeneity: I^2 = 92.8%, _^2 = 5.0882, p < 0.0001
Test for overall effect (random effects): z = −1.04 (p = 0.2973)
Test for subgroup differences (common effect): _^2_2 = 30.24, df = 2 (p < 0.0001)
Test for subgroup differences (random effects): _^2_2 = 1.18, df = 2 (p = 0.5529)

e

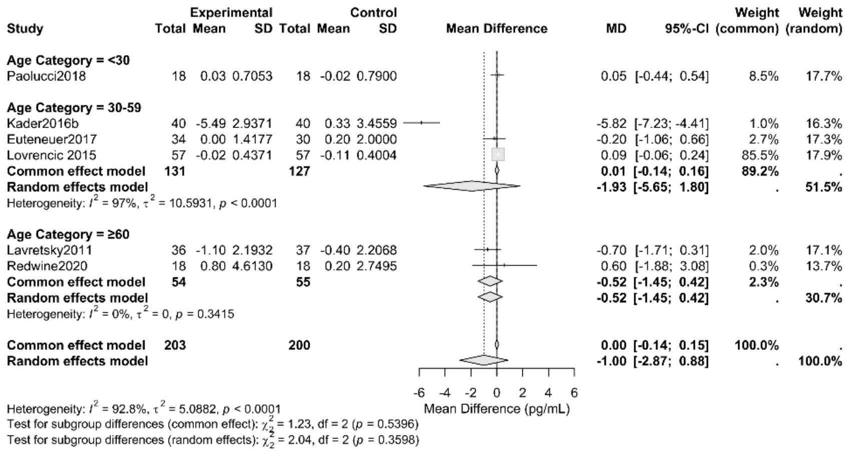
Study | Experimental Total Mean SD | Control Total Mean SD | Mean Difference | MD 95%-CI | Weight (common) | Weight (random)
Age Category = <30
Paolucci2018 18 0.03 0.7053 18 -0.02 0.7900 0.05 [-0.44; 0.54] 8.5% 17.7%
Age Category = 30-59
Kader2016b 40 -5.49 2.9371 40 0.33 3.4559 -5.82 [-7.23; -4.41] 1.0% 16.3%
Euteneuer2017 34 0.00 1.4177 30 0.20 2.0000 -0.20 [-1.06; 0.66] 2.7% 17.3%
Lovrencic 2015 57 -0.02 0.4371 57 -0.11 0.4004 0.09 [-0.06; 0.24] 85.5% 17.9%
Common effect model 131 127 0.01 [-0.14; 0.16] 89.2% .
Random effects model -1.93 [-5.65; 1.80] . 51.5%
Heterogeneity: I^2 = 97%, τ^2 = 10.5931, p < 0.0001
Age Category = ≥60
Lavretsky2011 36 -1.10 2.1932 37 -0.40 2.2068 -0.70 [-1.71; 0.31] 2.0% 17.1%
Redwine2020 18 0.80 4.6130 18 0.20 2.7495 0.60 [-1.88; 3.08] 0.3% 13.7%
Common effect model 54 55 -0.52 [-1.45; 0.42] 2.3% .
Random effects model -0.52 [-1.45; 0.42] . 30.7%
Heterogeneity: I^2 = 0%, τ^2 = 0, p = 0.3415
Common effect model 203 200 0.00 [-0.14; 0.15] 100.0% .
Random effects model -1.00 [-2.87; 0.88] . 100.0%
-6 -4 -2 0 2 4 6
Mean Difference (pg/mL)
Heterogeneity: I^2 = 92.8%, τ^2 = 5.0882, p < 0.0001
Test for subgroup differences (common effect): χ^2_2 = 1.23, df = 2 (p = 0.5396)
Test for subgroup differences (random effects): χ^2_2 = 2.04, df = 2 (p = 0.3598)

f

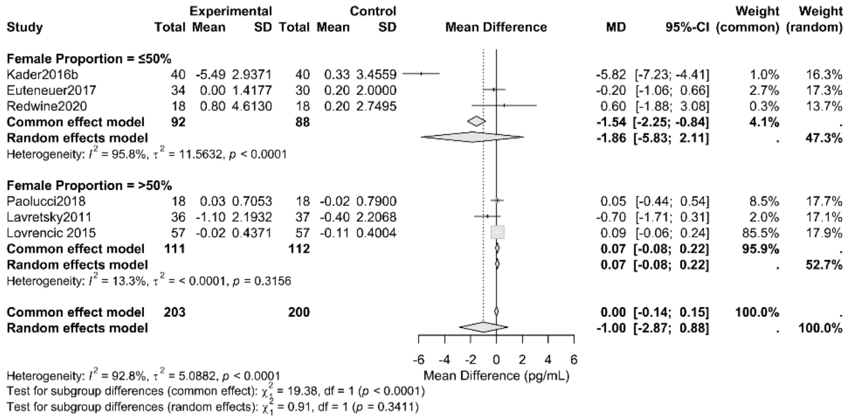
Study | Experimental Total Mean SD | Control Total Mean SD | Mean Difference | MD 95%-CI | Weight (common) | Weight (random)
Female Proportion = ≤50%
Kader2016b 40 -5.49 2.9371 40 0.33 3.4559 -5.82 [-7.23; -4.41] 1.0% 16.3%
Euteneuer2017 34 0.00 1.4177 30 0.20 2.0000 -0.20 [-1.06; 0.66] 2.7% 17.3%
Redwine2020 18 0.80 4.6130 18 0.20 2.7495 0.60 [-1.88; 3.08] 0.3% 13.7%
Common effect model 92 88 -1.54 [-2.25; -0.84] 4.1% .
Random effects model -1.86 [-5.83; 2.11] . 47.3%
Heterogeneity: I^2 = 95.8%, τ^2 = 11.5632, p < 0.0001
Female Proportion = >50%
Paolucci2018 18 0.03 0.7053 18 -0.02 0.7900 0.05 [-0.44; 0.54] 8.5% 17.7%
Lavretsky2011 36 -1.10 2.1932 37 -0.40 2.2068 -0.70 [-1.71; 0.31] 2.0% 17.1%
Lovrencic 2015 57 -0.02 0.4371 57 -0.11 0.4004 0.09 [-0.06; 0.24] 85.5% 17.9%
Common effect model 111 112 0.07 [-0.08; 0.22] 95.9% .
Random effects model 0.07 [-0.08; 0.22] . 52.7%
Heterogeneity: I^2 = 13.3%, τ^2 = < 0.0001, p = 0.3156
Common effect model 203 200 0.00 [-0.14; 0.15] 100.0% .
Random effects model -1.00 [-2.87; 0.88] . 100.0%
-6 -4 -2 0 2 4 6
Mean Difference (pg/mL)
Heterogeneity: I^2 = 92.8%, τ^2 = 5.0882, p < 0.0001
Test for subgroup differences (common effect): χ^2_1 = 19.38, df = 1 (p < 0.0001)
Test for subgroup differences (random effects): χ^2_1 = 0.91, df = 1 (p = 0.3411)

G

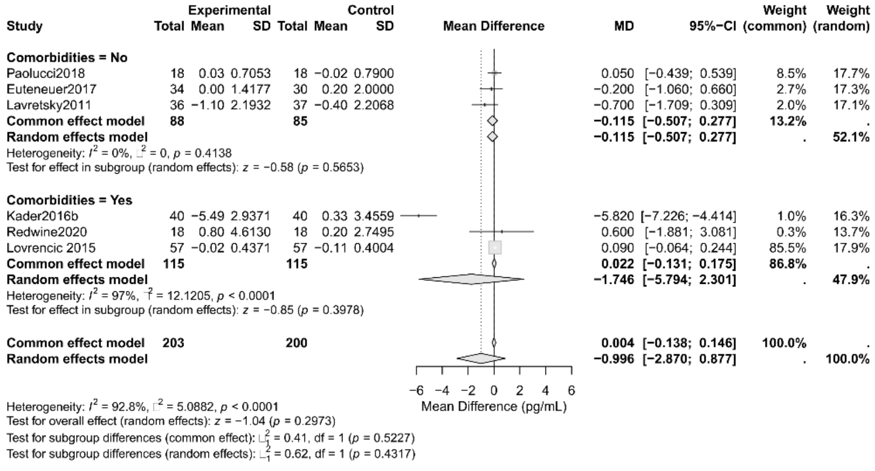
Study | Experimental Total Mean SD | Control Total Mean SD | Mean Difference | MD 95%−CI | Weight (common) | Weight (random)
Comorbidities = No
Paolucci2018 18 0.03 0.7053 18 −0.02 0.7900 0.050 [−0.439; 0.539] 8.5% 17.7%
Euteneuer2017 34 0.00 1.4177 30 0.20 2.0000 −0.200 [−1.060; 0.660] 2.7% 17.3%
Lavretsky2011 36 −1.10 2.1932 37 −0.40 2.2068 −0.700 [−1.709; 0.309] 2.0% 17.1%
Common effect model 88 85 −0.115 [−0.507; 0.277] 13.2% .
Random effects model −0.115 [−0.507; 0.277] . 52.1%
Heterogeneity: I^2 = 0%, τ^2 = 0, p = 0.4138
Test for effect in subgroup (random effects): z = −0.58 (p = 0.5653)
Comorbidities = Yes
Kader2016b 40 −5.49 2.9371 40 0.33 3.4559 −5.820 [−7.226; −4.414] 1.0% 16.3%
Redwine2020 18 0.80 4.6130 18 0.20 2.7495 0.600 [−1.881; 3.081] 0.3% 13.7%
Lovrencic 2015 57 −0.02 0.4371 57 −0.11 0.4004 0.090 [−0.064; 0.244] 85.5% 17.9%
Common effect model 115 115 0.022 [−0.131; 0.175] 86.8% .
Random effects model −1.746 [−5.794; 2.301] . 47.9%
Heterogeneity: I^2 = 97%, τ^2 = 12.1205, p < 0.0001
Test for effect in subgroup (random effects): z = −0.85 (p = 0.3978)
Common effect model 203 200 0.004 [−0.138; 0.146] 100.0% .
Random effects model −0.996 [−2.870; 0.877] . 100.0%
−6 −4 −2 0 2 4 6
Mean Difference (pg/mL)
Heterogeneity: I^2 = 92.8%, τ^2 = 5.0882, p < 0.0001
Test for overall effect (random effects): z = −1.04 (p = 0.2973)
Test for subgroup differences (common effect): χ^2_1 = 0.41, df = 1 (p = 0.5227)
Test for subgroup differences (random effects): χ^2_1 = 0.62, df = 1 (p = 0.4317)

h

Figure S3: Subgroup Analysis of Exercise-Induced CRP Level Changes by Intervention Parameters and Participant Characteristics. (**a**) Forest plot of the mean difference in CRP levels at different exercise intensities; (**b**) Forest plot of the mean difference in CRP levels at different exercise length; (**c**) Forest plot of the mean difference in CRP levels at different exercise frequency; (**d**) Forest plot of the mean difference in CRP levels at different depression severities; (**e**) Forest plot of the mean difference in CRP levels at different exercise types; (**f**) Forest plot of the mean difference in CRP levels at different age ranges; (**g**) Forest plot of the mean difference in CRP levels at different proportion of females; **(h)** Forest plot of the mean difference in CRP levels in the presence or absence of comorbidities. The references in Figure S3 are Ng2022 [51], Abd El-Kader2016a[69], Vučić Lovrenčić 2015[76], Redwine2020[72], Lavretsky 2011[71], Euteneuer 2017[50], Paolucci 2018[49], Hennings2013[77], Abd El-Kader2016b[70], Yuenyongchaiwat2022[74], Lucibello2020[75], Rethorst2013[52], Imbo-den2021[73].

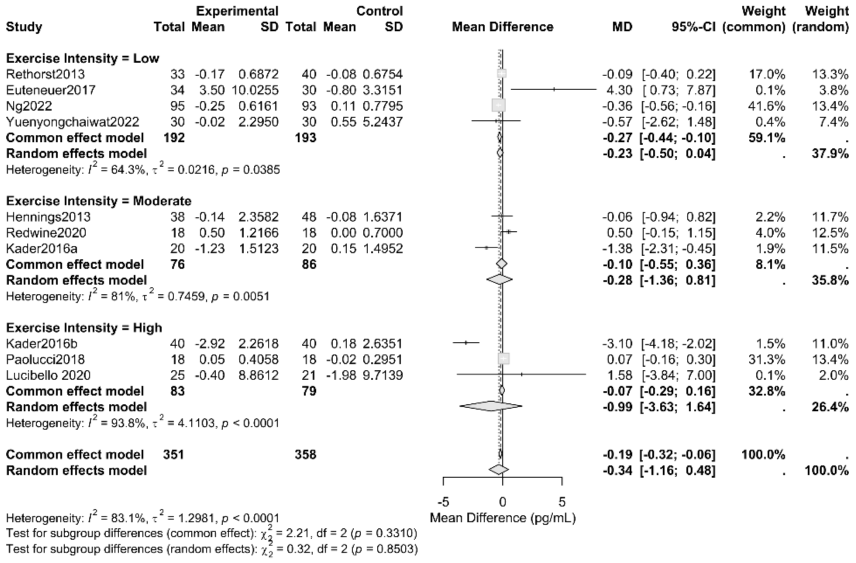

a

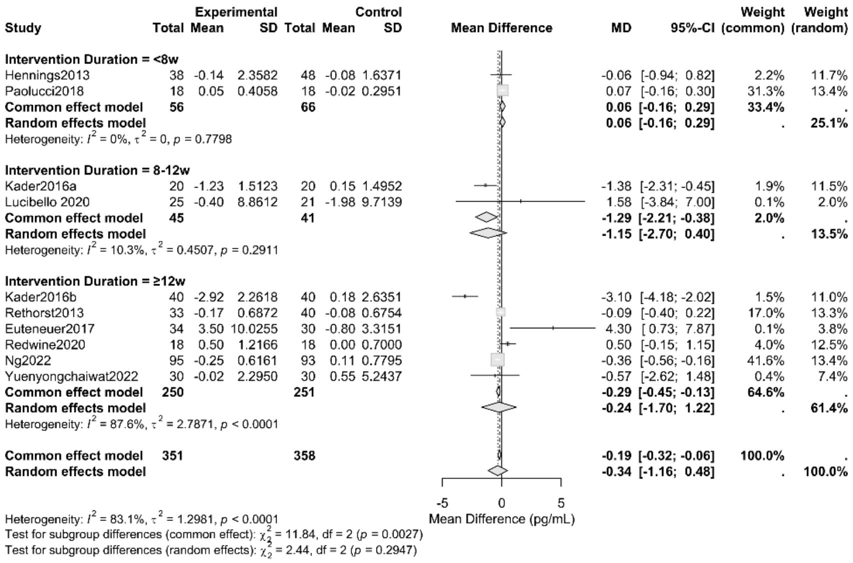
Experimental
Control
Weight
Weight
Study
Total Mean SD Total Mean SD
Mean Difference
MD 95%-CI (common) (random)
Intervention Duration = <8w
Hennings2013 38 -0.14 2.3582 48 -0.08 1.6371 -0.06 [-0.94; 0.82] 2.2% 11.7%
Paolucci2018 18 0.05 0.4058 18 -0.02 0.2951 0.07 [-0.16; 0.30] 31.3% 13.4%
Common effect model 56 66 0.06 [-0.16; 0.29] 33.4% .
Random effects model 0.06 [-0.16; 0.29] . 25.1%
Heterogeneity: I2 = 0%, τ2 = 0, p = 0.7798
Intervention Duration = 8-12w
Kader2016a 20 -1.23 1.5123 20 0.15 1.4952 -1.38 [-2.31; -0.45] 1.9% 11.5%
Lucibello 2020 25 -0.40 8.8612 21 -1.98 9.7139 1.58 [-3.84; 7.00] 0.1% 2.0%
Common effect model 45 41 -1.29 [-2.21; -0.38] 2.0% .
Random effects model -1.15 [-2.70; 0.40] . 13.5%
Heterogeneity: I2 = 10.3%, τ2 = 0.4507, p = 0.2911
Intervention Duration = ≥12w
Kader2016b 40 -2.92 2.2618 40 0.18 2.6351 -3.10 [-4.18; -2.02] 1.5% 11.0%
Rethorst2013 33 -0.17 0.6872 40 -0.08 0.6754 -0.09 [-0.40; 0.22] 17.0% 13.3%
Euteneuer2017 34 3.50 10.0255 30 -0.80 3.3151 4.30 [0.73; 7.87] 0.1% 3.8%
Redwine2020 18 0.50 1.2166 18 0.00 0.7000 0.50 [-0.15; 1.15] 4.0% 12.5%
Ng2022 95 -0.25 0.6161 93 0.11 0.7795 -0.36 [-0.56; -0.16] 41.6% 13.4%
Yuenyongchaiwat2022 30 -0.02 2.2950 30 0.55 5.2437 -0.57 [-2.62; 1.48] 0.4% 7.4%
Common effect model 250 251 -0.29 [-0.45; -0.13] 64.6% .
Random effects model -0.24 [-1.70; 1.22] . 61.4%
Heterogeneity: I2 = 87.6%, τ2 = 2.7871, p < 0.0001
Common effect model 351 358 -0.19 [-0.32; -0.06] 100.0% .
Random effects model -0.34 [-1.16; 0.48] . 100.0%
-5 0 5
Mean Difference (pg/mL)
Heterogeneity: I2 = 83.1%, τ2 = 1.2981, p < 0.0001
Test for subgroup differences (common effect): χ22 = 11.84, df = 2 (p = 0.0027)
Test for subgroup differences (random effects): χ22 = 2.44, df = 2 (p = 0.2947)

b

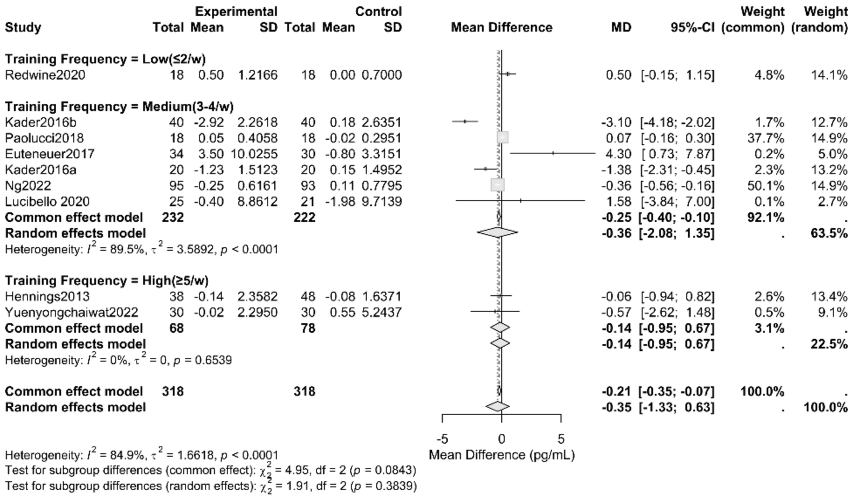
Experimental
Control
Weight
Weight
Study
Total Mean SD Total Mean SD
Mean Difference
MD 95%-CI (common) (random)
Training Frequency = Low(≤2/w)
Redwine2020 18 0.50 1.2166 18 0.00 0.7000 0.50 [-0.15; 1.15] 4.8% 14.1%
Training Frequency = Medium(3-4/w)
Kader2016b 40 -2.92 2.2618 40 0.18 2.6351 -3.10 [-4.18; -2.02] 1.7% 12.7%
Paolucci2018 18 0.05 0.4058 18 -0.02 0.2951 0.07 [-0.16; 0.30] 37.7% 14.9%
Euteneuer2017 34 3.50 10.0255 30 -0.80 3.3151 4.30 [0.73; 7.87] 0.2% 5.0%
Kader2016a 20 -1.23 1.5123 20 0.15 1.4952 -1.38 [-2.31; -0.45] 2.3% 13.2%
Ng2022 95 -0.25 0.6161 93 0.11 0.7795 -0.36 [-0.56; -0.16] 50.1% 14.9%
Lucibello 2020 25 -0.40 8.8612 21 -1.98 9.7139 1.58 [-3.84; 7.00] 0.1% 2.7%
Common effect model 232 222 -0.25 [-0.40; -0.10] 92.1% .
Random effects model -0.36 [-2.08; 1.35] . 63.5%
Heterogeneity: I2 = 89.5%, τ2 = 3.5892, p < 0.0001
Training Frequency = High(≥5/w)
Hennings2013 38 -0.14 2.3582 48 -0.08 1.6371 -0.06 [-0.94; 0.82] 2.6% 13.4%
Yuenyongchaiwat2022 30 -0.02 2.2950 30 0.55 5.2437 -0.57 [-2.62; 1.48] 0.5% 9.1%
Common effect model 68 78 -0.14 [-0.95; 0.67] 3.1% .
Random effects model -0.14 [-0.95; 0.67] . 22.5%
Heterogeneity: I2 = 0%, τ2 = 0, p = 0.6539
Common effect model 318 318 -0.21 [-0.35; -0.07] 100.0% .
Random effects model -0.35 [-1.33; 0.63] . 100.0%
-5 0 5
Mean Difference (pg/mL)
Heterogeneity: I2 = 84.9%, τ2 = 1.6618, p < 0.0001
Test for subgroup differences (common effect): χ22 = 4.95, df = 2 (p = 0.0843)
Test for subgroup differences (random effects): χ22 = 1.91, df = 2 (p = 0.3839)

c

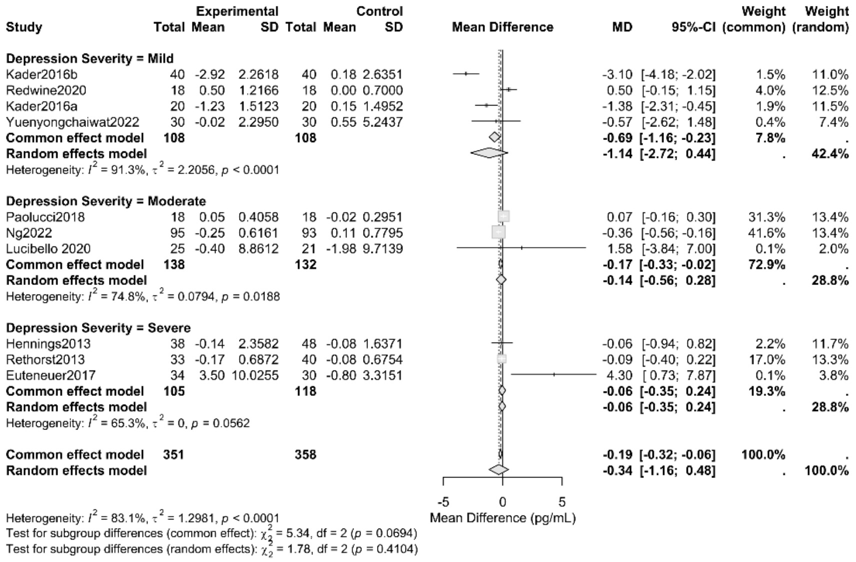

Study
Experimental Total Mean SD
Control Total Mean SD
Mean Difference
MD 95%-CI
Weight (common)
Weight (random)
Depression Severity = Mild
Kader2016b 40 -2.92 2.2618 40 0.18 2.6351 -3.10 [-4.18; -2.02] 1.5% 11.0%
Redwine2020 18 0.50 1.2166 18 0.00 0.7000 0.50 [-0.15; 1.15] 4.0% 12.5%
Kader2016a 20 -1.23 1.5123 20 0.15 1.4952 -1.38 [-2.31; -0.45] 1.9% 11.5%
Yuenyongchaiwat2022 30 -0.02 2.2950 30 0.55 5.2437 -0.57 [-2.62; 1.48] 0.4% 7.4%
Common effect model 108 108 -0.69 [-1.16; -0.23] 7.8% .
Random effects model -1.14 [-2.72; 0.44] . 42.4%
Heterogeneity: I^2 = 91.3%, τ^2 = 2.2056, p < 0.0001
Depression Severity = Moderate
Paolucci2018 18 0.05 0.4058 18 -0.02 0.2951 0.07 [-0.16; 0.30] 31.3% 13.4%
Ng2022 95 -0.25 0.6161 93 0.11 0.7795 -0.36 [-0.56; -0.16] 41.6% 13.4%
Lucibello 2020 25 -0.40 8.8612 21 -1.98 9.7139 1.58 [-3.84; 7.00] 0.1% 2.0%
Common effect model 138 132 -0.17 [-0.33; -0.02] 72.9% .
Random effects model -0.14 [-0.56; 0.28] . 28.8%
Heterogeneity: I^2 = 74.8%, τ^2 = 0.0794, p = 0.0188
Depression Severity = Severe
Hennings2013 38 -0.14 2.3582 48 -0.08 1.6371 -0.06 [-0.94; 0.82] 2.2% 11.7%
Rethorst2013 33 -0.17 0.6872 40 -0.08 0.6754 -0.09 [-0.40; 0.22] 17.0% 13.3%
Euteneuer2017 34 3.50 10.0255 30 -0.80 3.3151 4.30 [0.73; 7.87] 0.1% 3.8%
Common effect model 105 118 -0.06 [-0.35; 0.24] 19.3% .
Random effects model -0.06 [-0.35; 0.24] . 28.8%
Heterogeneity: I^2 = 65.3%, τ^2 = 0, p = 0.0562
Common effect model 351 358 -0.19 [-0.32; -0.06] 100.0% .
Random effects model -0.34 [-1.16; 0.48] . 100.0%
-5
0
5
Mean Difference (pg/mL)
Heterogeneity: I^2 = 83.1%, τ^2 = 1.2981, p < 0.0001
Test for subgroup differences (common effect): χ^2_2 = 5.34, df = 2 (p = 0.0694)
Test for subgroup differences (random effects): χ^2_2 = 1.78, df = 2 (p = 0.4104)

d

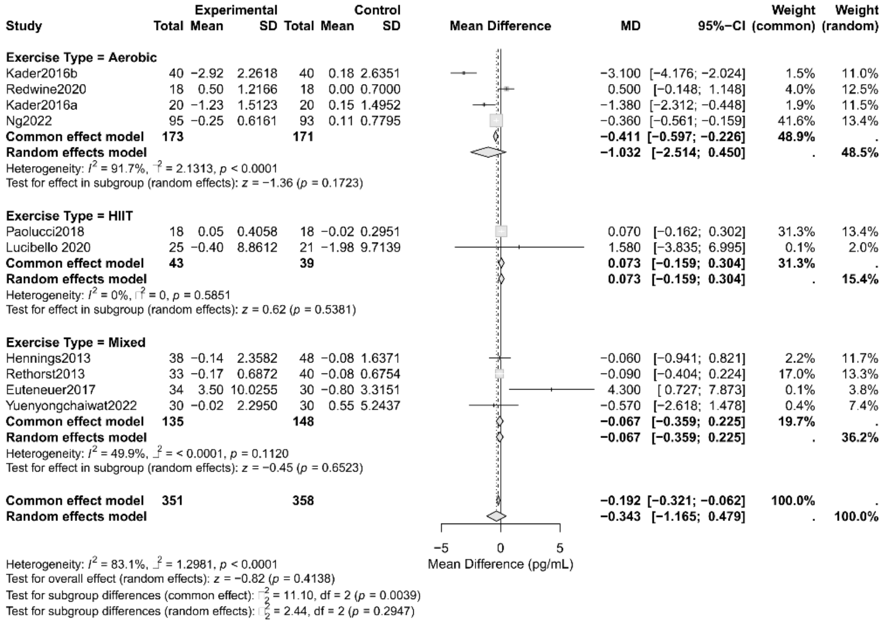

Study
Experimental Total Mean SD
Control Total Mean SD
Mean Difference
MD 95%−CI
Weight (common)
Weight (random)
Exercise Type = Aerobic
Kader2016b 40 −2.92 2.2618 40 0.18 2.6351 −3.100 [−4.176; −2.024] 1.5% 11.0%
Redwine2020 18 0.50 1.2166 18 0.00 0.7000 0.500 [−0.148; 1.148] 4.0% 12.5%
Kader2016a 20 −1.23 1.5123 20 0.15 1.4952 −1.380 [−2.312; −0.448] 1.9% 11.5%
Ng2022 95 −0.25 0.6161 93 0.11 0.7795 −0.360 [−0.561; −0.159] 41.6% 13.4%
Common effect model 173 171 −0.411 [−0.597; −0.226] 48.9% .
Random effects model −1.032 [−2.514; 0.450] . 48.5%
Heterogeneity: I^2 = 91.7%, τ^2 = 2.1313, p < 0.0001
Test for effect in subgroup (random effects): z = −1.36 (p = 0.1723)
Exercise Type = HIIT
Paolucci2018 18 0.05 0.4058 18 −0.02 0.2951 0.070 [−0.162; 0.302] 31.3% 13.4%
Lucibello 2020 25 −0.40 8.8612 21 −1.98 9.7139 1.580 [−3.835; 6.995] 0.1% 2.0%
Common effect model 43 39 0.073 [−0.159; 0.304] 31.3% .
Random effects model 0.073 [−0.159; 0.304] . 15.4%
Heterogeneity: I^2 = 0%, τ^2 = 0, p = 0.5851
Test for effect in subgroup (random effects): z = 0.62 (p = 0.5381)
Exercise Type = Mixed
Hennings2013 38 −0.14 2.3582 48 −0.08 1.6371 −0.060 [−0.941; 0.821] 2.2% 11.7%
Rethorst2013 33 −0.17 0.6872 40 −0.08 0.6754 −0.090 [−0.404; 0.224] 17.0% 13.3%
Euteneuer2017 34 3.50 10.0255 30 −0.80 3.3151 4.300 [0.727; 7.873] 0.1% 3.8%
Yuenyongchaiwat2022 30 −0.02 2.2950 30 0.55 5.2437 −0.570 [−2.618; 1.478] 0.4% 7.4%
Common effect model 135 148 −0.067 [−0.359; 0.225] 19.7% .
Random effects model −0.067 [−0.359; 0.225] . 36.2%
Heterogeneity: I^2 = 49.9%, τ^2 = < 0.0001, p = 0.1120
Test for effect in subgroup (random effects): z = −0.45 (p = 0.6523)
Common effect model 351 358 −0.192 [−0.321; −0.062] 100.0% .
Random effects model −0.343 [−1.165; 0.479] . 100.0%
−5
0
5
Mean Difference (pg/mL)
Heterogeneity: I^2 = 83.1%, τ^2 = 1.2981, p < 0.0001
Test for overall effect (random effects): z = −0.82 (p = 0.4138)
Test for subgroup differences (common effect): χ^2_2 = 11.10, df = 2 (p = 0.0039)
Test for subgroup differences (random effects): χ^2_2 = 2.44, df = 2 (p = 0.2947)

e

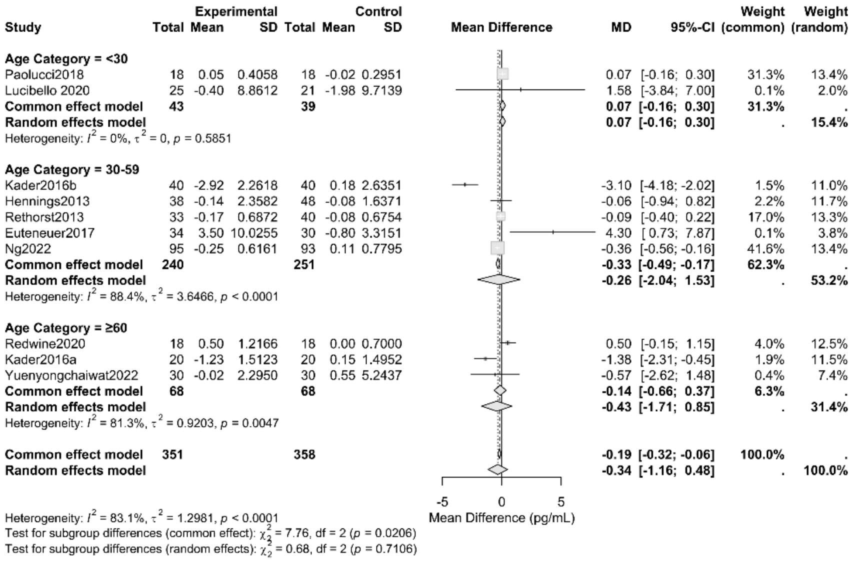

Experimental Control
Study Total Mean SD Total Mean SD Mean Difference MD 95%-CI Weight (common) Weight (random)
Age Category = <30
Paolucci2018 18 0.05 0.4058 18 -0.02 0.2951 0.07 [-0.16; 0.30] 31.3% 13.4%
Lucibello 2020 25 -0.40 8.8612 21 -1.98 9.7139 1.58 [-3.84; 7.00] 0.1% 2.0%
Common effect model 43 39 0.07 [-0.16; 0.30] 31.3% .
Random effects model 0.07 [-0.16; 0.30] . 15.4%
Heterogeneity: I^2 = 0%, τ^2 = 0, p = 0.5851
Age Category = 30-59
Kader2016b 40 -2.92 2.2618 40 0.18 2.6351 -3.10 [-4.18; -2.02] 1.5% 11.0%
Hennings2013 38 -0.14 2.3582 48 -0.08 1.6371 -0.06 [-0.94; 0.82] 2.2% 11.7%
Rethorst2013 33 -0.17 0.6872 40 -0.08 0.6754 -0.09 [-0.40; 0.22] 17.0% 13.3%
Euteneuer2017 34 3.50 10.0255 30 -0.80 3.3151 4.30 [0.73; 7.87] 0.1% 3.8%
Ng2022 95 -0.25 0.6161 93 0.11 0.7795 -0.36 [-0.56; -0.16] 41.6% 13.4%
Common effect model 240 251 -0.33 [-0.49; -0.17] 62.3% .
Random effects model -0.26 [-2.04; 1.53] . 53.2%
Heterogeneity: I^2 = 88.4%, τ^2 = 3.6466, p < 0.0001
Age Category = ≥60
Redwine2020 18 0.50 1.2166 18 0.00 0.7000 0.50 [-0.15; 1.15] 4.0% 12.5%
Kader2016a 20 -1.23 1.5123 20 0.15 1.4952 -1.38 [-2.31; -0.45] 1.9% 11.5%
Yuenyongchaiwat2022 30 -0.02 2.2950 30 0.55 5.2437 -0.57 [-2.62; 1.48] 0.4% 7.4%
Common effect model 68 68 -0.14 [-0.66; 0.37] 6.3% .
Random effects model -0.43 [-1.71; 0.85] . 31.4%
Heterogeneity: I^2 = 81.3%, τ^2 = 0.9203, p = 0.0047
Common effect model 351 358 -0.19 [-0.32; -0.06] 100.0% .
Random effects model -0.34 [-1.16; 0.48] . 100.0%
-5 0 5
Mean Difference (pg/mL)
Heterogeneity: I^2 = 83.1%, τ^2 = 1.2981, p < 0.0001
Test for subgroup differences (common effect): χ^2_2 = 7.76, df = 2 (p = 0.0206)
Test for subgroup differences (random effects): χ^2_2 = 0.68, df = 2 (p = 0.7106)

F

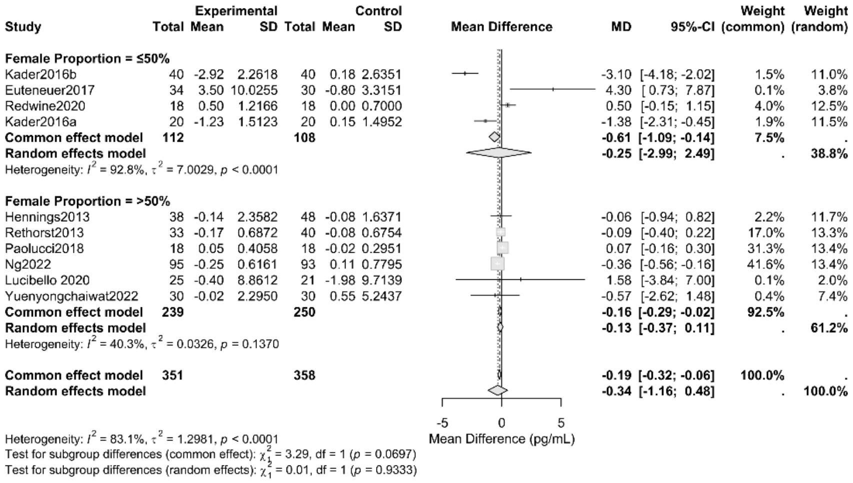

Experimental Control
Study Total Mean SD Total Mean SD Mean Difference MD 95%-CI Weight (common) Weight (random)
Female Proportion = ≤50%
Kader2016b 40 -2.92 2.2618 40 0.18 2.6351 -3.10 [-4.18; -2.02] 1.5% 11.0%
Euteneuer2017 34 3.50 10.0255 30 -0.80 3.3151 4.30 [0.73; 7.87] 0.1% 3.8%
Redwine2020 18 0.50 1.2166 18 0.00 0.7000 0.50 [-0.15; 1.15] 4.0% 12.5%
Kader2016a 20 -1.23 1.5123 20 0.15 1.4952 -1.38 [-2.31; -0.45] 1.9% 11.5%
Common effect model 112 108 -0.61 [-1.09; -0.14] 7.5% .
Random effects model -0.25 [-2.99; 2.49] . 38.8%
Heterogeneity: I^2 = 92.8%, τ^2 = 7.0029, p < 0.0001
Female Proportion = >50%
Hennings2013 38 -0.14 2.3582 48 -0.08 1.6371 -0.06 [-0.94; 0.82] 2.2% 11.7%
Rethorst2013 33 -0.17 0.6872 40 -0.08 0.6754 -0.09 [-0.40; 0.22] 17.0% 13.3%
Paolucci2018 18 0.05 0.4058 18 -0.02 0.2951 0.07 [-0.16; 0.30] 31.3% 13.4%
Ng2022 95 -0.25 0.6161 93 0.11 0.7795 -0.36 [-0.56; -0.16] 41.6% 13.4%
Lucibello 2020 25 -0.40 8.8612 21 -1.98 9.7139 1.58 [-3.84; 7.00] 0.1% 2.0%
Yuenyongchaiwat2022 30 -0.02 2.2950 30 0.55 5.2437 -0.57 [-2.62; 1.48] 0.4% 7.4%
Common effect model 239 250 -0.16 [-0.29; -0.02] 92.5% .
Random effects model -0.13 [-0.37; 0.11] . 61.2%
Heterogeneity: I^2 = 40.3%, τ^2 = 0.0326, p = 0.1370
Common effect model 351 358 -0.19 [-0.32; -0.06] 100.0% .
Random effects model -0.34 [-1.16; 0.48] . 100.0%
-5 0 5
Mean Difference (pg/mL)
Heterogeneity: I^2 = 83.1%, τ^2 = 1.2981, p < 0.0001
Test for subgroup differences (common effect): χ^2_1 = 3.29, df = 1 (p = 0.0697)
Test for subgroup differences (random effects): χ^2_1 = 0.01, df = 1 (p = 0.9333)

g

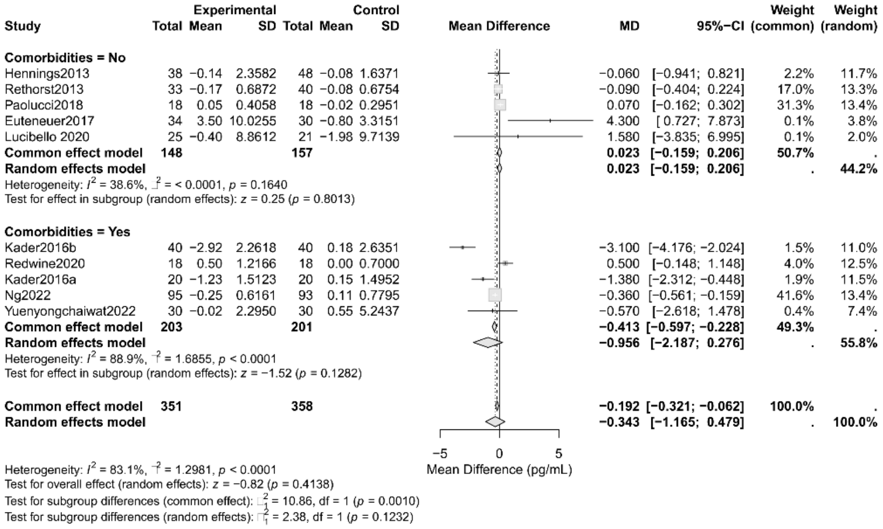

h

Figure S4: Subgroup Analysis of Exercise-Induced IL-6 Level Changes by Intervention Parameters and Participant Characteristics. (**a**) Forest plot of the mean difference in IL-6 levels at different exercise intensities; (**b**) Forest plot of the mean difference in IL-6 levels at different exercise length; (**c**) Forest plot of the mean difference in IL-6 levels at different exercise frequency; (**d**) Forest plot of the mean difference in IL-6 levels at different depression severities; **(e)** Forest plot of the mean difference in IL-6 levels at different exercise types; (**f**) Forest plot of the mean difference in IL-6 levels at different age ranges; (**g**) Forest plot of the mean difference in IL-6 levels at different proportion of females; **(h)** Forest plot of the mean difference in IL-6 levels in the presence or absence of comorbidities. The references in Figure S4 are Ng2022 [51], Abd El-Kader2016a[69], Vučić Lovrenčić 2015[76], Redwine2020[72], Lavretsky 2011[71], Euteneuer 2017[50], Paolucci 2018[49], Hennings2013[77], Abd El-Kader2016b[70], Yuenyongchaiwat2022[74], Lucibello2020[75], Rethorst2013[52], Imbo-den2021[73].

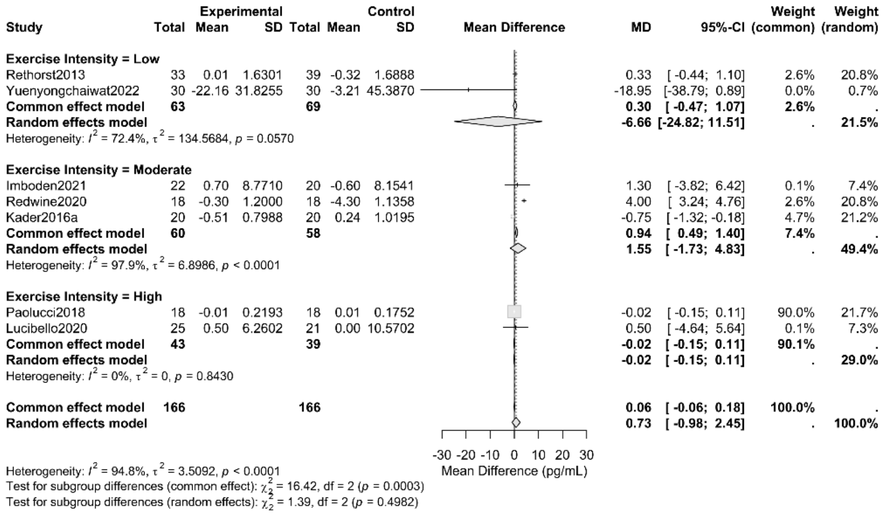
Study Total Experimental Mean SD Total Control Mean SD Mean Difference MD 95%-CI Weight (common) Weight (random)
Exercise Intensity = Low
Rethorst2013 33 0.01 1.6301 39 -0.32 1.6888 0.33 [-0.44; 1.10] 2.6% 20.8%
Yuenyongchaiwat2022 30 -22.16 31.8255 30 -3.21 45.3870 -18.95 [-38.79; 0.89] 0.0% 0.7%
Common effect model 63 69 0.30 [-0.47; 1.07] 2.6% .
Random effects model -6.66 [-24.82; 11.51] . 21.5%
Heterogeneity: I^2 = 72.4%, τ^2 = 134.5684, p = 0.0570
Exercise Intensity = Moderate
Imboden2021 22 0.70 8.7710 20 -0.60 8.1541 1.30 [-3.82; 6.42] 0.1% 7.4%
Redwine2020 18 -0.30 1.2000 18 -4.30 1.1358 4.00 [3.24; 4.76] 2.6% 20.8%
Kader2016a 20 -0.51 0.7988 20 0.24 1.0195 -0.75 [-1.32; -0.18] 4.7% 21.2%
Common effect model 60 58 0.94 [0.49; 1.40] 7.4% .
Random effects model 1.55 [-1.73; 4.83] . 49.4%
Heterogeneity: I^2 = 97.9%, τ^2 = 6.8986, p < 0.0001
Exercise Intensity = High
Paolucci2018 18 -0.01 0.2193 18 0.01 0.1752 -0.02 [-0.15; 0.11] 90.0% 21.7%
Lucibello2020 25 0.50 6.2602 21 0.00 10.5702 0.50 [-4.64; 5.64] 0.1% 7.3%
Common effect model 43 39 -0.02 [-0.15; 0.11] 90.1% .
Random effects model -0.02 [-0.15; 0.11] . 29.0%
Heterogeneity: I^2 = 0%, τ^2 = 0, p = 0.8430
Common effect model 166 166 0.06 [-0.06; 0.18] 100.0% .
Random effects model 0.73 [-0.98; 2.45] . 100.0%
-30 -20 -10 0 10 20 30
Mean Difference (pg/mL)
Heterogeneity: I^2 = 94.8%, τ^2 = 3.5092, p < 0.0001
Test for subgroup differences (common effect): χ^2_2 = 16.42, df = 2 (p = 0.0003)
Test for subgroup differences (random effects): χ^2_2 = 1.39, df = 2 (p = 0.4982)

a

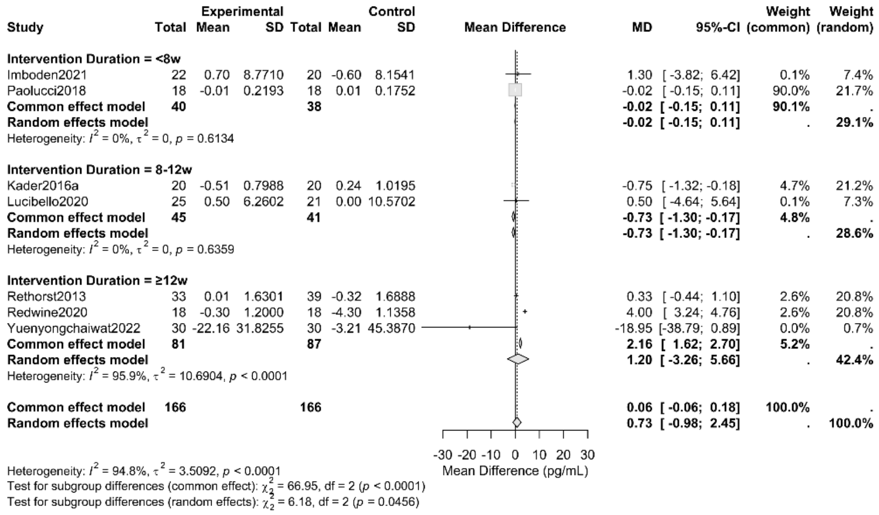
Study Total Experimental Mean SD Total Control Mean SD Mean Difference MD 95%-CI Weight (common) Weight (random)
Intervention Duration = <8w
Imboden2021 22 0.70 8.7710 20 -0.60 8.1541 1.30 [-3.82; 6.42] 0.1% 7.4%
Paolucci2018 18 -0.01 0.2193 18 0.01 0.1752 -0.02 [-0.15; 0.11] 90.0% 21.7%
Common effect model 40 38 -0.02 [-0.15; 0.11] 90.1% .
Random effects model -0.02 [-0.15; 0.11] . 29.1%
Heterogeneity: I^2 = 0%, τ^2 = 0, p = 0.6134
Intervention Duration = 8-12w
Kader2016a 20 -0.51 0.7988 20 0.24 1.0195 -0.75 [-1.32; -0.18] 4.7% 21.2%
Lucibello2020 25 0.50 6.2602 21 0.00 10.5702 0.50 [-4.64; 5.64] 0.1% 7.3%
Common effect model 45 41 -0.73 [-1.30; -0.17] 4.8% .
Random effects model -0.73 [-1.30; -0.17] . 28.6%
Heterogeneity: I^2 = 0%, τ^2 = 0, p = 0.6359
Intervention Duration = ≥12w
Rethorst2013 33 0.01 1.6301 39 -0.32 1.6888 0.33 [-0.44; 1.10] 2.6% 20.8%
Redwine2020 18 -0.30 1.2000 18 -4.30 1.1358 4.00 [3.24; 4.76] 2.6% 20.8%
Yuenyongchaiwat2022 30 -22.16 31.8255 30 -3.21 45.3870 -18.95 [-38.79; 0.89] 0.0% 0.7%
Common effect model 81 87 2.16 [1.62; 2.70] 5.2% .
Random effects model 1.20 [-3.26; 5.66] . 42.4%
Heterogeneity: I^2 = 95.9%, τ^2 = 10.6904, p < 0.0001
Common effect model 166 166 0.06 [-0.06; 0.18] 100.0% .
Random effects model 0.73 [-0.98; 2.45] . 100.0%
-30 -20 -10 0 10 20 30
Mean Difference (pg/mL)
Heterogeneity: I^2 = 94.8%, τ^2 = 3.5092, p < 0.0001
Test for subgroup differences (common effect): χ^2_2 = 66.95, df = 2 (p < 0.0001)
Test for subgroup differences (random effects): χ^2_2 = 6.18, df = 2 (p = 0.0456)

b

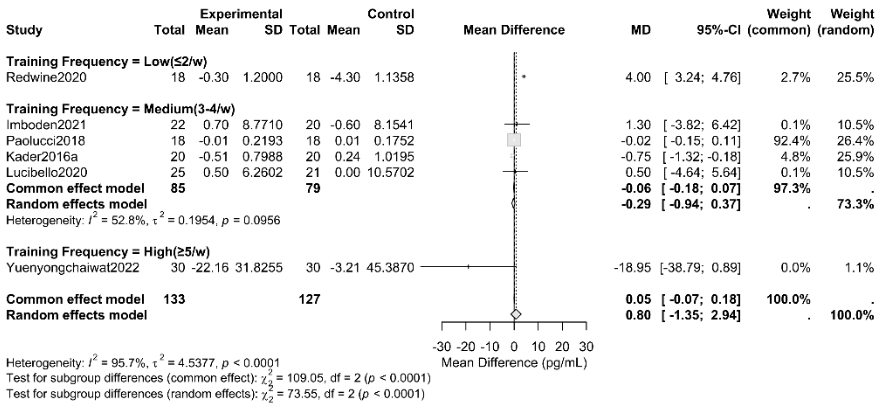
Study Total Experimental Mean SD Total Control Mean SD Mean Difference MD 95%-CI Weight (common) Weight (random)
Training Frequency = Low(≤2/w)
Redwine2020 18 -0.30 1.2000 18 -4.30 1.1358 4.00 [3.24; 4.76] 2.7% 25.5%
Training Frequency = Medium(3-4/w)
Imboden2021 22 0.70 8.7710 20 -0.60 8.1541 1.30 [-3.82; 6.42] 0.1% 10.5%
Paolucci2018 18 -0.01 0.2193 18 0.01 0.1752 -0.02 [-0.15; 0.11] 92.4% 26.4%
Kader2016a 20 -0.51 0.7988 20 0.24 1.0195 -0.75 [-1.32; -0.18] 4.8% 25.9%
Lucibello2020 25 0.50 6.2602 21 0.00 10.5702 0.50 [-4.64; 5.64] 0.1% 10.5%
Common effect model 85 79 -0.06 [-0.18; 0.07] 97.3% .
Random effects model -0.29 [-0.94; 0.37] . 73.3%
Heterogeneity: I^2 = 52.8%, τ^2 = 0.1954, p = 0.0956
Training Frequency = High(≥5/w)
Yuenyongchaiwat2022 30 -22.16 31.8255 30 -3.21 45.3870 -18.95 [-38.79; 0.89] 0.0% 1.1%
Common effect model 133 127 0.05 [-0.07; 0.18] 100.0% .
Random effects model 0.80 [-1.35; 2.94] . 100.0%
-30 -20 -10 0 10 20 30
Mean Difference (pg/mL)
Heterogeneity: I^2 = 95.7%, τ^2 = 4.5377, p < 0.0001
Test for subgroup differences (common effect): χ^2_2 = 109.05, df = 2 (p < 0.0001)
Test for subgroup differences (random effects): χ^2_2 = 73.55, df = 2 (p < 0.0001)

c

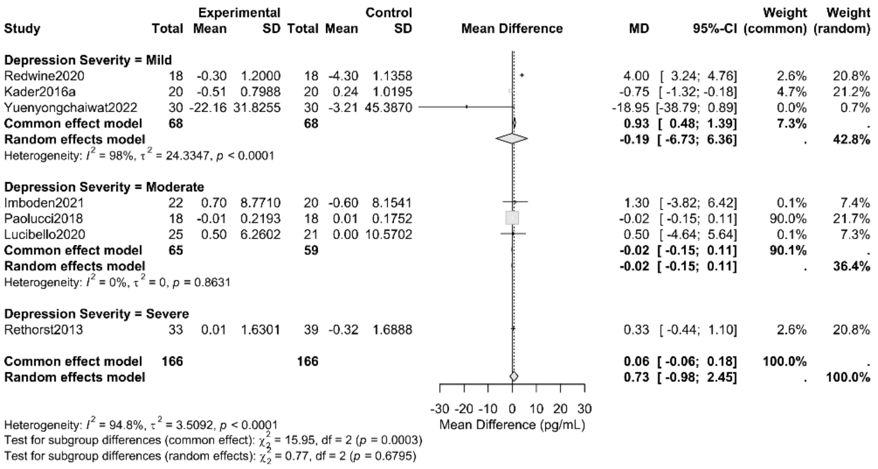
Study
Experimental Total Mean SD
Control Total Mean SD
Mean Difference
MD 95%-CI
Weight (common)
Weight (random)
Depression Severity = Mild
Redwine2020 18 -0.30 1.2000 18 -4.30 1.1358 4.00 [3.24; 4.76] 2.6% 20.8%
Kader2016a 20 -0.51 0.7988 20 0.24 1.0195 -0.75 [-1.32; -0.18] 4.7% 21.2%
Yuenyongchaiwat2022 30 -22.16 31.8255 30 -3.21 45.3870 -18.95 [-38.79; 0.89] 0.0% 0.7%
Common effect model 68 68 0.93 [0.48; 1.39] 7.3% .
Random effects model -0.19 [-6.73; 6.36] . 42.8%
Heterogeneity: I^2 = 98%, τ^2 = 24.3347, p < 0.0001
Depression Severity = Moderate
Imboden2021 22 0.70 8.7710 20 -0.60 8.1541 1.30 [-3.82; 6.42] 0.1% 7.4%
Paolucci2018 18 -0.01 0.2193 18 0.01 0.1752 -0.02 [-0.15; 0.11] 90.0% 21.7%
Lucibello2020 25 0.50 6.2602 21 0.00 10.5702 0.50 [-4.64; 5.64] 0.1% 7.3%
Common effect model 65 59 -0.02 [-0.15; 0.11] 90.1% .
Random effects model -0.02 [-0.15; 0.11] . 36.4%
Heterogeneity: I^2 = 0%, τ^2 = 0, p = 0.8631
Depression Severity = Severe
Rethorst2013 33 0.01 1.6301 39 -0.32 1.6888 0.33 [-0.44; 1.10] 2.6% 20.8%
Common effect model 166 166 0.06 [-0.06; 0.18] 100.0% .
Random effects model 0.73 [-0.98; 2.45] . 100.0%
-30 -20 -10 0 10 20 30
Mean Difference (pg/mL)
Heterogeneity: I^2 = 94.8%, τ^2 = 3.5092, p < 0.0001
Test for subgroup differences (common effect): χ^2_2 = 15.95, df = 2 (p = 0.0003)
Test for subgroup differences (random effects): χ^2_2 = 0.77, df = 2 (p = 0.6795)

d

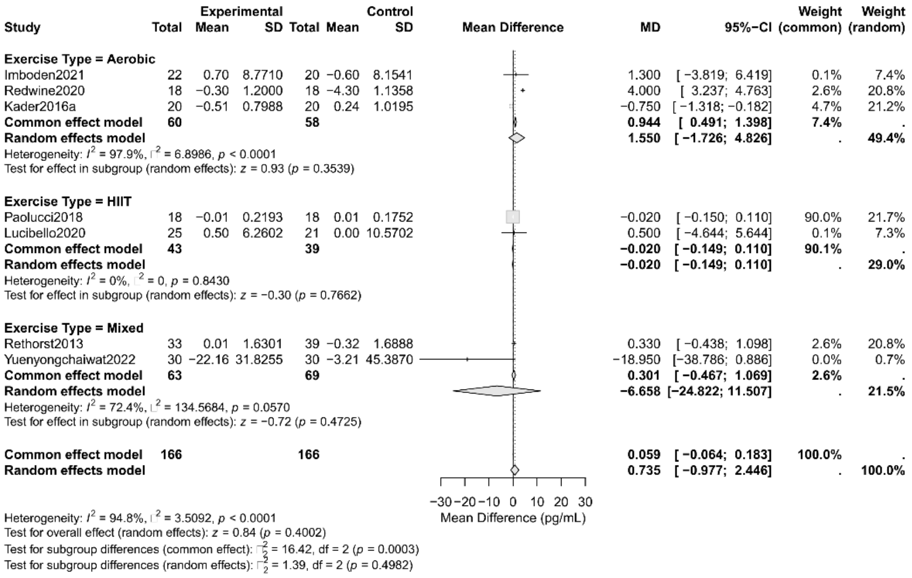
Study
Experimental Total Mean SD
Control Total Mean SD
Mean Difference
MD 95%−CI
Weight (common)
Weight (random)
Exercise Type = Aerobic
Imboden2021 22 0.70 8.7710 20 −0.60 8.1541 1.300 [−3.819; 6.419] 0.1% 7.4%
Redwine2020 18 −0.30 1.2000 18 −4.30 1.1358 4.000 [3.237; 4.763] 2.6% 20.8%
Kader2016a 20 −0.51 0.7988 20 0.24 1.0195 −0.750 [−1.318; −0.182] 4.7% 21.2%
Common effect model 60 58 0.944 [0.491; 1.398] 7.4% .
Random effects model 1.550 [−1.726; 4.826] . 49.4%
Heterogeneity: I^2 = 97.9%, τ^2 = 6.8986, p < 0.0001
Test for effect in subgroup (random effects): z = 0.93 (p = 0.3539)
Exercise Type = HIIT
Paolucci2018 18 −0.01 0.2193 18 0.01 0.1752 −0.020 [−0.150; 0.110] 90.0% 21.7%
Lucibello2020 25 0.50 6.2602 21 0.00 10.5702 0.500 [−4.644; 5.644] 0.1% 7.3%
Common effect model 43 39 −0.020 [−0.149; 0.110] 90.1% .
Random effects model −0.020 [−0.149; 0.110] . 29.0%
Heterogeneity: I^2 = 0%, τ^2 = 0, p = 0.8430
Test for effect in subgroup (random effects): z = −0.30 (p = 0.7662)
Exercise Type = Mixed
Rethorst2013 33 0.01 1.6301 39 −0.32 1.6888 0.330 [−0.438; 1.098] 2.6% 20.8%
Yuenyongchaiwat2022 30 −22.16 31.8255 30 −3.21 45.3870 −18.950 [−38.786; 0.886] 0.0% 0.7%
Common effect model 63 69 0.301 [−0.467; 1.069] 2.6% .
Random effects model −6.658 [−24.822; 11.507] . 21.5%
Heterogeneity: I^2 = 72.4%, τ^2 = 134.5684, p = 0.0570
Test for effect in subgroup (random effects): z = −0.72 (p = 0.4725)
Common effect model 166 166 0.059 [−0.064; 0.183] 100.0% .
Random effects model 0.735 [−0.977; 2.446] . 100.0%
−30 −20 −10 0 10 20 30
Mean Difference (pg/mL)
Heterogeneity: I^2 = 94.8%, τ^2 = 3.5092, p < 0.0001
Test for overall effect (random effects): z = 0.84 (p = 0.4002)
Test for subgroup differences (common effect): χ^2_2 = 16.42, df = 2 (p = 0.0003)
Test for subgroup differences (random effects): χ^2_2 = 1.39, df = 2 (p = 0.4982)

e

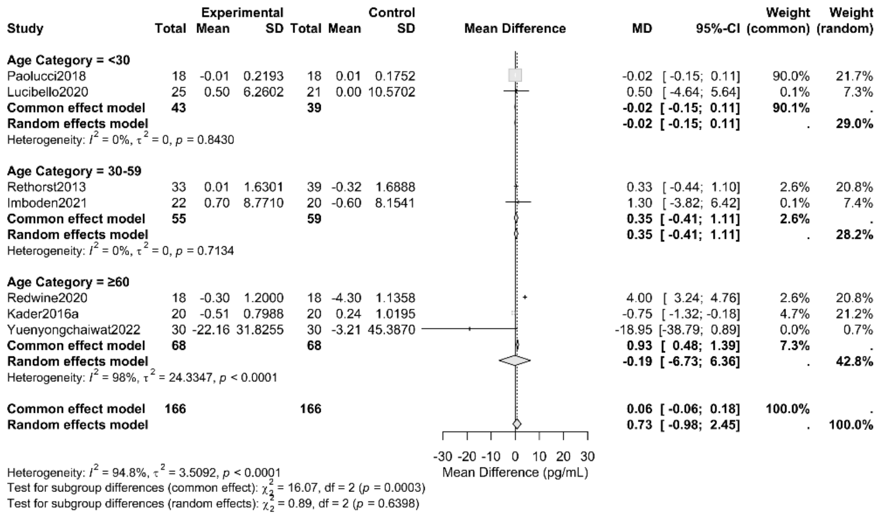
Study | Experimental Total Mean SD | Control Total Mean SD | Mean Difference | MD 95%-CI | Weight (common) | Weight (random)
Age Category = <30
Paolucci2018 18 -0.01 0.2193 18 0.01 0.1752 -0.02 [-0.15; 0.11] 90.0% 21.7%
Lucibello2020 25 0.50 6.2602 21 0.00 10.5702 0.50 [-4.64; 5.64] 0.1% 7.3%
Common effect model 43 39 -0.02 [-0.15; 0.11] 90.1% .
Random effects model -0.02 [-0.15; 0.11] . 29.0%
Heterogeneity: I^2 = 0%, τ^2 = 0, p = 0.8430
Age Category = 30-59
Rethorst2013 33 0.01 1.6301 39 -0.32 1.6888 0.33 [-0.44; 1.10] 2.6% 20.8%
Imboden2021 22 0.70 8.7710 20 -0.60 8.1541 1.30 [-3.82; 6.42] 0.1% 7.4%
Common effect model 55 59 0.35 [-0.41; 1.11] 2.6% .
Random effects model 0.35 [-0.41; 1.11] . 28.2%
Heterogeneity: I^2 = 0%, τ^2 = 0, p = 0.7134
Age Category = ≥60
Redwine2020 18 -0.30 1.2000 18 -4.30 1.1358 4.00 [3.24; 4.76] 2.6% 20.8%
Kader2016a 20 -0.51 0.7988 20 0.24 1.0195 -0.75 [-1.32; -0.18] 4.7% 21.2%
Yuenyongchaiwat2022 30 -22.16 31.8255 30 -3.21 45.3870 -18.95 [-38.79; 0.89] 0.0% 0.7%
Common effect model 68 68 0.93 [0.48; 1.39] 7.3% .
Random effects model -0.19 [-6.73; 6.36] . 42.8%
Heterogeneity: I^2 = 98%, τ^2 = 24.3347, p < 0.0001
Common effect model 166 166 0.06 [-0.06; 0.18] 100.0% .
Random effects model 0.73 [-0.98; 2.45] . 100.0%
-30 -20 -10 0 10 20 30
Mean Difference (pg/mL)
Heterogeneity: I^2 = 94.8%, τ^2 = 3.5092, p < 0.0001
Test for subgroup differences (common effect): χ^2_2 = 16.07, df = 2 (p = 0.0003)
Test for subgroup differences (random effects): χ^2_2 = 0.89, df = 2 (p = 0.6398)

f

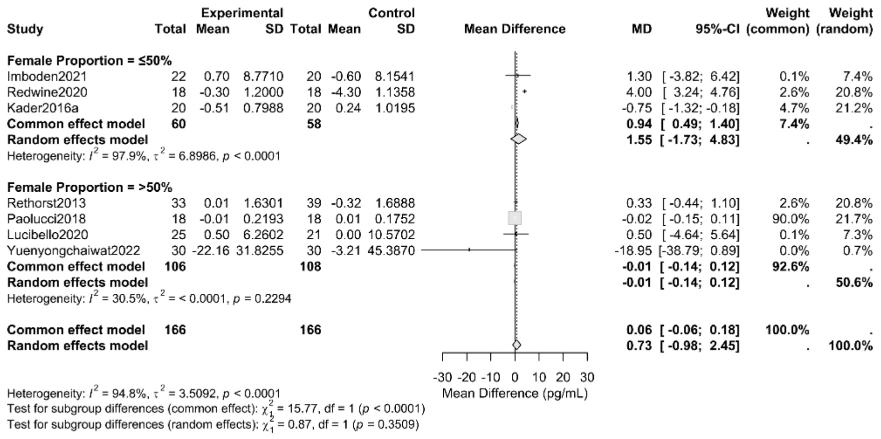
Study | Experimental Total Mean SD | Control Total Mean SD | Mean Difference | MD 95%-CI | Weight (common) | Weight (random)
Female Proportion = ≤50%
Imboden2021 22 0.70 8.7710 20 -0.60 8.1541 1.30 [-3.82; 6.42] 0.1% 7.4%
Redwine2020 18 -0.30 1.2000 18 -4.30 1.1358 4.00 [3.24; 4.76] 2.6% 20.8%
Kader2016a 20 -0.51 0.7988 20 0.24 1.0195 -0.75 [-1.32; -0.18] 4.7% 21.2%
Common effect model 60 58 0.94 [0.49; 1.40] 7.4% .
Random effects model 1.55 [-1.73; 4.83] . 49.4%
Heterogeneity: I^2 = 97.9%, τ^2 = 6.8986, p < 0.0001
Female Proportion = >50%
Rethorst2013 33 0.01 1.6301 39 -0.32 1.6888 0.33 [-0.44; 1.10] 2.6% 20.8%
Paolucci2018 18 -0.01 0.2193 18 0.01 0.1752 -0.02 [-0.15; 0.11] 90.0% 21.7%
Lucibello2020 25 0.50 6.2602 21 0.00 10.5702 0.50 [-4.64; 5.64] 0.1% 7.3%
Yuenyongchaiwat2022 30 -22.16 31.8255 30 -3.21 45.3870 -18.95 [-38.79; 0.89] 0.0% 0.7%
Common effect model 106 108 -0.01 [-0.14; 0.12] 92.6% .
Random effects model -0.01 [-0.14; 0.12] . 50.6%
Heterogeneity: I^2 = 30.5%, τ^2 = < 0.0001, p = 0.2294
Common effect model 166 166 0.06 [-0.06; 0.18] 100.0% .
Random effects model 0.73 [-0.98; 2.45] . 100.0%
-30 -20 -10 0 10 20 30
Mean Difference (pg/mL)
Heterogeneity: I^2 = 94.8%, τ^2 = 3.5092, p < 0.0001
Test for subgroup differences (common effect): χ^2_1 = 15.77, df = 1 (p < 0.0001)
Test for subgroup differences (random effects): χ^2_1 = 0.87, df = 1 (p = 0.3509)

g

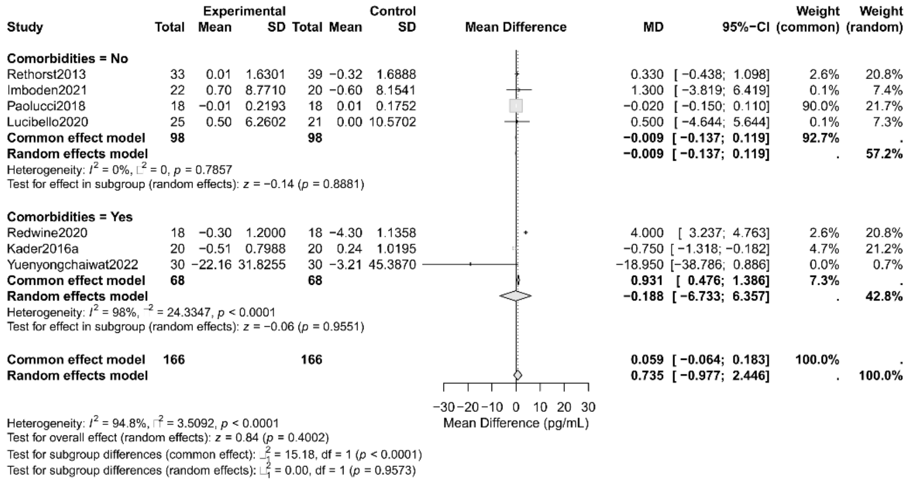
Study | Experimental Total Mean SD | Control Total Mean SD | Mean Difference | MD 95%-CI | Weight (common) | Weight (random)
Comorbidities = No
Rethorst2013 33 0.01 1.6301 39 -0.32 1.6888 0.330 [-0.438; 1.098] 2.6% 20.8%
Imboden2021 22 0.70 8.7710 20 −0.60 8.1541 1.300 [−3.819; 6.419] 0.1% 7.4%
Paolucci2018 18 −0.01 0.2193 18 0.01 0.1752 −0.020 [−0.150; 0.110] 90.0% 21.7%
Lucibello2020 25 0.50 6.2602 21 0.00 10.5702 0.500 [−4.644; 5.644] 0.1% 7.3%
Common effect model 98 98 −0.009 [−0.137; 0.119] 92.7% .
Random effects model −0.009 [−0.137; 0.119] . 57.2%
Heterogeneity: I^2 = 0%, = 0, p = 0.7857
Test for effect in subgroup (random effects): z = −0.14 (p = 0.8881)
Comorbidities = Yes
Redwine2020 18 −0.30 1.2000 18 −4.30 1.1358 4.000 [3.237; 4.763] 2.6% 20.8%
Kader2016a 20 −0.51 0.7988 20 0.24 1.0195 −0.750 [−1.318; −0.182] 4.7% 21.2%
Yuenyongchaiwat2022 30 −22.16 31.8255 30 −3.21 45.3870 −18.950 [−38.786; 0.886] 0.0% 0.7%
Common effect model 68 68 0.931 [0.476; 1.386] 7.3% .
Random effects model −0.188 [−6.733; 6.357] . 42.8%
Heterogeneity: I^2 = 98%, = 24.3347, p < 0.0001
Test for effect in subgroup (random effects): z = −0.06 (p = 0.9551)
Common effect model 166 166 0.059 [−0.064; 0.183] 100.0% .
Random effects model 0.735 [−0.977; 2.446] . 100.0%
−30 −20 −10 0 10 20 30
Mean Difference (pg/mL)
Heterogeneity: I^2 = 94.8%, = 3.5092, p < 0.0001
Test for overall effect (random effects): z = 0.84 (p = 0.4002)
Test for subgroup differences (common effect): = 15.18, df = 1 (p < 0.0001)
Test for subgroup differences (random effects): = 0.00, df = 1 (p = 0.9573)

h

Figure S5: Subgroup Analysis of Exercise-Induced TNF-α Level Changes by Intervention Parameters and Participant Characteristics. (**a**) Forest plot of the mean difference in TNF-α levels at different exercise intensities; (**b**) Forest plot of the mean difference in TNF-α levels at different exercise length; (**c**) Forest plot of the mean difference in TNF-α levels at different exercise frequency; (**d**) Forest plot of the mean difference in TNF-α levels at different depression severities; (**e**) Forest plot of the mean difference in TNF-α levels at different exercise types; (**f**) Forest plot of the mean difference in TNF-α levels at different age ranges; (**g**) Forest plot of the mean difference in TNF-α levels at different proportion of females; **(h)** Forest plot of the mean difference in TNF-α levels in the presence or absence of comorbidities. The references in Figure S5 are Ng2022 [51], Abd El-Kader2016a[69], Vučić Lovrenčić 2015[76], Redwine2020[72], Lavretsky 2011[71], Euteneuer 2017[50], Paolucci 2018[49], Hennings2013[77], Abd El-Kader2016b[70], Yuenyongchaiwat2022[74], Lucibello2020[75], Rethorst2013[52], Imbo-den2021[73].

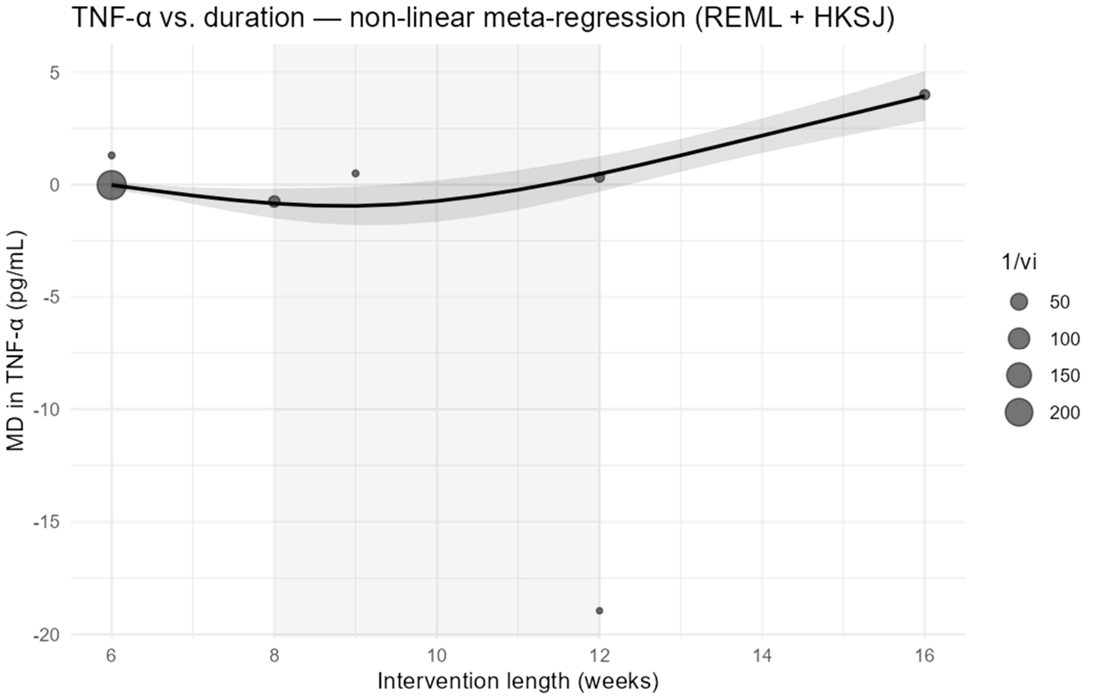

Figure S6. Non-Linear Relationship Between Exercise Duration and Change in TNF-α Levels.
