# Supplementary material for: Effect of Different Exercise Modalities on Inflammatory Markers in Individuals with Depressive Disorder: A Systematic Review and Meta-Analysis
Source: Life (Basel). 2025 Sep 16;15(9):1452. doi: 10.3390/life15091452 (PMC12472113; doi:10.3390/life15091452)
Supplement: Supplementary file 1 [file life-15-01452-s001.zip › Supplementary File S2 (PRISMA_2020_checklist).pdf]

PRISMA 2020 Checklist

Section and Topic	Item #	Checklist item	Location where item is reported
**TITLE**			
Title	1	Effect of Different Exercise Modalities on Inflammatory Markers in Individuals with Depressive Disorder: A Systematic Re-view and Meta-Analysis	p.1
**ABSTRACT**			
Abstract	2	Background: One of the important mechanisms of depression is long-term high levels of inflammation. Exercise can effectively relieve depressive symptoms and has an-ti-inflammatory effect. This research methodically assesses how various exercise modalities influence peripheral inflammatory markers in individuals with depression, so that more personalized and precise exercise schemes can be provided for people with depression to improve the inflammation.<br>Methods: Randomized controlled trials on depressive disorder, exercise and inflammation published until May 2025 were screened in five databases. The Cochrane Risk of Bias tool (RoB2.0) was used to evaluate potential biases, with random effects meta-analyses gauging the impact of varying exercise regimens on peripheral inflammatory markers (CRP, IL-6 and TNF-α) involving exercise types, intensity, frequency and length. Meta-regression analyses ware employed to show the sources of heterogeneity and investigate potential moderator variables influencing the CRP, IL-6, and TNF-α. (PROSPERO CRD42024590612)<br>Results: 13 unique studies with 1004 participants were included. Overall, exercise training could alleviate depressive symptoms (SMD = -0.59, 95%CI: [-0.82, -0.35], I^2 = 61.7%, $p < 0.0001$), and subgroup analysis showed that exercise of lasting 8-12 weeks could reduce the level of TNF-α (MD = -0.73, 95%CI: [-0.17, -1.30], I2 = 0, $p < 0.05$). However, univariable regression analysis demonstrated a significant positive association between exercise period and TNF-α levels (β = 0.24, p = 0.039).<br>Conclusion: Exercise could alleviate depressive symptoms in individuals with depression. However, the influence of exercise length on TNF-α levels has conflicting conclusions across different analytical methods, which may be attributed to methodological variations and worth further investigation.	p.1 (Abstract)
**INTRODUCTION**			
Rationale	3	Antidepressants combined with physical exercise is an effective non-pharmacological intervention for individuals with depression to improve depressive symptoms and reduce peripheral proinflammatory factors. Despite these advances, critical gaps remain. Previous researches have failed to establish a clear quantitative dose-response relationship between specific exercise regimens (e.g., type, intensity, length and frequency) and the magnitude of reductions in peripheral inflammation markers, particularly in depressed populations, creating a significant gap in developing targeted exercise protocols for individuals with depressive disorders. To directly address this critical gap and provide actionable insights for clinical practice, this meta-analysis systematically quantifies the effects of various exercise intervention characteristics (defined by their specific parameters) on pro-inflammatory biomarkers in individuals with depression, aiming to elucidate potential dose-response relationships.	p.2-3, section1 (Introduction)
Objectives	4	This systematic review aims to answer: In adults with depressive disorders, compared to control/routine care, can exercise interventions reduce peripheral inflammatory markers (CRP, IL-6, TNF-α, etc.)? Additionally, it explores the moderating effects of exercise modality, intensity, frequency, and duration on outcomes.	p.3, section 1 (Introduction)
**METHODS**			
Eligibility criteria	5	Inclusion criteria<br>All studies incorporated into this meta-analysis had to satisfy specific PICOS criteria (Participants, Interventions, Comparisons, Outcomes, and Study Design). The inclusion requirements were as follows: (1) Participants had to be adults aged 18 or older, regardless of gender, with a formal diagnosis of depressive disorder based on the DSM-5™ Diagnostic Criteria reference guide or ICD classification. These diagnoses were confirmed either by licensed psychiatrists or through validated assessment tools; (2) The experimental group received regular exercise as a standalone treatment, de-fined as structed interventions meeting core parameters: intensity at light intensity (57%–63% HRmax), moderate intensity (64%–76% HRmax), high intensity (77%–95% HRmax); frequency of ⩾1 sessions/week; session duration type-specified as ⩾30 minutes for aerobic exercise, ⩾20 minutes for high-intensity interval training (including recovery), or full major muscle group	p.3-4, section 2.1 (Inclusion and exclusion criteria)

Section and Topic	Item #	Checklist item	Location where item is reported
		coverage (⩾2 sets/muscle) for resistance training; and total length of ⩾4 weeks. This definition integrates ACSM FITT-VP prescription principles. (3) The control group comprised individuals with a depression diagnosis who were not involved in any exercise-based intervention programs, including standard care, a waiting-list control, or placebo treatments, serve as the non-exercise control. These control conditions could be active (e.g., stretching, relaxation techniques) or passive (e.g., placebo). When investigating the effects of pan-population exercise or the antidepressant effect of different volume, the control group consisting of healthy subjects or DD individuals who received exercise intervention was acceptable; (4) The study focused chiefly on measuring depressive symptoms and inflammatory cytokine levels as its main endpoints. Additionally, it examined secondary outcomes, including how various forms of exercise, workout frequency, and session duration influenced both depression and anxiety symptoms, as well as inflammatory marker concentrations; (5) Only randomized controlled trials (RCTs) published in peer-reviewed journals or academic dissertations were considered; conference abstracts and presentations were excluded from the analysis.<br>Exclusion criteria<br>The meta-analysis excluded studies if they fell into any of the following criteria: (1) Clinical trials that were ongoing or had initiated physical activity programs for expectant or nursing mothers; (2) Trials that incorporated nutritional advice as a comparative intervention; (3) Research studies including individuals with a clinical diagnosis of anxiety or bipolar disorder; (4) Studies that were short on details about the specifics of exercise—like its frequency, intensity, duration, volume, and progression. (5) Animal studies, conference reports, review papers, editorials, and non-English language articles; (6) The experimental group used acute exercise intervention; (7) The data is incomplete and the data cannot be converted.	
Information sources	6	The protocol for this research was developed in advance and officially registered with PROSPERO (CRD42024590612). The review was conducted in accordance with the Preferred Reporting Items for Systematic Review and Meta-Analyses (PRISMA) statement. To gather pertinent studies published up to May 2025, searches were conducted across some databases including PubMed, the Cochrane Library, Embase, EBSCO, and Web of Science. The search incorporated keywords related to “depressive disorder,” “exercise,” and “inflammation” to ensure a comprehensive collection of relevant records.	p.4, section 2.2. (Search strategy)
Search strategy	7	Details of the search strategy and findings are available in Supplementary Table S1.<br>PubMed, The Cochrane Library: #1 ((((((((((((depressive disorders[MeSH Terms]) OR (Disorder, Depressive[Title/Abstract])) OR (Depressive Neuroses[Title/Abstract])) OR (Depressive Neurosis[Title/Abstract])) OR (Endogenous Depression[Title/Abstract])) OR (Endogenous Depressions[Title/Abstract])) OR (Depressive Syndrome[Title/Abstract])) OR (Depressive Syndromes[Title/Abstract])) OR (Neurotic Depression[Title/Abstract])) OR (Neurotic Depressions[Title/Abstract])) OR (Melancholia[Title/Abstract])) OR (Melancholias[Title/Abstract])) OR (Unipolar Depression[Title/Abstract])) OR (Unipolar Depressions[Title/Abstract])<br>#2 (((((((((((((exercise[MeSH Terms])) OR (Exercises[Title/Abstract])) OR (Physical Activity[Title/Abstract])) OR (Physical Activities[Title/Abstract])) OR (Physical Exercise[Title/Abstract])) OR (Physical Exercises[Title/Abstract])) OR (Acute Exercise[Title/Abstract])) OR (Acute Exercises[Title/Abstract])) OR (Isometric Exercises[Title/Abstract])) OR (Isometric Exercise[Title/Abstract])) OR (Aerobic Exercise[Title/Abstract])) OR (Aerobic Exercises[Title/Abstract])) OR (Exercise Training[Title/Abstract])) OR (Exercise Trainings[Title/Abstract])<br>#3 (((inflammation[MeSH Terms]) OR (Inflammations[Title/Abstract]))) OR (Innate Inflammatory Response[Title/Abstract])) OR (Innate Inflammatory Responses[Title/Abstract])<br>#1 AND #2 AND #3<br>Web of Science: #1 TS = (depressive disorders OR Disorder, Depressive OR Depressive Neuroses OR Depressive Neurosis OR Endogenous Depression OR Endogenous Depressions OR Depressive Syndrome OR Depressive Syndromes OR Neurotic Depression OR Neurotic Depressions OR Melancholia OR Melancholias OR Unipolar Depression OR Unipolar Depressions)<br>#2 TS = (exercise OR Exercises OR Physical Activity OR Physical Activities OR Physical Exercise OR Physical Exercises OR Acute Exercise OR Acute Exercises OR Isometric Exercises OR Isometric Exercise OR Aerobic Exercise OR Aerobic Exercises OR Exercise Training OR Exercise Trainings)	p4, section 2.2 (Search strategy) and Supplementary Table S1

Section and Topic	Item #	Checklist item	Location where item is reported
		#3 TS = (inflammation OR Inflammations OR Innate Inflammatory Response OR Innate Inflammatory Responses)<br>#1 AND #2 AND #3<br>Embase: #1 'depression'/exp OR 'depression' OR 'central depression'/exp OR 'central depression' OR 'clinical depression'/exp OR 'clinical depression' OR 'depressive disease'/exp OR 'depressive disease' OR 'depressive disorder'/exp OR 'depressive disorder' OR 'depressive episode'/exp OR 'depressive episode' OR 'depressive illness'/exp OR 'depressive illness' OR 'depressive personality disorder'/exp OR 'depressive personality disorder' OR 'depressive state'/exp OR 'depressive state' OR 'depressive symptom'/exp OR 'depressive symptom' OR 'depressive syndrome'/exp OR 'depressive syndrome' OR 'depressivity'/exp OR 'depressivity' OR 'mental depression'/exp OR 'mental depression' OR 'parental depression'/exp OR 'parental depression'<br>#2 'exercise'/exp OR 'exercise' OR 'biometric exercise'/exp OR 'biometric exercise' OR 'effort'/exp OR 'effort' OR 'exercise capacity'/exp OR 'exercise capacity' OR 'exercise performance'/exp OR 'exercise performance' OR 'exercise training'/exp OR 'exercise training' OR 'exertion'/exp OR 'exertion' OR 'fitness training'/exp OR 'fitness training' OR 'fitness workout'/exp OR 'fitness workout' OR 'physical conditioning, human'/exp OR 'physical conditioning, human' OR 'physical effort'/exp OR 'physical effort' OR 'physical exercise'/exp OR 'physical exercise' OR 'physical exertion'/exp OR 'physical exertion' OR 'physical work-out'/exp OR 'physical work-out' OR 'physical workout'/exp OR 'physical workout'<br>#3 'inflammation'/exp OR 'inflammation' OR 'acute inflammation'/exp OR 'acute inflammation' OR 'inflammation reaction'/exp OR 'inflammation reaction' OR 'inflammation response'/exp OR 'inflammation response' OR 'inflammatory condition'/exp OR 'inflammatory condition' OR 'inflammatory lesion'/exp OR 'inflammatory lesion' OR 'inflammatory process'/exp OR 'inflammatory process' OR 'inflammatory reaction'/exp OR 'inflammatory reaction' OR 'inflammatory response'/exp OR 'inflammatory response' OR 'inflammatory syndrome'/exp OR 'inflammatory syndrome' OR 'reaction, inflammation'/exp OR 'reaction, inflammation' OR 'response, inflammatory'/exp OR 'response, inflammatory'<br>#1 AND #2 AND #3<br>EBSCO: #1 depressive disorder or depressive symptoms or major depressive disorder<br>#2 exercise or physical fitness or physical activity or exercise therapy or physical therapy or walking<br>#3 inflammation or inflammatory or inflammation response<br>#1 AND #2 AND #3	
Selection process	8	Two reviewers screened records independently (JS and XJW); disagreements were resolved by a third reviewer (YL). The initial search yielded a total of 2833 potentially eligible reports. After duplicate entries were eliminated, 2228 titles and abstracts were carefully reviewed. In the next stage, 27 studies were chosen for the full-text reading; and of these, 14 studies were excluded, leaving 13 studies that fulfilled the inclusion criteria and were included in the present review.	p.6, section 3.1 (The process of study selection)
Data collection process	9	Two reviewers extracted data independently with cross-checking (JS and XJW); corresponding authors were contacted for missing data (YL). In each studies reviewed, pertinent details were extracted, including (1)study characteristics (title, author information, publication year); (2) participants' characteristics (sex, age and sample size); (3) clinical features (depression diagnostic criteria baseline depression severity using standard scales e.g., HAM-D/BDI);(4) exercise intervention parameters: exercise type (e.g., mixed exercise, traditional aerobic exercise, Mind-Body exercise and High-intensity interval exercise. These categories were selected because they represent classifications commonly used by health promotion organizations and are applicable to the exercise modalities adopted in the included studies. For this study, aerobic exercise was defined as any exercise intervention aimed at improving cardiovascular health. This includes walking, running, dancing, cycling, swimming, etc. Mind-Body exercise was categorized as exercise combining movement sequences, breath control, and attention regulation. Examples of Mind-Body exercise are Tai Chi, Pilates, and yoga. High-intensity interval training was defined as repeated bouts of relatively high-intensity exercise interspersed with easier recovery periods or rest). Mixed exercise is defined as a combination of at least two modalities; exercise intensity (light intensity: 57%–63% HRmax, moderate intensity: 64%–76% HRmax, high intensity: 77%–95% HRmax); exercise frequency; exercise length. (5) inflammatory cytokine levels (specific cytokines (e.g., CRP, IL-6, TNF-α), and pre/post-intervention mean ± SD or change values); (6) outcome measures (main outcome: the average difference in the alteration of biomarker concentrations,	p.4, section 2.4 (Data extraction)

PRISMA 2020 Checklist

Section and Topic	Item #	Checklist item	Location where item is reported
		from the pre-intervention to the post-intervention period, between those in the exercise group and the control group, following a sustained exercise program. The secondary outcome: measures of depressive symptoms. Studies that provided biomarker data of patients with depressive disorder were included, even if accurate information on depressive symptoms was not available).	
Data items	10a	Main outcome: the average difference in the alteration of biomarker concentrations, from the pre-intervention to the post-intervention period, between those in the exercise group and the control group, following a sustained exercise program. The secondary outcome: measures of depressive symptoms. Studies that provided biomarker data of patients with depressive disorder were included, even if accurate information on de-pressive symptoms was not available	p.4, section 2.4 (Data extraction)
	10b	(1) study characteristics (title, author information, publication year); (2) participants' characteristics (sex, age and sample size); (3) clinical features (depression diagnostic criteria baseline depression severity using standard scales e.g., HAM-D/BDI); (4) exercise intervention parameters: exercise type (e.g., mixed exercise, traditional aerobic exercise, Mind-Body exercise and High-intensity interval exercise. These categories were selected because they represent classifications commonly used by health promotion organizations and are applicable to the exercise modalities adopted in the included studies. For this study, aerobic exercise was defined as any exercise intervention aimed at improving cardiovascular health. This includes walking, running, dancing, cycling, swimming, etc. Mind-Body exercise was categorized as exercise combining movement sequences, breath control, and attention regulation. Examples of Mind-Body exercise are Tai Chi, Pilates, and yoga. High-intensity interval training was defined as repeated bouts of relatively high-intensity exercise interspersed with easier recovery periods or rest). Mixed exercise is defined as a combination of at least two modalities; exercise intensity (light intensity: 57%–63% HRmax, moderate intensity: 64%–76% HRmax, high intensity: 77%–95% HRmax); exercise frequency; exercise length. (5) inflammatory cytokine levels (specific cytokines (e.g., CRP, IL-6, TNF-α), and pre/post-intervention mean ± SD or change values);	p.4, section 2.4 (Data extraction)
Study risk of bias assessment	11	To evaluate the methodological rigor of the studies in our analysis, we employed the RoB 2.0 tool. Each study was classified into one of three categories: "low risk," "high risk," or "some concerns," across five critical domains: 1) randomization process; 2) deviations from the intended interventions; 3) missing outcome data; 4) measurement of the outcome; and 5) selection of the reported result. Two independent evaluators (JS and XJW) performed the assessments following the Cochrane RoB 2.0 guidelines, issuing individual ratings for every study. In cases where discrepancies arose, a third reviewer (YL) stepped in to mediate discussions until a unanimous decision was reached.	p.4, section 2.3 (Quality Assessment)
Effect measures	12	For depressive symptoms, outcomes were expressed as standardized mean differences (SMD) with 95% CIs, whereas mean differences (MD) were used for the three common inflammatory factors (CRP, IL-6, TNF-α). Biomarker units (e.g., IL-6 in pg/mL) and standard errors were harmonized to SDs.	p.5, section 2.5 (Data Synthesis and Analysis)
Synthesis methods	13a	For exploring the effects of specific exercise prescriptions on individuals with depression, we conducted a subgroup analysis. The selection of these analytical variables was based on the characteristics of the included literature, which suggests that the effects of exercise interventions may be influenced by the exercise parameters themselves (exercise type, intensity, frequency, length) and the baseline characteristics of the subjects (severity of depression, age).	p.5, section 2.5 (Data Synthesis and Analysis)
	13b	For multi-arm trials we combined relevant arms per Cochrane guidance. Units were harmonized (e.g., pg/mL) before pooling.	p.5, section 2.5 (Data Synthesis and Analysis)
	13c	We presented results with forest plots, funnel plots, summary tables and narrative synthesis where meta-analysis was not appropriate. Analyses were performed in R software (version 4.2.0), using the metafor, dplyr, ggplot2, and knitr packages.	p.5, section 2.5 (Data Synthesis and Analysis)
	13d	We calculate the pooled effect size within each subgroup and evaluate whether these factors significantly moderated the effect of exercise on inflammatory biomarkers by comparing the between-subgroup effect size estimates and the overlap of their confidence intervals, supplemented by tests of between-subgroup heterogeneity. When $I^2 < 50\%$, a fixed-effect model was applied, and a random-effects model was selected otherwise. Publication bias was assessed via funnel plots (⩾10 studies). All tests were two-tailed ($\alpha = 0.05$).	p.5, section 2.5 (Data Synthesis and Analysis)

Section and Topic	Item #	Checklist item	Location where item is reported
		This study aimed to elucidate the sources of heterogeneity and investigate potential moderator variables influencing the effect sizes of inflammatory biomarkers (CRP, IL-6, and TNF-α), using meta-regression analyses. The meta-regression with random effects employed residual restricted maximum likelihood (REML) for assessing inter study variance(τ^2), with the method of moments utilized for τ^2 estimation. Potential moderators investigated included exercise type, intensity, length, frequency, depression severity, age, and proportion of female participants to investigate their effects on distinct inflammatory factors. Subsequently, all covariates were simultaneously incorporated into a unified statistical model by pooling data from all three inflammatory biomarkers, thereby increasing the total sample size (k = 23) and enhancing statistical power for evaluating the independent contributions of each covariate.	
	13e	We conducted subgroup analyses by exercise modality, intensity, length and intervention duration.<br>Meta-regression was used to explore continuous moderators. Potential moderators investigated included exercise type, intensity, length, frequency, depression severity, age, and proportion of female participants to investigate their effects on distinct inflammatory factors. Subsequently, all covariates were simultaneously incorporated into a unified statistical model by pooling data from all three inflammatory biomarkers, thereby increasing the total sample size (k = 23) and enhancing statistical power for evaluating the independent contributions of each covariate.	p.5, section 2.5 (Data Synthesis and Analysis)
	13f	Leave-one-out sensitivity analysis was performed to explore potential sources of heterogeneity.	p.5, section 2.5 (Data Synthesis and Analysis)
Reporting bias assessment	14	Publication bias was assessed via funnel plots (⩾10 studies).	p.5, section 2.5 (Data Synthesis and Analysis)
Certainty assessment	15	To gauge the robustness of our approach, we utilized the RoB 2.0 assessment tool. The analysis was meticulously reviewed by our two researchers, with the outcomes thoroughly deliberated upon. Drawing from the Cochrane Collaboration's criteria for determining the risk of bias outlined in the Cochrane Handbook for Systematic Reviews of Interventions, we classified the studies into distinct categories based on the level of bias. In the two scenarios previously mentioned, the assessment should be classified as "low risk." If a study had any domains rated as having a "high risk of bias," it was classified as low-quality research. Those with one or more "unclear risk of bias" assessments fell into the moderate reliability category, while studies scoring "low risk of bias" across all domains were regarded as top-tier in terms of quality.	p.10, section 3.4 (Risk of bias and quality assessment)
**RESULTS**			
Study selection	16a	The initial search yielded a total of 2833 potentially eligible reports. After duplicate entries were eliminated, 2228 titles and abstracts were carefully reviewed. In the next stage, 27 studies were chosen for the full-text reading; and of these, 14 studies were excluded, leaving 13 studies that fulfilled the inclusion criteria and were included in the present review as shown in Figure 1.	p.6, section 3.1 (The process of study selection) and Figure 1
	16b	Several studies appeared missing (e.g., M. L. Perez et al. 2020) any biomarker value and data can not be converted (e.g., Krogh J et al. 2014)	Figure 1
Study characteristics	17	The summary of the key characteristics of the 13 studies included in our review is presented in Table 1. And details of intervention delivery across studies are summarized in Table S2, including settings, participant residence, supervision modes, and implementation of group exercise. A total of 1004 subjects evenly split between the exercise group (n=502) and the control group (n=502), with 54.28% women in these studies. participant ages ranged from 18 to 72 years old. There were no significant differences in gender distribution or age range between the exercise and control groups. Diverse exercise interventions types were employed across the qualifying studies, including mixed exercise, traditional aerobic exercise (running, cycling and swimming), Mind-Body exercise (qigong, tai chi) and High-intensity interval exercise. Of the total interventions, 11 studies used aerobic exercise, 2 studies used anaerobic exercise. Exercise intensity was categorized as low, moderate, or high. The lengths of the exercise interventions ranged from 4 weeks to 48 weeks. The control group conditions reported were active and passive, considering usual care, wait-list, placebo intervention, stretching exercise, health education, patient-centered	p.7. section 3.2 (Characteristics of included studies)

Section and Topic	Item #	Checklist item	Location where item is reported
		counselling in the group. What is more, because the study aims to explore influence of exercise across different populations and the antidepressant effect of different exercise volume, the control group received a intervention of active week x passive week or 4KKW exercise volume. Most of the research used outcome indicators that are depression and anxiety scale, CRP, IL-6, and TNF-α levels.	
Risk of bias in studies	18	Risk of bias judgements for each study are shown in Figure 2 and Supplementary Table S3. Common concerns included missing outcome data and selection of the reported data.	Figure 2 and Supplementary Table S3
Results of individual studies	19	Our findings demonstrated that exercise significantly improved depressive symptoms (SMD = -0.59, 95%CI: [-0.82, -0.35], I^2 = 61.7%, $p < 0.0001$) (Figure.3). The funnel plot showed approximate symmetry, and sensitivity analysis indicated that excluding any single study retained statistically significant results. When excluding Kader et al , heterogeneity decreased to 30% (SMD = -0.49, 95%CI: [-0.67, -0.31], I^2 = 30%, $p < 0.0001$). The funnel plot and sensitivity analysis plot are shown in Supplementary Figure S1.<br><br>Among the 13 articles reviewed, three categories of peripheral biomarkers were reported, with CRP, TNF-α, and IL-6 being the most commonly reported. Meta-analysis revealed that exercise did not reduce the level of CRP, IL-6 and TNF-α (CRP, MD = -1.21, 95%CI: [-3.02, 0.60], I^2 = 92.8%, p = 0.07; TNF-α, MD = 0.73, 95%CI: [-0.98, 2.45], I^2 = 94.8%, p = 0.10; IL-6, MD = -0.59, 95%CI: [-1.33, 0.18], I^2 = 81.7%, p = 0.29)(Figure.3). The funnel plot and sensitivity analysis plot are shown in Supplementary Figure S2.	p.10. section 3.5 (Primary outcome)
Results of syntheses	20a	Our analysis revealed that 2 studies presented a low risk of bias, 7 studies had a medium risk, and 4 studies were flagged as having a high risk of bias, as depicted in Figure.2 and Supplementary Table S3.	p.10. section 3.5 (Primary outcome)
	20b	Among the 13 studies included in the meta-analysis, 2 studies did not provide detailed data on depression symptom scores, 11 studies suggested exercise alleviated depressive symptoms, with 8 studies showing statistical significance. Our findings demonstrated that exercise significantly improved depressive symptoms (SMD = -0.59, 95%CI: [-0.82, -0.35], I^2 = 61.7%, $p < 0.0001$) (Figure.3).<br><br>Among the 13 articles reviewed, three categories of peripheral biomarkers were reported, with CRP, TNF-α, and IL-6 being the most commonly reported. Meta-analysis revealed that exercise did not reduce the level of CRP, IL-6 and TNF-α (CRP, MD = -1.21, 95%CI: [-3.02, 0.60], I^2 = 92.8%, p = 0.07; TNF-α, MD = 0.73, 95%CI: [-0.98, 2.45], I^2 = 94.8%, p = 0.10; IL-6, MD = -0.59, 95%CI: [-1.33, 0.18], I^2 = 81.7%, p = 0.29)(Figure.3).<br><br>6 trials were incorporated into the meta-analysis of CRP, with 4 studies suggesting that exercise might reduce CRP levels, though only 1 study reached statistical significance. The pooled results showed no significant association between exercise and CRP levels (MD = -1.21, 95%CI: [-3.02, 0.60], I^2 = 92.8%, p = 0.07).<br><br>The meta-analysis of IL-6 encompassed 10 eligible studies: 7 trials suggested exercise might lower IL-6 levels, with 3 studies achieving statistical significance. Our study found no notably association between exercise and IL-6 levels (MD = -0.59, 95%CI: [-1.33, 0.18], I^2 = 81.7%, p = 0.10).<br><br>7 trials were incorporated into the meta-analysis of TNF-α: 1 study reported exercise increased TNF-α with statistical significance, while 1 study showed the opposite trend. The results indicated no significant association between exercise and TNF-α levels (MD = 0.73, 95%CI: [-0.98, 2.45], I^2 = 94.8%, p = 0.29).	p.10. section 3.5 (Primary outcome)
	20c	We conducted subgroup analyses based on factors including age, severity of depression, exercise type, exercise intensity, frequency, and length. Subgroup analyses revealed that the sample's age, severity of depression, exercise type, intensity and frequency demonstrated minimal to negligible impact on the exercise-induced changes in biomarker concentrations in the bloodstream. However, lasting 8-12 weeks is effective to reduce the level of TNF-α (n = 89; MD =-0.73, 95%CI: [-0.17, -1.30], I2 = 0, $p < 0.05$), as shown in Table 2. The forest plot of different subgroups on CRP, IL-6 and TNF-α are presented in Supplementary Figure S3, 4, 5.	p.12-13. section 3.6. (Subgroup analysis) and 3.7 (Meta-regression analysis)
	20d	Depressive symptom: Sensitivity analysis revealed that removing Kader2016a et al reduced I^2 to 0%, yielding MD=0.06 (95%CI: -0.08 to	p.10. section

Section and Topic	Item #	Checklist item	Location where item is reported
		0.20).<br>CRP: Sensitivity analysis revealed that removing Kader2016a et al reduced I² to 0%, yielding MD=0.06 (95%CI: -0.08 to 0.20).<br>IL-6: Sensitivity analysis confirmed robustness, as excluding any study did not alter the results.<br>TNF-α: Sensitivity analysis confirmed no statistically significant changes upon excluding any study.	3.5 (Primary outcome)
Reporting biases	21	Regarding depressive symptoms, typical publication bias was observed. For CRP, no significant publication bias was detected, though a small-study effect trend was present. In the case of IL-6, significant publication bias existed. Substantial publication bias was identified for TNF-α.	Supplementary Figure S1, S2
Certainty of evidence	22	Our analysis revealed that 2 studies presented a low risk of bias, 7 studies had a medium risk, and 4 studies were flagged as having a high risk of bias depicted in Figure.2 and Supplementary Table S3.	p.10. section 3.4 (Risk of bias and quality assessment)
**DISCUSSION**			
Discussion	23a	The antidepressant effects of exercise have been confirmed, and it may even exert potential protective effects, making it a viable adjunctive treatment for depression. Our findings demonstrate that exercise combined with pharmacological treatment can improve the severity of depressive symptoms in individuals with major depression. It is beyond doubt that exercise is beneficial for depressive disorder. An 11-year HUNT cohort study including 33,908 healthy adults showed that regular exercise helps to reduce the prevalence of depressive disorder, and 12% of potential depression cases could be averted if every participant engaged in at least 60 minutes of weekly exercise. However, the mechanisms underlying exercise-induced improvement in depressive severity remain unclear. Current researches suggest that exercise may alleviate depression through increased BDNF levels in brain regions such as the hippocampus and modulating neuroimmune responses in the brain. An animal study has shown that 4 weeks of aerobic running exercise significantly reduced the depressive-like behaviors of mice exposed to chronic unpredictable stress (CUS). This also led to a decline in the microglial numbers and brought about changes in their shape within three specific hippocampal regions, effectively rebalancing the M1/M2 microglial states. These neural effects were mirrored by shifts in the production and release of both pro-inflammatory and anti-inflammatory cytokines in CUS-exposed mice. The underlying mechanisms may likely involve elevations in peripheral tissue such as fat stores and muscle, as well as increased plasma adiponectin. Additionally, running exercise appears to boost the expression of AdipoR1 in the hippocampus and activate the AMPK-NF-κB/STAT3 signaling pathways. Furthermore, regular aerobic exercise can reprogram peripheral tryptophan metabolism by enhancing kynurenine aminotransferase (KAT) expression in skeletal muscle via the PGC-1α1 pathway. This adaptation promotes the conversion of kynurenine to the neuroprotective metabolite kynurenic acid, thereby lowering circulating kynurenine levels and limiting its entry into the brain. Through this shift, exercise helps reduce the generation of neurotoxic metabolites such as quinolinic acid, modulates immune responses, and confers neuroprotection. This mechanism mediates the potential process by which exercise improves depression.<br>Our findings revealed that exercise had no statistically significant effect on inflammatory cytokine levels. This finding aligns with the results obtained by previous research. One meta-analysis suggested that long-term exercise interventions do not significantly modulate baseline IL-6 and IL-1β. Moreover, a randomized controlled trials revealed that there was no clear dose-dependent relationship between 12-week exercise intervention and peripheral cytokine levels in patients with depression. The lack of efficacy might arise from the interplaies of multifactorial biological mechanisms. The effects of exercise on peripheral proinflammatory cytokines are influenced by several factors including exercise type (aerobic, anaerobic, mixed), intensity, volume, and subject-specific attributes (NSAID use, sex, age, diet, baseline activity, and comorbidities like obesity, diabetes, and autoimmune diseases). The variance in exercise parameters might directly shift the balance between anti-inflammatory classical IL-6Rα signaling and pro-inflammatory sIL-6R trans-signaling pathways. The pretreatment with medications (e.g., selective serotonin reuptake inhibitors) in the participants might mask any observable alterations in cytokine concentrations. Moreover, during exercise, skeletal muscle mobilizes multiple energy substrates, including branched-chain amino acids (BCAAs) and tryptophan (Trp). Muscle takes up and metabolizes these amino acids through a series of transporters (e.g., the L-type amino acid transporter (LAT) family) and metabolic enzymes (e.g., BCAT, BCKDH), thereby modulating the flux of the peripheral Trp–kynurenine (Kyn) pathway. Circulating BCAAs can compete with Trp and certain Kyn metabolites via the large neutral amino acid (LNAA) transport system, consequently altering the Trp/Kyn distribution within tissues and the brain. Experimental evidence indicates that BCAAs or other LNAAs can reduce kynurenic acid	p.17-19. section 4. (Discussion)

Section and Topic	Item #	Checklist item	Location where item is reported
		(KYNA) production—primarily by inhibiting Kyn uptake into tissues (transport competition), although direct inhibition of kynurenine aminotransferase (KAT) activity has also been reported under certain in vitro conditions. Such metabolic interplay may amplify peripheral inflammatory responses through a variety of pathways. Finally, the results of the studies included in this meta-analysis were all levels of peripheral inflammatory factors. There might be dissociation between central and peripheral cytokines. Thus, finding a reliable and sensitive method for central inflammation detection and analysis is still a challenge.<br>The results of subgroup analysis revealed that 8-12 weeks of exercise intervention significantly decreased TNF-α levels in patients with depression. However, the results of univariable regression indicated that studies with longer exercise length showed a higher level of TNF-α. Synthesizing above outcomes, the peak of exercise-induced improvement of TNF-α level appeared around 8-12 weeks in patients with depression. The temporal dimension of intervention efficacy on TNF-α exhibits nonlinear characteristics: short-term programs (median 9 weeks, range 4-15 weeks) is not sufficient to initiate anti-inflammatory adaptations, whereas excessive exercise load or exercise dosage can lead to the increase of chronic inflammation. If the exercise load and dosage are too high, the body will be in a state of chronic low-concentration inflammation for a long time, and its ability to inhibit the production of TNF-α will be weakened, which may be one of the reasons for the increase in TNF-α levels. This biphasic response pattern substantiates prior observations by Rose et al., who identified an 8-week minimal threshold (optimal 9-12 weeks) to elicit stable inflammatory marker adaptations, suggesting the existence of critical temporal windows for exercise-induced immunomodulation in depressive populations.<br>There is evidence that IL-6 exerts a dual regulatory role on TNF-α through dichotomous signaling: promoting TNF-α release in pro-inflammatory states, yet suppressing it via pathways like TLR/NF-κB inhibition in response to exercise. Exercise-induced IL-6 release is closely linked to the energy demand and metabolism of skeletal muscle. There might be a “U” shaped relationship between training load and basal IL-6 levels, with non-excessive training levels being associated with lower IL-6 levels in either sedentary or excessively exercising individuals. If the exercise volume is too low (e.g. walking), it cannot induce an IL-6 anti-inflammatory effect. On the other hand, if the exercise volume is too high, it might hinder or restrict the secretion of anti-inflammatory cytokines, which are needed to decrease the enhancement in TNF-α levels. Therefore, a suitable exercise volume can beneficially skew the function of IL-6 to the anti-inflammatory effect, which contributed to suppression of TNF-α. For the above reasons, there might be an appropriate exercise volume (8-12 week) for the improvement of enhanced level of TNF-α in patients with depression.	
	23b	First, lots of studies included in this meta-analysis focused on low-to-moderate intensity exercise, with fewer studies focused on high intensity exercise. Because of the small number of included RCTs, the data of this meta-analysis were collected from females, males, older subjects, subjects with various disease and so on. However, these inherent individual variabilities had impacts on results. The number of subjects in our research is limited, and subsequent large-scale clinical studies are still needed. Second, medication data for subjects in this meta-analysis were not collected. Some drugs used by subjects may have effects on the levels of peripheral proinflammatory factors and effect size of exercise. For the future studies, it is recommended to give extra consideration to the drugs used by subjects. Third, the depression severity of patients included in this meta-analysis were not graded. This may create variation in efficacy for the exercise intervention. For the future studies, it becomes important to classify patients according to the severity of depression.	p.19. section 4. (Discussion)
	23c	First, inability to fully adjust confounders (e.g., exact drug dose, diet); Moreover, restricted subgroup analyses due to high heterogeneity; Third, assumed linearity in meta-regression despite potential U-shaped cytokine response.	p.19-20. section 4. (Discussion)
	23d	Future studies should employ more robust methodologies, extend trial length, account for participants' medication use, and include proper control groups that don't involve exercise. Furthermore, the detection of integrating CNS biomarkers will be essential to explore the peripheral-central connect. Only through such comprehensive investigations can we truly establish if exercise's mental health benefits operate through inflammatory pathways.	p.20. section 5. (Conclusion)
**OTHER INFORMATION**			
Registration and protocol	24a	This review was registered in PROSPERO (CRD42024590612).	p.3. Section 2. (Materials and Methods)

PRISMA 2020 Checklist

Section and Topic	Item #	Checklist item	Location where item is reported
	24b	The protocol is available at https://www.crd.york.ac.uk/PROSPERO/view/CRD42024590612	p.3. Section 2. (Materials and Methods)
	24c	Amendments to the registered protocol included: (1) revision of the review title to better reflect the study scope, without altering the main objectives; (2) addition of two co-authors who contributed to data analysis and manuscript preparation; and (3) inclusion of meta-regression analysis in the statistical methods, which was not specified in the original protocol, to explore potential sources of heterogeneity. These amendments were made prior to conducting the relevant analyses and did not affect the core objectives of the review.	NA
Support	25	This research was funded by the National Natural Science Foundation of China (grant number 32000838); and the Chinese Universities Scientific Fund (grant numbers 2024JNPD002 and 2024058).	p.21. section Funding
Competing interests	26	The authors declare no conflicts of interest. The funders had no role in: the design of the study; the collection, analyses, or interpretation of data; the writing of the manuscript; or the decision to publish the results.	p.21. section Conflicts of Interest
Availability of data, code and other materials	27	The extracted data and analysis code that support the findings of this study are available from the corresponding author upon reasonable request. The full search strategies and risk-of-bias assessments are provided in the Supplementary Files.	p.21. section Data Availability Statement

From: Page MJ, McKenzie JE, Bossuyt PM, Boutron I, Hoffmann TC, Mulrow CD, et al. The PRISMA 2020 statement: an updated guideline for reporting systematic reviews. BMJ 2021;372:n71. doi: 10.1136/bmj.n71. This work is licensed under CC BY 4.0. To view a copy of this license, visit https://creativecommons.org/licenses/by/4.0/
